# Engineered *Aedes aegypti* JAK/STAT Pathway-Mediated Immunity to Dengue Virus

**DOI:** 10.1371/journal.pntd.0005187

**Published:** 2017-01-12

**Authors:** Natapong Jupatanakul, Shuzhen Sim, Yesseinia I. Angleró-Rodríguez, Jayme Souza-Neto, Suchismita Das, Kristin E. Poti, Shannan L. Rossi, Nicholas Bergren, Nikos Vasilakis, George Dimopoulos

**Affiliations:** 1 W. Harry Feinstone Department of Molecular Microbiology and Immunology, Bloomberg School of Public Health, Johns Hopkins University, Baltimore, Maryland, United States of America; 2 Department of Pathology and Center of Biodefense and Emerging Infectious Diseases, Center for Tropical Diseases, Institute for Human Infections and Immunity, The University of Texas Medical Branch, Galveston TX, United States of America; Colorado State University, UNITED STATES

## Abstract

We have developed genetically modified *Ae*. *aegypti* mosquitoes that activate the conserved antiviral JAK/STAT pathway in the fat body tissue, by overexpressing either the receptor Dome or the Janus kinase Hop by the blood feeding-induced vitellogenin (Vg) promoter. Transgene expression inhibits infection with several dengue virus (DENV) serotypes in the midgut as well as systemically and in the salivary glands. The impact of the transgenes Dome and Hop on mosquito longevity was minimal, but it resulted in a compromised fecundity when compared to wild-type mosquitoes. Overexpression of Dome and Hop resulted in profound transcriptome regulation in the fat body tissue as well as the midgut tissue, pinpointing several expression signatures that reflect mechanisms of DENV restriction. Our transcriptome studies and reverse genetic analyses suggested that enrichment of DENV restriction factor and depletion of DENV host factor transcripts likely accounts for the DENV inhibition, and they allowed us to identify novel factors that modulate infection. Interestingly, the fat body-specific activation of the JAK/STAT pathway did not result in any enhanced resistance to Zika virus (ZIKV) or chikungunya virus (CHIKV) infection, thereby indicating a possible specialization of the pathway’s antiviral role.

## Author Summary

Dengue has represented a significant public health burden for a number of decades, and given the lack of dengue-specific drugs and limited availability of licensed vaccine, new methods for prevention and control are urgently needed. Here, we investigated whether genetic manipulation of the mosquitoes’ native JAK/STAT pathway-mediated anti-DENV defense system could be used to render mosquitoes more resistant to infection. We generated *Ae*. *aegypti* mosquitoes overexpressing the JAK/STAT pathway components Dome and Hop under the control of a bloodmeal-inducible, fat body-specific vitellogenin (Vg) promoter. These genetically modified mosquitoes showed an increased resistance to DENV infection, likely because of higher expression of DENV restriction factors and lower expression of DENV host factors, as indicated by transcriptome analyses. Expression of the transgenes had a minimal impact on mosquito longevity; however, it significantly impaired the mosquitoes’ fecundity. Interestingly, bloodmeal-inducible fat body-specific overexpression of either Hop or Dome did not affect mosquito permissiveness to either ZIKV or CHIKV infection, suggesting a possible specialization of JAK/STAT pathway antiviral defenses. Thus, our study is the first to provide a proof-of-concept that genetic engineering of the mosquitoes’ JAK/STAT immune pathway can be used to render this host more resistant to DENV infection.

## Introduction

Despite decades of attempts at disease control, dengue remains a major mosquito-borne arboviral disease, causing an estimated 390 million infections annually [1]. Without drugs and with only limited availability of a licensed vaccine, vector control has remained the most important approach to reduce disease transmission.

Dengue virus (DENV: *Flavivirus*) is maintained in a population through a horizontal transmission cycle between *Aedes* mosquitoes and humans. After mosquitoes acquire an infectious bloodmeal, the virus needs to complete its infection cycle and end up in the mosquito’s salivary glands for transmission to occur. Three major DENV infection barriers have been described in various refractory *Aedes aegypti* strains. The midgut infection barrier does not allow the virus to establish infection after ingestion of an infectious bloodmeal, and the disseminated infection barrier does not allow the virus to escape from the midgut tissue and disseminate to other parts of the insect; salivary gland infection and escape barriers have also been described. If the virus can overcome these impediments, it can then be injected into a human host in the mosquito’s saliva, thus transmitting the disease [2]. The replication cycle of DENV from midgut to salivary glands in *Aedes* mosquitoes takes 7–14 days, but this time interval can vary depending on the mosquito and virus strains as well as the temperature [3–7].

The Janus kinase/signal transducer and activator of transcription (JAK/STAT) pathway is a conserved immune signaling pathway that regulates developmental processes and antiviral immunity in both mammals and insects. We have previously shown that the JAK/STAT pathway controls DENV infection in *Ae*. *aegypti* [8]. Transient activation of the JAK/STAT pathway through RNAi-mediated gene silencing of the protein inhibitor of activated STAT (PIAS) renders mosquitoes more resistant to DENV infection of the midgut, whereas silencing of the receptor Dome or the Janus kinase Hop renders the mosquitoes more susceptible to DENV infection [8].

The JAK/STAT pathway controls DENV infection as early as 3 days post-infectious bloodmeal (dpibm), suggesting that genetic engineering of the pathway for earlier activation after a bloodmeal might result in a DENV resistance phenotype, and therefore offers a likely strategy to reduce dengue transmission. Activation of the JAK/STAT pathway is triggered by cytokine binding to the extracellular domain of the receptor, Dome. This binding changes the conformation of Dome, resulting in a dimerization of the receptor and self-phosphorylation of the Janus kinase Hop. Activated Hop then phosphorylates the cytoplasmic tail of Dome to generate a docking site for the transcription factor STAT. Once STAT is recruited to the receptor, it is phosphorylated, leading to dimerization. Dimerized STAT is then translocated to nucleus to activate the transcription of JAK/STAT pathway-regulated genes [9]. The JAK/STAT pathway is also negatively regulated at various steps by the suppressor of cytokine signaling (SOCS) and PIAS proteins [10].

We hypothesized that activation of the JAK/STAT pathway prior to, or immediately upon DENV ingestion could significantly restrict virus infection, perhaps to a degree that would adversely affect DENV transmission. To activate the JAK/STAT pathway, we generated genetically modified *Ae*. *aegypti* that expressed Dome or Hop under the control of the bloodmeal-inducible, fat body-specific vitellogenin (Vg) promoter. These transgenic *Ae*. *aegypti* showed greater resistance to DENV infection than did wild-type (WT) mosquitoes, and they have enabled further characterization of the molecular interactions between DENV and the mosquito vector. Interestingly, while the JAK/STAT pathway-hyperactive mosquitoes showed increased resistance to two DENV serotypes (DENV2 and DENV4), transgenic pathway activation did not confer resistance to two other important arboviral pathogens, Zika virus (ZIKV: *Flavivirus*) and chikungunya virus (CHIKV: *Alphavirus*), suggesting that the mosquito’s innate immune system and the JAK/STAT pathway deal differently with different viruses.

## Materials and Methods

### Ethics statement

This study was carried out in strict accordance with the recommendations in the Guide for the Care and Use of Laboratory Animals of the National Institutes of Health. Mice were used only for mosquito rearing as a blood source, according to the approved protocol. The protocol was approved by the Animal Care and Use Committee of the Johns Hopkins University (Permit Number: M006H300). Commercially obtained anonymous human blood was used for DENV, CHIKV, and ZIKV infection assays in mosquitoes, and informed consent was therefore not required.

### Generation of transformation vector constructs and transgenic mosquitoes

**Fig 1:**
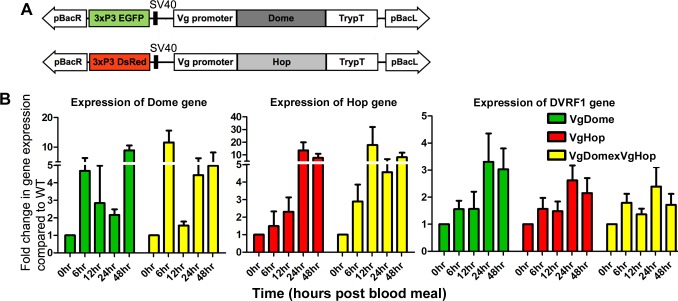
Generation of transgenic *Ae*. *aegypti* over-expressing Dome and Hop under the control of the Vg promoter. (A) Schematic of the piggyBac (pBac) transformation plasmids used to generate the VgDome and VgHop lines. pBacL, pBacR: pBac left and right arms, 3xP3: eye-specific promoter with either EGFP or DsRed as markers, Vg promoter: vitellogenin promoter, Dome: Dome coding sequence, Hop: Hop coding sequence, TrypT: trypsin terminator sequence. (B) Transcript abundance of transgenes and effector genes in the fat body of VgDome and VgHop lines from before blood feeding (0 hr) up to 48 hpbm. Each bar represents the relative fold change of Dome, Hop or DVRF1 (DENV restriction factor 1), compared between transgenic lines and WT *Ae*. *aegypti*. The S7 ribosomal gene was used to normalize cDNA templates. Error bars indicate standard error of the mean.

A schematic of the gene constructs used to generate the VgDome and VgHop transgenic *Ae*. *aegypti* lines is shown in Fig 1A. The *Ae*. *aegypti* Dome and Hop genes were PCR-amplified from *Ae*. *aegypti* cDNA using the primers listed in S6 Table and cloned downstream of the vitellogenin promoter [11]. *Ae*. *aegypti* Dome (AAEL012471) was PCR-amplified from cDNA in two segments: bp 1–1531 and bp 1532–3432; full-length Dome was then obtained through PCR using the Dome1F_PstI and Dome2R_*Pst*I primers, with equal proportions of each segment as template. Dome was cloned into the pBluescript II KS vector (Stratagene) at the *EcoR*V site. A 392-bp sequence from the putative terminator region of *Anopheles gambiae* trypsin was PCR-amplified from the vector pENTR-carboxypeptidase P-antryp1T [12,13] and cloned into pBluescript downstream of Dome at the *Xho*I/Klenow-filled site. A 2085-bp fragment from the promoter region of *Ae*. *aegypti* vitellogenin [11] was PCR-amplified from genomic DNA and cloned into pBluescript at the *Sma*I site upstream of Dome. The terminator sequence from the *An*. *gambiae* trypsin gene was cloned downstream of Dome. The AeVg-Dome-TrypT cassette was excised from pBluescript with *Fse*I and cloned into the *Fse*I site of the pBac[3xP3-EGFPafm] vector [14]. The resulting vector was used for embryo microinjections to generate the VgDome line.

*Ae*. *aegypti* Hop (AAEL012533) was PCR-amplified from cDNA in two segments: bp 1–1516 and bp 1517–3408. Each segment was separately cloned into pBluescript at the *EcoR*V site. The 5’ and 3’ segments were digested out with *EcoR*I/*Sac*I and *Sac*I/*Sal*I, respectively, and re-ligated into pBluescript at the *EcoR*I/*Sal*I sites to obtain full-length Hop. The trypsin terminator sequence was cloned at the *Xho*I/Klenow-filled site downstream of Hop, and the Vg promoter sequence was cloned at the *Xba*I/Klenow-filled site upstream of Hop. The AeVg-Hop-TrypT cassette was excised from pBluescript with *Fse*I and cloned into the *Fse*I site of the pBac[3xP3-DsRedafm] vector. The resulting vector was used for embryo microinjections to generate the VgHop line.

Embryo microinjections and initial screening for transformants were performed by the Insect Transformation Facility at the University of Maryland Biotechnology Institute using Orlando (Orl) strain *Ae*. *aegypti*. To generate the VgDome transgenic line, 565 embryos were injected with the transformation vector and the phsp-pBac helper plasmid. Of these, 279 survived to adults and were backcrossed to WT Orl adults in 19 pools. G1 larvae were screened for EGFP eye fluorescence (S1 Fig), and one pool was found to contain positives. Similarly, to generate the VgHop transgenic line, 613 embryos were injected with the transformation vector and the phsp-pBac helper plasmid. Of these, 132 survived to adulthood and were backcrossed to WT Orl adults in 10 pools. G1 larvae were screened for DsRed eye fluorescence (S1 Fig), and one pool was found to contain positives. Positive larvae were reared to adults and intercrossed to G5 to ensure homozygosity of the transgene. PCR confirmation of each line was performed with the VgPro R and ITRR2’ primers for the VgDome line and the AeVgPro R and DsRed S primers for the VgHop line (S2 Fig).

When characterizing the transgenic lines generated in the Orl background, we discovered that the Orl strain was in fact highly refractory to DENV infection [5]. Since the low levels of dengue infection in this strain would make it difficult to observe the impact of transgenic JAK/STAT activation on the virus, we undertook the additional step of backcrossing the transgene into the DENV-susceptible Rockefeller/UGAL (Rock) strain *Ae*. *aegypti* for five generations. After outcrossing with the Rock strain, both the VgDome and VgHop transgenic lines were bred within the same strain for another five generations to ensure homozygosity. The WT Orl strain was mated with the WT Rock strain in parallel to serve as a control. These Rock-introgressed VgDome, VgHop, and WT (OrlxRock) lines were used for subsequent gene expression analyses and infection experiments.

In an attempt to increase the induction of the JAK/STAT pathway, we crossed homozygous transgenic VgDome male mosquitoes with homozygous transgenic VgHop female mosquitoes in a ratio of 1:5 to generate a heterozygous hybrid VgDomexVgHop line overexpressing both Dome and Hop after blood feeding. All adult mosquitoes were maintained on 10% sucrose solution in a controlled environment at 27°C and 80% humidity with a 12 h light/dark cycle.

### Mosquito rearing, cell culture and virus strains

*Ae*. *aegypti* mosquito lines was reared using standard rearing procedures and mouse blood (BALB/c 028, Charles River Laboratories) was used for blood feeding.

The *Ae*. *albopictus* C6/36 cells (ATCC CRL-1660) were maintained in MEM medium (Gibco, USA) supplemented with 10% heat-inactivated FBS, 1% L-glutamine, 1% penicillin-streptomycin, and 1% MEM non-essential amino acids at 32°C and 5% CO_2_.

Baby hamster kidney cells (BHK-21, ATCC CCL-10) were maintained on DMEM medium supplemented with 10% FBS, 1% penicillin-streptomycin, and 5 μg/ml Plasmocin at 37°C and 5% CO_2_. Green monkey kidney (Vero) (Sigma-Aldrich) cells were maintained in DMEM with 5% FBS and 1% penicillin-streptomycin at 37°C and 5% CO_2._

DENV serotype 2 New Guinea C strain (DENV2), DENV serotype 4 strain Dominica/814669 (DENV4), ZIKV strain FSS 13025, and CHIKV strain 99659 were used as indicated in experiments.

### Oral virus infections in *Ae*. *aegypti* and virus titration

Mosquitoes were orally infected with DENV2 or DENV4 via artificial membrane feeding, as previously described [8,15]. In brief, C6/36 cells grown to 80% confluence were infected with DENV2, DENV4, or ZIKV at a multiplicity of infection (MOI) of 3.5 and incubated at 32°C and 5% CO_2_ for 6 days. The infected cells were then harvested and lysed through 3 cycles of freezing and thawing (between dry ice and a 37°C water bath). CHIKV was amplified on Vero cells at an MOI of 0.01 and harvested approximately 36 h later. The propagation yielded virus titers of 10^6^ to 10^7^ PFU/ml. The viruses were then mixed 1:1 v/v with commercial human blood and supplemented with 10% human serum and 1 mM ATP. The bloodmeal was offered to mosquitoes via an artificial membrane feeding system. Each experiment was performed in at least two to three biological replicates, as indicated. Plaque assays for DENV2 were performed in the BHK cell line, while CHIKV and ZIKV were titrated on Vero cell monolayers, and plaques were visualized by staining with 1% crystal violet. TCID50 assays for DENV4 were performed in C6/36 cells and visualized using peroxidase immunostaining, with monoclonal antibody 4G2 (a flavivirus group-specific monoclonal antibody) [16] as the primary antibody and a goat anti-mouse horseradish peroxidase (HRP) conjugate as the secondary antibody. All procedures involving DENV and ZIKV infections were performed in a BSL2 environment, and procedures involving CHIKV infections were performed in a BSL3 environment.

### Genome-wide oligonucleotide microarray transcriptomic analyses

To determine transcriptomic changes in the fat body and midgut after blood feeding in the transgenic VgDome and VgHop mosquito lines, RNA samples of the transgenic lines were compared to WT mosquitoes at 24 h post-naïve bloodmeal (hpbm) using Agilent-based oligonucleotide microarrays, as previously described [5]. Each transcriptomic comparison was performed in 3 or 4 biological replicates. In brief, pools of abdominal fat body or midgut tissue from 10–15 WT or transgenic mosquitoes were collected at 24 h after a naïve bloodmeal. We used 200 ng of total RNA from each pool to generate cy3- and cy5-labeled dCTP probes. Hybridizations were performed according to the manufacturer’s instructions, and the arrays were scanned with an Agilent SureScan microarray scanner; spot intensity was extracted using Agilent Feature extraction software. The expression data were processed and analyzed as described previously [5]. Self-self hybridizations have been used to determine a cutoff value on these microarrays for the biological significance of fold changes in transcript abundance: 0.75 on a log_2_ scale, which corresponds to a 1.68-fold regulation [17]. Numeric microarray gene expression data are presented in S1 Table (fat body transcriptomes) and S3 Table (midgut transcriptomes). Gene Expression Omnibus (GEO) accession number for raw microarray data is GSE90515.

Gene Ontology enrichment analysis was performed with the GOstats package in R (http://www.bioconductor.org/packages/release/bioc/html/GOstats.html) [18]. Over-representation of gene functional category based on previous classification [15] was performed using the hypergeometric test with the phyper package in R [19]. The results from the hypergeometric test are presented in S2 Table.

### RNA interference (RNAi)-mediated gene silencing

We used RNAi-mediated gene silencing to study the function of candidate host and restriction factors in WT mosquitoes as previously described [15]. Primers used to generate the dsRNAs are listed in S6 Table. EGFP dsRNA was used as a negative control for all experiments, and gene silencing efficiency was determined 3 days after dsRNA injection by using real-time PCR with gene-specific primers (S6 Table).

### Mosquito fitness assays

Mosquito longevity and fecundity assays were performed in three biological replicates as previously described [12]. Longevity assays with mosquitoes maintained on sucrose solution were performed with 3- to 4-day-old adult male or female mosquitoes. For the longevity assays involving JAK/STAT pathway activation, female mosquitoes were provided a single naïve human bloodmeal, followed by maintenance on a 10% sucrose solution. The number of dead mosquitoes was then monitored daily.

For the fecundity assays, 3- to 4-day-old adult female mosquitoes were fed on human blood via an artificial membrane. Fed mosquitoes were individually transferred to oviposition tubes, and the number of eggs laid was monitored until 5 days post-bloodmeal.

### Bacterial challenge

*Pantoea spp*. *and Bacillus cereus* isolated from a field site in Zambia [20] were used to represent Gram-negative and Gram-positive bacteria, respectively. Bacteria were cultured in Luria-Bertani (LB) medium at 30°C at 250 rpm for 12–14 h. Overnight cultures were washed twice with 1X PBS buffer, then resuspended in 1xPBS buffer to OD_600_ = 0.01. For bacterial challenge, we blood-fed mosquitoes with a naïve bloodmeal to activate the JAK/STAT pathway, then injected 69 nl of resuspended bacteria (approximately 400 bacteria per injection) into the thorax of each cold-anesthetized mosquito at 24 hpbm. Mosquitoes injected with 1X PBS were used as a negative control.

## Results

### Generation and validation of JAK/STAT pathway transgenic *Ae*. *aegypti*

To activate the JAK/STAT pathway in female mosquitoes upon bloodmeal acquisition, we generated the homozygous transgenic lines VgDome and VgHop, which over-express the pathway receptor Dome or the Janus kinase Hop under the control of the bloodmeal-inducible, fat body-specific Vg promoter (Fig 1A, S1 Fig). The Vg promotor has been shown to be activated after a bloodmeal and to reach its peak level of activity 24–48 h after blood ingestion [21]. *Aedes* mosquitoes usually acquire multiple bloodmeals during their gonadotropic cycle, especially when blood feeding is interrupted by a physical response from the host or probing in a non-optimal skin area [22–24], and we therefore hypothesized that transgene-mediated activation of the immune pathway by the selected promoter would likely prime the mosquito’s JAK/STAT-mediated anti-DENV defense for the next potentially infectious bloodmeal.

To generate a hybrid transgenic line over-expressing both Dome and Hop simultaneously, male homozygous VgDome and female homozygous VgHop were mated in a ratio of 1:5. The offspring were then screened for the expression of both EGFP and DsRed (S1 Fig), checked for transgenic Dome and Hop expression in the fat body, and used for subsequent experiments to test their susceptibility to DENV.

In the VgDome line, fat body expression of Dome was rapidly induced relative to WT mosquitoes, peaking as early as 6 h post-bloodmeal (hpbm), and again at 48 hpbm (Fig 1B). Dome induction in the hybrid line followed a similar pattern, albeit with an approximately 2-fold higher peak at 6 and 24 hpbm (Fig 1B). In the VgHop line, Hop expression was induced more gradually, peaking at 24 hpbm. Hop induction in the hybrid line followed a similar pattern, but with an earlier peak at 12 hpbm (Fig 1B). It is of course also possible that differences in transgene expression patterns could be due to position effects arising from transgene integration in different genomic locations.

Expression of dengue virus restriction factor 1 (DVRF1; AAEL008492), a putative anti-DENV effector molecule known to be transcriptionally regulated by the JAK/STAT pathway [8], peaked at 24 hpbm in all three lines (Fig 1B), indicating pathway activation. Interestingly, DVRF1 expression was not increased in the hybrid line (Fig 1B), suggesting that the pathway may have become maximally activated by each of the two transgenes, and that there might be limiting factors downstream of Dome and Hop.

### Transgenic activation of the JAK/STAT pathway inhibits DENV replication

**Fig 2:**
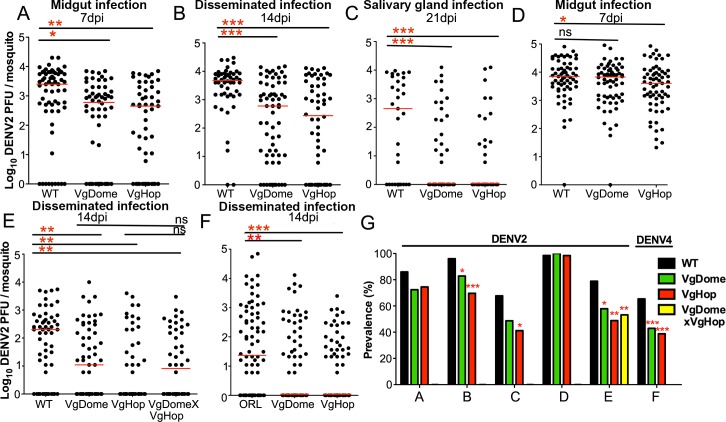
Effect of JAK/STAT pathway activation on DENV infection in transgenic *Ae*. *aegypti*. The JAK/STAT pathway was induced in the transgenic lines by providing them a naïve bloodmeal; 2 days later, JAK/STAT-activated mosquitoes were orally infected with DENV2 or DENV4. DENV2 titers of the VgDome and VgHop lines were determined for (A) midgut infection at 7 dpibm, (B) disseminated infection at 14 dpibm, and (C) salivary gland infection at 21 dpibm. (D) Midgut DENV2 infection without prior activation of the JAK/STAT pathway through a naïve blood meal at 7 dpibm. (E) Disseminated DENV2 infection of the JAK/STAT pathway-activated hybrid VgDomexVgHop line at 14 dpibm. (F) Disseminated DENV4 infection of the JAK/STAT-activated VgDome and VgHop lines at 14 dpibm. WT mosquitoes were used as a control in parallel in all experiments. Horizontal red lines indicate medians. (G) Prevalence of DENV infection represents data from graphs A-F. Data are pools of results from at least three replicates. Statistical analyses comparing median virus titers were performed using either the Mann-Whitney test or Kruskal-Wallis test with Dunn’s post-test, using Prism software. Statistical analyses comparing virus prevalence were determined by chi-square test. *: p<0.05, **: p<0.01, ***: p<0.001 compared to WT. Descriptive statistics is presented in supplementary S7 Table.

We first investigated the effect of the transgene-mediated activation of the JAK/STAT pathway on DENV infection. Mosquitoes were first fed a naïve bloodmeal to activate the JAK/STAT pathway; 2 days later, they were orally infected with DENV2 via a second (infectious) bloodmeal. We determined midgut infection at 7 dpibm (Fig 2A), disseminated infection at 14 dpibm (Fig 2B), and salivary gland infection at 21 dpibm (Fig 2C).

VgDome and VgHop mosquitoes showed significantly lower midgut DENV2 titers than did the WT mosquitoes (78.18% and 83.63% reduction in median titers for VgDome and VgHop, respectively). Both lines displayed an 87.37% (VgDome) and 94.21% (VgHop) reduction in median disseminated DENV2 titers; more importantly, the transgenic mosquitoes also displayed significantly lower virus titers in the salivary glands. Interestingly, the VgDome mosquitoes showed a significant reduction in DENV2 infection prevalence (percentage of mosquitoes with a detectable infection), but only at the stage of disseminated infection (13.74% reduction in prevalence), and not in the midgut or salivary glands (Fig 2G). VgHop mosquitoes had significantly lower levels of DENV2 prevalence at the disseminated infection stage (27.46% reduction) and in the salivary glands (39.14% reduction) but not in the midgut (Fig 2G).

Next, to determine if pathway activation at the point of DENV2 infection was sufficient for mediating systemic resistance, we omitted the initial naïve bloodmeal, and offered VgDome and VgHop mosquitoes a single DENV2-infected bloodmeal. Only the VgHop line showed significantly lower midgut DENV2 titers compared to WT (42.86% reduction in median titers); midgut titers in the VgDome line were not significantly different from WT (Fig 2D). DENV2 midgut prevalences in both lines without initial bloodmeal were comparable to WT (Fig 2G).

The hybrid line, which over-expresses both Dome and Hop, also displayed significantly reduced DENV2 titers and prevalence in the disseminated infection compared to WT when they were given a naïve bloodmeal before te DENV2 infection. However, this reduction was not significantly different from the homozygous VgDome and VgHop lines (Fig 2E and 2G). This result points to the possible existence of a limiting factor downstream of Dome and Hop, and is consistent with the induction patterns of DVRF1 transcripts described above. Since we observed no difference in DENV2 susceptibility between the hybrid and the VgDome and VgHop transgenic lines, we chose to use only the two homozygous lines for subsequent experiments.

Previous studies on the role of the JAK/STAT pathway during DENV infection in *Ae*. *aegypti* [8,25] have only been performed with DENV2. To determine if the inhibitory activity of the JAK/STAT pathway on DENV infection is conserved for different DENV serotypes, we challenged the VgDome and VgHop lines with DENV4 and assessed disseminated infection (Fig 2F and 2G). Both lines showed a significantly lower DENV4 titers and prevalence compared to WT mosquitoes.

### Impact of transgenic JAK/STAT pathway activation on mosquito fitness

Immune system activation and transgenic over-expression of certain immune-related genes have been associated with fitness trade-offs [26,27]; transgenic JAK/STAT activation may be particularly prone to this because the pathway also functions in insect development and other processes [28–30]. For this reason, we examined the impact of transgene expression on certain fitness parameters in our transgenic lines.

**Fig 3:**
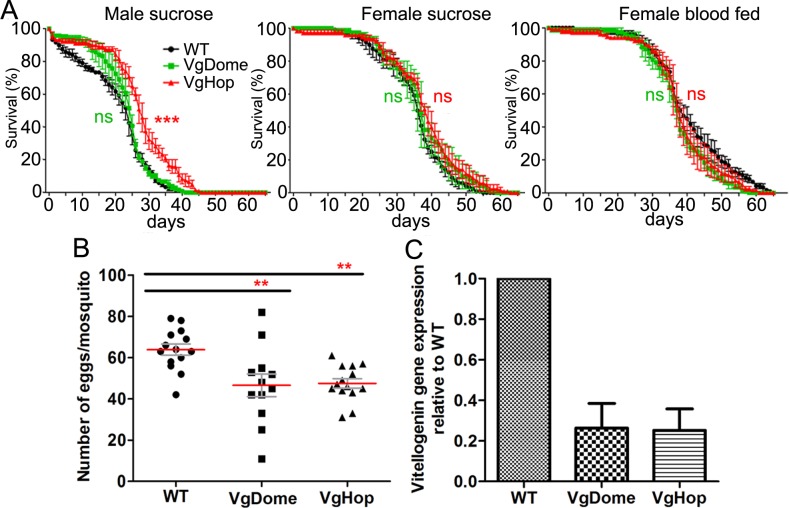
Impact of transgenesis on mosquito fitness. (A) Lifespans of male and female mosquitoes maintained on 10% sucrose solution, or of female mosquitoes that were provided a bloodmeal to induce transgene expression. Statistical analyses of survival curves was performed using the log rank test with Prism software. ***: p<0.001. (B) Fecundity of WT and transgenic *Ae*. *aegypti*, as represented by the number of eggs produced by each female mosquito. Statistical analyses were performed using the Mann-Whitney test with Prism software **: p<0.01 as compared to WT. (C) Expression of vitellogenin at 24 hpbm in the transgenic lines as compared to WT. mRNA levels were measured by real-time PCR, with ribosomal gene S7 as the normalization control. Error bars indicate standard error of the mean.

We first examined the impact of Dome and Hop transgenesis on the longevity of male and female mosquitoes maintained on 10% sucrose solution (i.e. without a bloodmeal that would induce transgene expression). In male mosquitoes, the longevity of the VgDome line was comparable to WT, while the longevity of the male VgHop line was greater (by 4 days) than WT (Fig 3A). The longevities of the female VgDome and VgHop lines were comparable to WT, suggesting a minimal impact of the transgenes on the mosquitoes’ life span in the absence of a bloodmeal.

We next examined the effect of bloodmeal-inducible transgene expression on female *Ae*. *aegypti* longevity. The longevity of the female VgDome and VgHop lines after blood feeding was comparable to WT, suggesting minimal fitness trade-offs in terms of mosquito life span when the JAK/STAT pathway is transiently activated.

The VgDome and VgHop lines both produced significantly fewer eggs than did WT mosquitoes (Fig 3B), suggesting that transgene expression compromises fecundity. The lower egg production may in part be due to competition between the endogenous and transgenic Vg promoters for transcriptional machinery such as transcription factors and RNA polymerase. This is supported by our observation of reduced Vg gene expression in transgenic mosquitoes compared to WT after blood feeding (Fig 3C).

### Impact of transgenic JAK/STAT pathway activation on the mosquito transcriptome

The JAK/STAT pathway-regulated antiviral effectors responsible for suppressing DENV infection are largely unknown, except for two genes, DVRF1 and DVRF2, that encode putative secreted and membrane-bound proteins, respectively, of unknown function [8]. In an effort to comprehensively characterize the impact of JAK/STAT activation, we used whole-genome oligonucleotide microarrays to compare fat body transcriptomes of the transgenic and WT lines, at 24 hpbm. DVRF1 expression peaks at this time point, suggesting peak JAK/STAT pathway activity. As expected, DVRF1 transcripts were enriched in both transgenic lines relative to WT (S1 Table), an indication of pathway activation.

**Fig 4:**
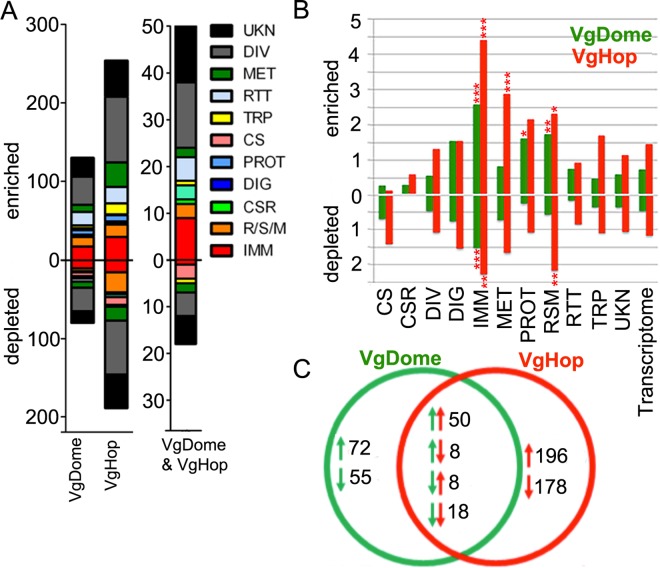
Fat body transcriptomic profiles of transgenic mosquitoes compared to WT at 24 hpbm. (A) Number of differentially expressed transcripts between the fat body of transgenic and WT mosquitoes., classified according to functional groups as previously described [8,15]. Abbreviations: CS, cytoskeletal and structural; CSR, chemosensory reception; DIV, diverse functions; DIG, blood and sugar food digestive; IMM, immunity; MET, metabolism; PROT, proteolysis; RSM, redox, stress, and mitochondrion; RTT, replication, transcription, and translation; TRP, transport; UKN, unknown functions. (B) Percentage of genes enriched or depleted, in the fatbody of transgenic lines compared to the WT, for each functional group (C) Venn diagram showing genes significantly regulated in VgDome and VgHop mosquitoes. Green arrows and circle represent the VgDome strain, and red arrows and circle represent the VgHop strain. Upward arrows represent genes significantly enriched, downward arrows represent genes significantly depleted in each strain when compared to WT mosquitoes.

The fat body transcriptomic analysis identified hundreds of JAK/STAT pathway-regulated transcripts belonging to various functional groups. Genes with diverse (DIV) and unknown (UKN) functions were particularly prominent (Fig 4A), which was not unexpected since the JAK/STAT pathway regulates a variety of biological processes. In VgDome mosquitoes, 130 transcripts (0.75% of the whole transcriptome) were enriched, and 71 (0.47%) were depleted compared to WT mosquitoes (Fig 4A and 4B). In VgHop, 254 transcripts (1.46%) were enriched compared to WT, and 204 (1.18%) were depleted (Fig 4A and 4B).

Only 50 transcripts were commonly enriched and 18 commonly depleted in the fat bodies of both the VgDome and VgHop transgenic lines compared to WT mosquitoes (Fig 4A and 4C). Functional pathway analysis using the GOstats package in R [18,31] revealed that gene ontology (GO) terms related to cell cycle regulation were over-represented among these 68 commonly regulated transcripts (Table 1). This small overlap suggests the presence of complex regulatory mechanisms, such that over-expression of different pathway components activates different subsets of genes.

**Table 1:** Gene ontology terms over-represented among transcripts commonly regulated in the fat body of VgDome and VgHop compared to WT. The analyses were performed using the GOstats package in R with a list of 50 commonly enriched and 18 commonly depleted transcripts. Gene ontology (GO) terms with p-values ≤0.01 were considered statistically significant.

Gene ontology ID	P-value	GO Term
GO:0051301	0	cell division
GO:0051246	0.002	regulation of protein metabolic process
GO:0010389	0.002	regulation of G2/M transition of mitotic cell cycle
GO:0000086	0.002	G2/M transition of mitotic cell cycle
GO:0044839	0.002	cell cycle G2/M phase transition
GO:1902749	0.002	regulation of cell cycle G2/M phase transition
GO:0007049	0.002	cell cycle
GO:0050790	0.003	regulation of catalytic activity
GO:0065009	0.003	regulation of molecular function
GO:0007126	0.004	meiotic nuclear division
GO:0051321	0.004	meiotic cell cycle
GO:0007346	0.008	regulation of mitotic cell cycle
GO:0044770	0.008	cell cycle phase transition
GO:0044772	0.008	mitotic cell cycle phase transition
GO:1901990	0.008	regulation of mitotic cell cycle phase transition
GO:0010564	0.008	regulation of cell cycle process
GO:1901987	0.008	regulation of cell cycle phase transition
GO:0060255	0.008	regulation of macromolecule metabolic process
GO:0006801	0.009	superoxide metabolic process
GO:0000079	0.01	regulation of cyclin-dependent protein serine/threonine kinase activity
GO:0071900	0.01	regulation of protein serine/threonine kinase activity

### Transgenic JAK/STAT pathway activation results in broad immune system stimulation

Because of the incomplete gene ontology assignment of *Ae*. *aegypti* transcripts in Vectorbase (i.e., several immune-related genes have not been assigned immunity-related ontologies), we also performed an over-representation analysis based on a previously annotated gene functional category list [15] using the phyper package in R [19,31]. In both transgenic lines, transcripts with immune-related (IMM) functions made up the largest specific class of regulated transcripts (excluding those with diverse [DIV] or unknown [UKN] function), and were significantly over-represented (Fig 4B and S2 Table). Of the 659 *Ae*. *aegypti* immune-related genes (IMM), 2.58% were induced and 1.52% were repressed in the VgDome line compared to WT; in the VgHop line, 4.40% of all IMM genes were enriched, and 2.28% were repressed. The percentage of regulated genes in the IMM category was 2 to 3-fold higher than the average percentage across all functional categories (S2 Table). These results emphasize the importance of the JAK/STAT pathway in mosquito immune regulation, and are consistent with our observations that the transgenic lines control DENV infection better than the WT. Several JAK/STAT pathway-induced IMM transcripts may encode potential DENV restriction factors (RFs), i.e. proteins that inhibit DENV replication in the mosquito.

Among the 50 genes commonly enriched in VgDome and VgHop, the IMM category represented the largest class (9 genes, 1.37% of the total IMM) (Fig 4A, S1 Table). These were: three C-type lectins (CTLs; AAEL005482, AAEL011610, and AAEL014390), three fibrinogen and fibronectin-related proteins (FBNs; AAEL006704, AAEL011400, and AAEL013417), two transferrins (TFs; AAEL015458, and AAEL015639), and a cathepsin b (CatB; AAEL015312). Super oxide dismutase (AAEL006271) was the only IMM gene among 18 genes commonly depleted in both lines.

Over-expression of Dome and Hop also regulated specific subsets of IMM transcripts (S1 Table). Eight IMM genes were enriched in VgDome but not in VgHop mosquitoes, including three serine proteases (AAEL003279, AAEL000030, and AAEL006434), two Niemann-Pick Type C2 molecules (AAEL012064, and AAEL004120), a cathepsin b (AAEL007599), and a lysozyme C (AAEL017132). Twenty IMM transcripts were enriched in VgHop but not in VgDome mosquitoes. These included four cathepsin b genes (AAEL009637, AAEL009642, AAEL007585, and AAEL012216); four serine proteases (AAEL007969, AAEL007006, AAEL015430, and AAEL003625); a thioester-containing protein (TEP22; AAEL000087); and several anti-microbial peptides (AMPs) such as cecropins (AAEL000621, AAEL000625), defensins (AAEL003832, AAEL003841), a gambicin (AAEL004522); and a lysozyme P (AAEL003723).

Upregulated IMM transcripts could potentially encode as-yet uncharacterized DENV restriction factors. FBNs, for example, are thought to play pattern recognition roles in *Drosophila* and in *Anopheles* mosquitoes [32–35], but their function in *Ae*. *aegypti* has yet to be elucidated. TEP22, which encodes a complement factor-like protein, was previously reported to be involved in the mosquito's anti-fungal response [9,32,36,37] but remains unstudied in the context of DENV infection. Finally, while the antiviral activities of AMPs from the defensin and cecropin families have previously been reported [38,39], the role of gambicin in anti-DENV defense remains unknown.

### Transgenic JAK/STAT pathway activation down-regulates putative DENV host factors

Hundreds of transcripts belonging to functional classes other than IMM were differentially expressed in the fat body of VgDome and VgHop lines compared to WT (Fig 4A), reflecting the JAK/STAT pathway’s important roles in other biological processes such as cell development and homeostasis, as well as lipid metabolism [9,36,37].

Transcript abundances of several previously reported putative DENV host factors (HFs; genes that facilitate virus replication in the host) were significantly depleted in the transgenic lines compared to WT mosquitoes (S1 Table); these included vacuolar ATP synthase subunit ac39 (vATPase-ac39; AAEL0011025), sterol carrier protein 2 (SCP2; AAEL012697), and DEAD-box ATP-dependent RNA helicase (DDX; AAEL004978). This suggests possible mechanisms for the increased resistance to DENV observed in these lines. Down-regulation of virus HFs may act in parallel with the induction of virus RFs to limit virus infection.

vATPase-ac39 transcripts were depleted 2.707 log_2_-fold in VgHop compared to WT mosquitoes. Knockdown of vATPase-ac39 and several other vATPase subunits, as well as chemical inhibition of vATPase activity with bafilomycin, have been shown to inhibit DENV replication in *Ae*. *aegypti* [40].

Sterol carrier protein 2 (SCP2; AAEL012697) transcripts were depleted 3.624 log_2_-fold in the VgHop line compared to WT; proteins of this family are thought to mediate intracellular trafficking of cholesterol and other lipids. Lipid metabolism is known to be influenced by the JAK/STAT pathway [36], and also plays important roles in the replication of DENV, an enveloped virus. DENV is thought to facilitate its own replication by altering the expression of lipid-binding proteins and enzymes involved in lipid biosynthesis, such as fatty acid synthases and Niemann-Pick type C protein family members [41,42].

Transcripts of the DDX gene were depleted 0.79 log_2_-fold in VgHop compared to WT. DDX gene family members are required for the replication of hepatitis C virus (HCV) [43,44], retroviruses [45,46], and Japanese encephalitis virus (JEV) [47]. DDX proteins are used by these viruses to regulate the translational machinery and for viral RNA transport to favor virus replication. However, the role of this gene family in the context of DENV infection in *Ae*. *aegypti* remains unstudied.

### Functional analysis of JAK/STAT pathway-regulated putative DENV restriction factors and host factors

Based on their expression patterns and previous reports of their function, we selected five candidate DENV restriction factors for further functional characterization through RNAi-mediated gene knockdowns: the immune-related genes FBN, TEP22, and gambicin, and two genes of unknown function, Ukn7703 and Ukn566 (Table 2). Ukn7703 (AAEL007703) encodes a putative secreted protein with a C-terminal beta-propeller domain distantly related to WD-40 repeats, which are involved in protein-protein interactions in several biological processes, including signal transduction [48]. Ukn566 (AAEL000566) is predicted to be a transmembrane protein with conserved cysteine positions; cysteine repeats have previously been reported to be important for the three-dimensional structure and function of receptor proteins such as LDL [49] and scavenger receptors [50].

**Table 2:** Candidate host or restriction factors (HFs or RFs) selected for functional characterization using RNAi-mediated gene silencing.

Gene ID	Gene name	Abbreviation	Putative role	Log2-fold difference in gene expression in the fat body compared to WT	
				VgDome	VgHop
**AAEL013417**	**fibrinogen and fibronectin**	**FBN**	**RF**	**1.584**	**1.708**
**AAEL000087**	**macroglobulin/complement (TEP22)**	**TEP22**	**RF**	**0.414**	**0.752**
**AAEL004522**	**gambicin**	**GAMB**	**RF**		**1.796**
**AAEL007703**	**conserved hypothetical protein**	**UKN7703**	**RF**	**3.215**	**3.352**
**AAEL000566**	**conserved hypothetical protein**	**UKN566**	**RF**	**0.946**	**1.748**
**AAEL004978**	**DEAD box ATP-dependent RNA helicase**	**DDX**	**HF**	**-0.699**	**-0.79**
**AAEL012697**	**sterol carrier protein-2, putative**	**SCP2**	**HF**		**-3.624**

We further selected two candidate host factors—DDX and SCP2—from among the depleted transcripts for further characterization (Table 2). The potential modes of action of these genes have been elaborated on in the previous section.

**Fig 5:**
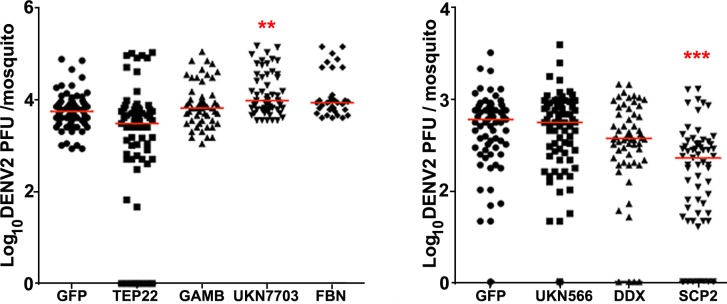
Effect of putative host and restriction factor silencing on DENV susceptibility. DENV2 titers at 14 dpibm in the disseminated infection (whole mosquito body except midgut) of WT *Ae*. *aegypti* after silencing of putative HFs or RFs, as compared to the GFP dsRNA-injected control. Data are pools of three biological replicates, and statistical analyses were performed using the Mann-Whitney test, **: p<0.01, ***: p<0.001 vs. WT mosquitoes. Descriptive statistics is presented in supplementary S8 Table.

Across the candidate genes, silencing efficiencies varied from 22% to 85% (S4 Fig). Our screen confirmed Ukn7703 as a putative DENV restriction factor (31.82% increase in median DENV titers when compared to the GFP dsRNA-injected group), and SCP2 as a putative host factor (85.71% decrease in DENV titers when compared to the GFP dsRNA-injected group) (Fig 5). Silencing of DDX also reduced DENV2 titers in the carcass by 61.43%, although this result was not statistically significant by a small margin (p = 0.0555) (Fig 5). This may be a result of the lower silencing efficiency (22%) achieved for this gene.

### Transgenic activation of the JAK/STAT pathway in the fat body regulates the midgut transcriptome

**Fig 6:**
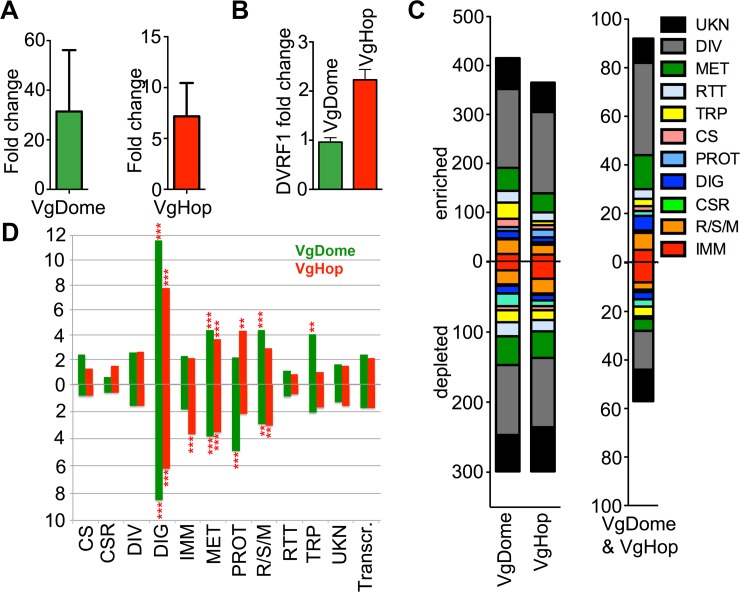
Midgut transcriptomic profiles of transgenic mosquitoes compared to WT at 24 hpbm. Relative gene expression of (A) Fold change in Dome and Hop gene expression (fat body/midgut) (B) Fold change in DVRF1 gene expression in the midgut of the transgenic lines as compared to WT. (C) Number of differentially expressed transcripts in the midgut of the transgenic lines as compared to WT mosquitoes, classified according to functional groups as previously described [8,15]. Abbreviations: CS, cytoskeletal and structural; CSR, chemosensory reception; DIV, diverse functions; DIG, blood and sugar food digestive; IMM, immunity; MET, metabolism; PROT, proteolysis; RSM, redox, stress, and mitochondrion; RTT, replication, transcription, and translation; TRP, transport; UKN, unknown functions. (D) Percentage of genes enriched or depleted in each functional group in the midguts of the VgDome or VgHop mosquitoes as compared to WT.

An interesting feature of the VgDome and VgHop transgenic lines was that activation of the JAK/STAT pathway in the fat body also restricts DENV2 infection in the midgut. This is unlikely to be due to leaky activation of the Vg promoter in the midgut, since both transgenes were induced to much higher levels in the fat body compared to the midgut (31-fold for Dome; 7-fold for Hop) (Fig 6A). Further, DVRF1 transcripts were not bloodmeal-induced in VgDome midguts, and induced only two-fold in VgHop midguts (Fig 6B). To further investigate this, we again used whole-genome oligonucleotide microarrays to compare the midgut transcriptomes of transgenic and WT mosquitoes, at 24 hpbm.

Intriguingly, Vg promoter-driven JAK/STAT activation regulated the expression of a larger number of transcripts in the midgut than in the fat body (Fig 6C, S3 Table): 415 transcripts (2.39% of the transcriptome) were enriched and 299 (1.72% of the transcriptome) depleted in VgDome midguts compared to WT; 365 transcripts (2.1% of the transcriptome) were enriched and 299 (1.72% of the transcriptome) depleted in VgHop midguts compared to WT (Fig 6C and 6D). Among these, 92 were commonly enriched and 57 commonly depleted in the midguts of both transgenic lines (Fig 6C). GO representation analysis of commonly depleted transcripts indicated over-representation of genes involved in proteolysis, protein metabolic processes, and lipid localization and transport, while no gene ontology was significantly over-represented in the enriched genes (Table 3).

**Table 3:** Gene ontology terms over-represented among transcripts commonly depleted in the midgut of VgDome and VgHop compared to WT. The analyses were performed using the GOstats package in R with a list of 57 transcripts commonly depleted in the midguts of VgDome and VgHop mosquitoes as compared to WT mosquitoes. Gene ontology (GO) terms with p-values ≤0.01 were considered statistically significant.

Gene ontology ID	P-value	GO Term
GO:0006508	0	proteolysis
GO:0019538	0.005	protein metabolic process
GO:0010876	0.01	lipid localization
GO:0006869	0.01	lipid transport

While IMM transcripts were over-represented in the fat body transcriptome, transcripts belonging to the digestion (DIG) functional category were over-represented in the midgut (Fig 6D). Certain putative host factors identified in the fat body transcriptome analysis were also depleted in the VgHop midgut; these included SCP2, and vATPase-ac39. VgDome midguts displayed a higher transcript abundance of Unk7703, a novel putative DENV restriction factor that was also induced in the fat body (S4 Table). These data suggest that JAK/STAT activation in the fat body can have a profound impact on distal organs, possibly through uncharacterized signaling mechanisms.

### Transgenic JAK/STAT pathway activation in fat body tissue does not affect susceptibility to bacteria, CHIKV, or ZIKV

Since JAK/STAT pathway activation resulted in the upregulation of numerous immune-related transcripts, we investigated whether transgenic mosquitoes also showed altered resistance to systemic bacterial infection. Independent transgenic mosquito cohorts were injected with either the Gram-negative bacterium *Pantoea spp*., the Gram-positive bacterium *Bacillus cereus*, or sterile PBS, after blood feeding. We found no resulting differences in mortality between the VgDome or VgHop lines and WT mosquitoes (S3 Fig). This is consistent with data from our previous study, in which transient silencing of PIAS, a negative regulator of the JAK/STAT pathway, had no effect on mosquito mortality upon bacterial infection [8]. It is possible that the regulated AMPs may have more specialized anti-DENV function or may not have anti-microbial activity against these particular bacteria. Similarly, a previous study of defensins from humans has also suggested that the anti-bacterial activity of certain AMPs is highly specific [51].

Further, since the JAK/STAT pathway is active against different DENV serotypes [52,53], we also investigated its role in mosquito immunity against CHIKV and ZIKV. Only the VgHop line was used in these experiments since it showed a more profound DENV resistance phenotype than the VgDome line.

**Fig 7:**
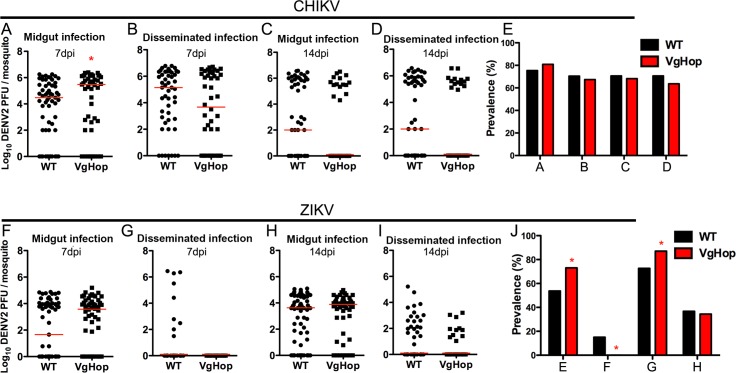
Effect of JAK/STAT pathway activation on CHIKV and ZIKV infection in the VgHop line. The JAK/STAT pathway was induced in the VgHop mosquitoes by providing them a naïve bloodmeal; 2 days later, JAK/STAT-activated mosquitoes were orally infected with CHIKV or ZIKV. CHIKV titers of the VgHop line were determined for (A) midgut infection at 7 dpibm, (B) disseminated infection at 7 dpibm, (C) midgut infection at 14 dpibm, and (D) disseminated infection at 14 dpibm. (E) Prevalence of CHIKV infection represents data from Fig 7A–D. ZIKV titers of the VgHop line were determined for (F) midgut infection at 7 dpibm, (G) disseminated infection at 7 dpibm, (H) midgut infection at 14 dpibm, and (I) disseminated infection at 14 dpibm. (J) Prevalence of ZIKV infection represents data from Fig 7F–I. Data are pools of results from 2 replicates. Statistical analyses comparing median virus titers were performed using the Mann-Whitney test with Prism software. Statistical analyses comparing virus prevalence were determined by chi square test. *: p<0.05 compared to WT mosquitoes. Descriptive statistics for CHIKV and ZIKV infection assays are presented in S5 Table.

VgHop mosquitoes were provided with a naïve bloodmeal to activate the pathway, then orally infected with CHIKV or ZIKV via a second bloodmeal, as done for DENV. We measured both midgut and disseminated infection at both 7 and 14 dpibm (Fig 7; descriptive statistics are presented in S5 Table).

At 7 dpibm, CHIKV titers were significantly higher in VgHop midguts compared to WT, but no differences in disseminated infection levels were observed (Fig 7A and 7B). At 14dpibm, infection levels did not differ between VgHop and WT in either tissue (Fig 7C and 7D). CHIKV infection prevalence did not differ between WT and VgHop cohorts at either time point or tissue (Fig 7E).

ZIKV infection intensity did not differ significantly between the VgHop and WT lines at either time point or tissue (Fig 7F–7I). While disseminated ZIKV prevalence was significantly reduced at 7 dpibm in VgHop mosquitoes as compared to WT, this difference was absent by 14 dpibm (Fig 7J). Interestingly, in both the transgenic and WT lines, ZIKV disseminated much less efficiently from the midgut as compared to DENV and CHIKV (Fig 7J).

Taken together, these data suggest that the antiviral activity of transgenic JAK/STAT pathway activation is restricted to DENV2 and DENV4.

## Discussion

While previous studies have linked the JAK/STAT pathway with DENV restriction in *Ae*. *aegypti*, the biology and translational potential of this relationship remains poorly understood. To examine the impact of JAK/STAT pathway activation on mosquito biology and identify possible genes and mechanisms mediating the inhibition of DENV infection, we generated transgenic *Ae*. *aegypti* that activated the JAK/STAT pathway in the fat body after bloodmeal. We reasoned that this spatially and temporally controlled JAK/STAT pathway activation, as opposed to the more random RNAi-mediated activation, would enable a much more detailed analysis.

Over-expression of Dome and Hop in the fat body prior to viral challenge controls DENV infection both in the midgut and systemically. Activation of the JAK/STAT pathway via a naïve bloodmeal prior to DENV exposure is required to maximize systemic resistance to the virus. In the absence of this immune pre-activation, only the VgHop line showed an increased resistance to midgut infection, and this effect was not as profound as when a naïve bloodmeal was provided. Pre-activation may boost the insect’s anti-viral defense, perhaps by priming uninfected cells and/or by maximizing the concentration of anti-viral effectors.

Besides its impact on DENV, transgenic JAK/STAT activation also profoundly affected the mosquito’s transcriptome, resulting in differential expression of hundreds of transcripts in the fat body and midgut. This reflects the pathway’s known involvement in a variety of biological processes, including development, cell proliferation, lipid homeostasis, and immunity.

In the fat body, the site of Dome and Hop over-expression, genes implicated in cell cycle regulation and kinase activity were over-represented. Most differentially regulated transcripts were specific to either Dome or Hop over-expression; these line-specific gene subsets suggest further complexities in JAK/STAT pathway regulation, such as novel branches, fine-tuning mechanisms, and multiple roles for the two transgenes.

That profound transcriptomic changes, along with increased resistance to DENV, were also observed in the midgut despite transgene over-expression in the fat body suggests possible JAK/STAT pathway-mediated inter-tissue signaling and immune priming. The *Drosophila* JAK/STAT pathway exerts a similar systemic effect [5,54], and the mammalian JAK/STAT pathway also plays critical roles in systemic type I interferon-mediated antiviral responses [51,55]. Chakrabarti *et al*. recently demonstrated JAK/STAT pathway-mediated inter-tissue signaling in *Drosophila* [56], where septic injury triggers hemocytes to secrete the cytokine Upd3, which then activates the JAK/STAT pathway in the fat body and gut, resulting in gut stem cell proliferation and an antimicrobial response [56].

Our transcriptomic analyses provide insights into how JAK/STAT signaling may control DENV infection in the mosquito. JAK/STAT activation resulted in the broad induction of numerous transcripts encoding immune recognition and effector molecules, including several that have previously been shown to restrict DENV. In addition, functional pathway analyses revealed that digestion- and lipid transport-related transcripts were differentially regulated in transgenic mosquitoes; these processes have previously been shown to impact DENV replication [41,42,57]. Finally, JAK/STAT activation also down-regulated transcript abundances of several genes that have known or potential roles as DENV host factors. These include vATPase (required for viral genome entry into host cells), DDX (translation of viral proteins), and SCP2 (lipid trafficking and homeostasis). That the pathway appears to impact DENV through diverse mechanisms bodes well for its use in transmission control, since this reduces the likelihood of the virus populations evolving resistance.

Through RNAi-mediated gene silencing assays, we provided further evidence that UNK7703 and SCP2 function as novel putative DENV restriction and host factors, respectively. UNK7703 is also induced in *Wolbachia*-infected *Ae*. *aegypti* [39,58], and is conserved among *Aedes*, *Culex*, and *Anopheles* mosquitoes (S4 Fig). It encodes a putative secreted protein with a C-terminal beta-propeller domain that is distantly related to WD-40 repeats; we speculate that it may be involved in cell signaling. SCP2 encodes an intracellular sterol carrier protein that facilitates cholesterol uptake in *Ae*. *aegypti* cells [59]. Knockdown or chemical inhibition of SCP2 was recently shown to inhibit DENV replication in *Ae*. *aegypti* Aag2 cells [60]. Here we confirm, for the first time, a role for SCP2 as a DENV host factor *in vivo*. Taken together with our transcriptomic data, which revealed regulation of lipid transport processes in both transgenic lines, and with previous studies [41,42], these results emphasize important roles for lipid homeostasis during DENV infection in *Ae*. *aegypti*.

While our transcriptomic analyses have yielded interesting insights into JAK/STAT pathway biology, we recognize that immune signaling in a WT genetic context may differ from our transgenic setup. In this study, we have made an effort to further characterize several candidate genes (identified through analysis of our transcriptomic data) through gene silencing assays in WT mosquitoes; more studies of this nature are needed to better understand the role of the JAK/STAT pathway in natural settings.

Although transgenic JAK/STAT pathway activation in the fat body effectively controlled both DENV2 and DENV4, it had no effect against two other important arboviral human pathogens, the alphavirus CHIKV and flavivirus ZIKV. While more detailed studies are required, this suggests a more complex nature for *Ae*. *aegypti* defenses against different arboviruses, and cautions against generalizing certain pathways as pan-antiviral. In *Drosophila*, *hop* function is required but not sufficient for the activation of Drosophila C virus (DCV)-induced immune genes [53]; similarly, it is possible that the JAK/STAT pathway is involved in the mosquito response against CHIKV and ZIKV, but that additional signals or regulators are also required to efficiently limit infection. CHIKV, in particular, does not appear to activate, and may even suppress, classical insect immune pathways in mosquito cells [8,15,61], suggesting that the mosquito mounts a very different response to this virus compared to DENV. Finally, since CHIKV and ZIKV reached higher infection levels than DENV in our WT mosquitoes, the replication rate of each virus could also have affected the pathway’s efficiency at controlling infection.

It is important to evaluate the fitness impact of any potential transgenic strategy. While the transgenic and WT lines did not differ in longevity, it should be noted that the insects were maintained under laboratory conditions, with an abundant food supply and minimal environmental stress; further experiments will be necessary to fully evaluate the effect of transient JAK/STAT pathway activation on longevity in natural settings. Both transgenic lines showed impaired fecundity compared to WT mosquitoes. Reduced egg production has also been observed in transgenic *An*. *gambiae* lines in which the Vg promoter is used to drive gene expression [12]; the use of alternative fat body-specific promoters may help minimize this fitness disadvantage.

In sum, our transgenic mosquito lines have provided valuable insights into the biology of the JAK/STAT pathway and its anti-DENV action, and allowed the identification of novel putative host and restriction factors. Further, this study serves as a proof-of-concept that genetic engineering of the *Ae*. *aegypti* JAK/STAT pathway has potential to increase resistance to DENV and further development and optimization, prior to extensive field-testing, could contribute towards the development of novel dengue control strategies. For example, it may be possible to achieve improved or total resistance by expressing additional transgenes that block the virus through different mechanisms, and/or by using more effective promoters. Recently developed powerful mosquito gene-drive systems [62,63], used circumspectly, are likely to make it possible to spread pathogen resistance genes in mosquito populations in a self-propagating fashion, even at a certain fitness cost.

## Supporting Information

S1 Fig Fluorescence screening of VgDome, VgHop, and hybrid VgDomexVgHop transgenic lines. The VgDome and VgHop lines contain eye-specific EGFP and DsRed markers respectively; the hybrid line contains both markers.
(TIF)

S2 Fig PCR confirmation of the transgenic Ae. aegypti VgDome and VgHop lines. (A) PCR confirmation of the VgDome transgenic line. The arrow indicates the expected band at 3.6 kb. (B) PCR confirmation of the VgHop transgenic line. The arrow indicates the expected band at 2.9 kb. The following templates were used: Lane 2: Genomic DNA from the VgDome line; Lanes 3 and 9: Genomic DNA from WT *Ae*. *aegypti*; Lanes 4 and 10: No template; Lane 5: pBac[3xP3-EGFPafm-AeVg-Dome-TrypT] plasmid; Lane 8: Genomic DNA from the VgHop line; Lane 11: pBac[3xP3-DsRedafm-AeVg-Hop-TrypT] plasmid. Lanes 1, 6, 7: 1-kb ladder.
(TIF)

S3 Fig Mortality of the VgDome and VgHop lines from bacterial infection. Mosquitoes were challenged with *Pantoea spp*. or *Bacillus cereus*, with PBS as a negative control. Survival analysis was performed using the OIsurv package in R. Data are from three independent replicates.
(TIF)

S4 Fig Phylogenetic tree of orthologs of the AAEL007703 gene obtained from Vector base. URL: https://www.vectorbase.org/Multi/GeneTree/Image?gt=VBGT00190000016830
(TIF)

S5 Fig Silencing efficiencies for candidate RFs and HFs. (TIFF)

S1 Table Log2-fold values and functional groups of transcripts that are enriched or depleted in the fat body of VgDome or VgHop mosquitoes relative to WT mosquitoes. Functional group abbreviations: CS, cytoskeletal and structural; CSR, chemosensory reception; DIV, diverse functions; DIG, blood and sugar food digestive; IMM, immunity; MET, metabolism; PROT, proteolysis; RSM, redox, stress and mitochondrion; RTT, replication, transcription, and translation; TRP, transport; UKN, unknown functions.
(DOCX)

S2 Table Numerical data and hypergeometric statistics of over-representation analysis of gene functional category from fat body and midgut transcriptome of VgDome and VgHop lines. Statistical analyses were performed using a hypergeometric test with phyper in R.
(DOCX)

S3 Table Log2-fold values and functional groups of transcripts that are enriched or depleted in the midgut of VgDome or VgHop mosquitoes relative to WT mosquitoes. Functional group abbreviations: CS, cytoskeletal and structural; CSR, chemosensory reception; DIV, diverse functions; DIG, blood and sugar food digestive; IMM, immunity; MET, metabolism; PROT, proteolysis; RSM, redox, stress and mitochondrion; RTT, replication, transcription, and translation; TRP, transport; UKN, unknown functions.
(DOCX)

S4 Table Log2-fold values of the putative RFs and HFs in the midgut transcriptome of the transgenic lines. (DOCX)

S5 Table Descriptive statistics for CHIKV and ZIKV infection assays. (DOCX)

S6 Table List of primers used to generate the transgene constructs, dsRNA synthesis, and real-time PCR. (DOCX)

S7 Table Descriptive statistics for DENV infection assays in transgenic mosquitoes. (DOCX)

S8 Table Descriptive statistics for DENV infection assays in RF and HF gene silenced mosquitoes. (DOCX)
