# Supplementary figures and images for: Engineered Aedes aegypti JAK/STAT Pathway-Mediated Immunity to Dengue Virus
Source: PLoS Negl Trop Dis. 2017 Jan 12;11(1):e0005187. doi: 10.1371/journal.pntd.0005187 (PMC5230736; doi:10.1371/journal.pntd.0005187)

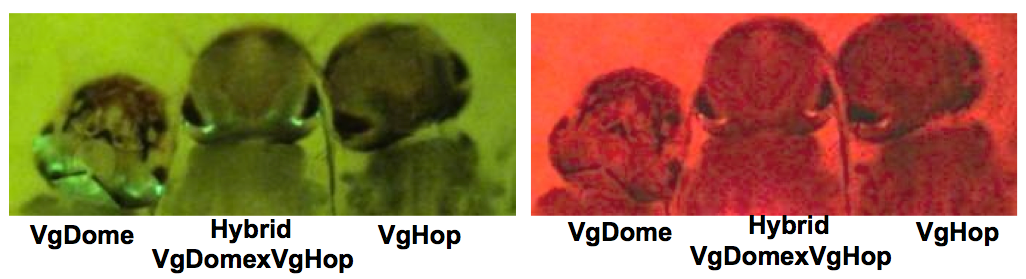

Supplement: S1 Fig — The VgDome and VgHop lines contain eye-specific EGFP and DsRed markers respectively; the hybrid line contains both markers. (TIF) [file pntd.0005187.s001.tif]

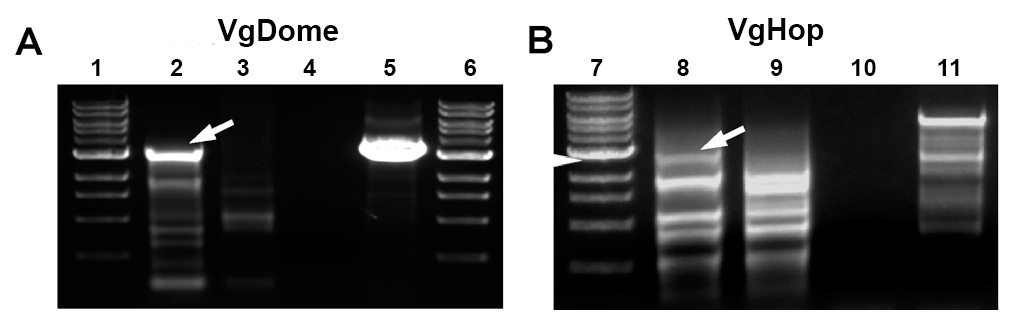

Supplement: S2 Fig — (A) PCR confirmation of the VgDome transgenic line. The arrow indicates the expected band at 3.6 kb. (B) PCR confirmation of the VgHop transgenic line. The arrow indicates the expected band at 2.9 kb. The following templates were used: Lane 2: Genomic DNA from the VgDome line; Lanes 3 and 9: Genomic DNA from WT Ae. aegypti; Lanes 4 and 10: No template; Lane 5: pBac[3xP3-EGFPafm-AeVg-Dome-TrypT] plasmid; Lane 8: Genomic DNA from the VgHop line; Lane 11: pBac[3xP3-DsRedafm-AeVg-Hop-TrypT] plasmid. Lanes 1, 6, 7: 1-kb ladder. (TIF) [file pntd.0005187.s002.tif]

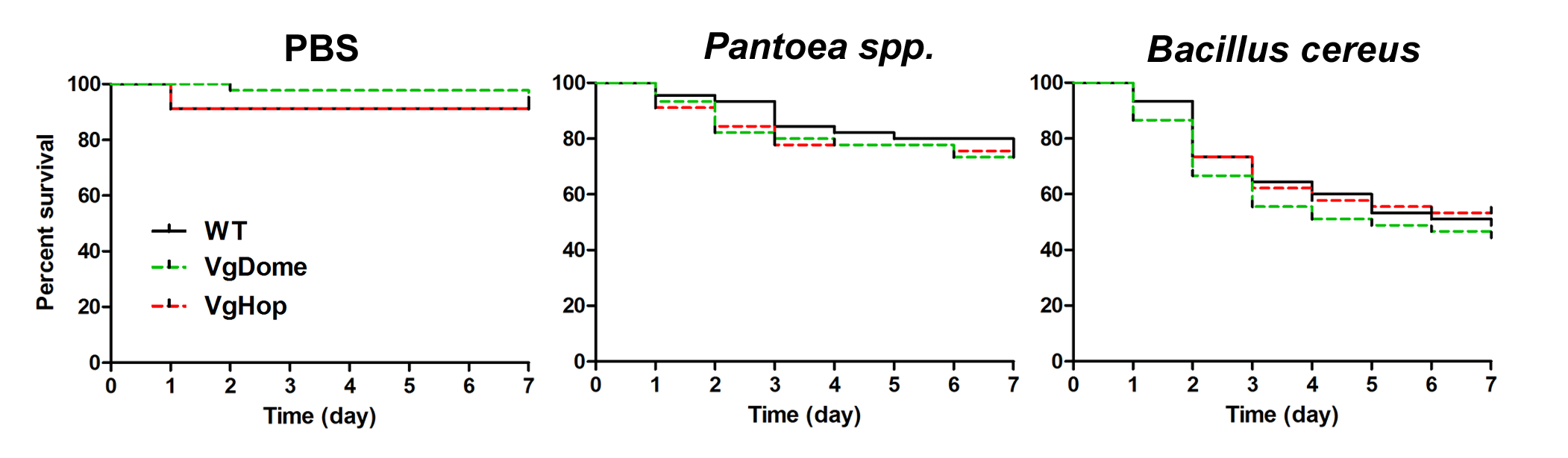

Supplement: S3 Fig — Mosquitoes were challenged with Pantoea spp. or Bacillus cereus, with PBS as a negative control. Survival analysis was performed using the OIsurv package in R. Data are from three independent replicates. (TIF) [file pntd.0005187.s003.tif]

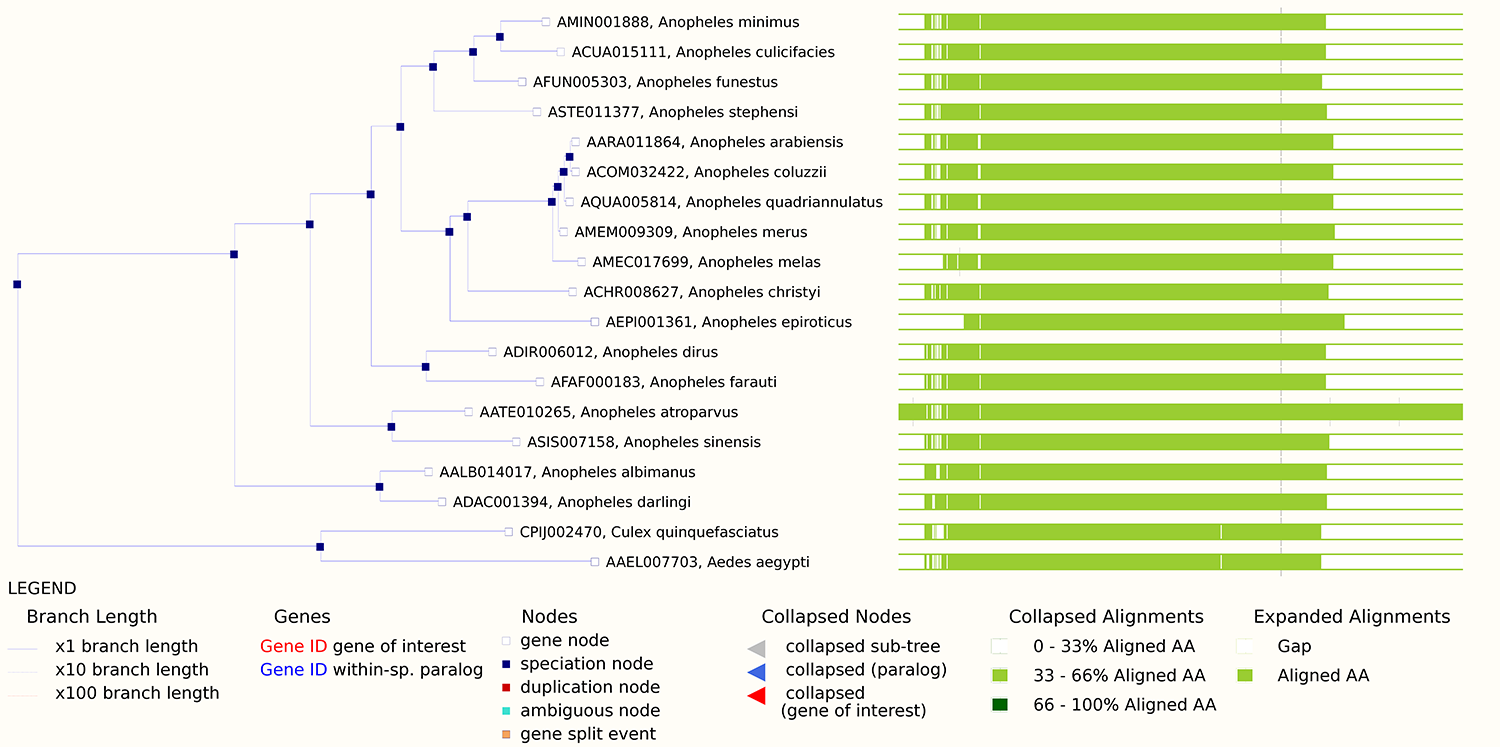

Supplement: S4 Fig — URL: https://www.vectorbase.org/Multi/GeneTree/Image?gt=VBGT00190000016830 (TIF) [file pntd.0005187.s004.tif]

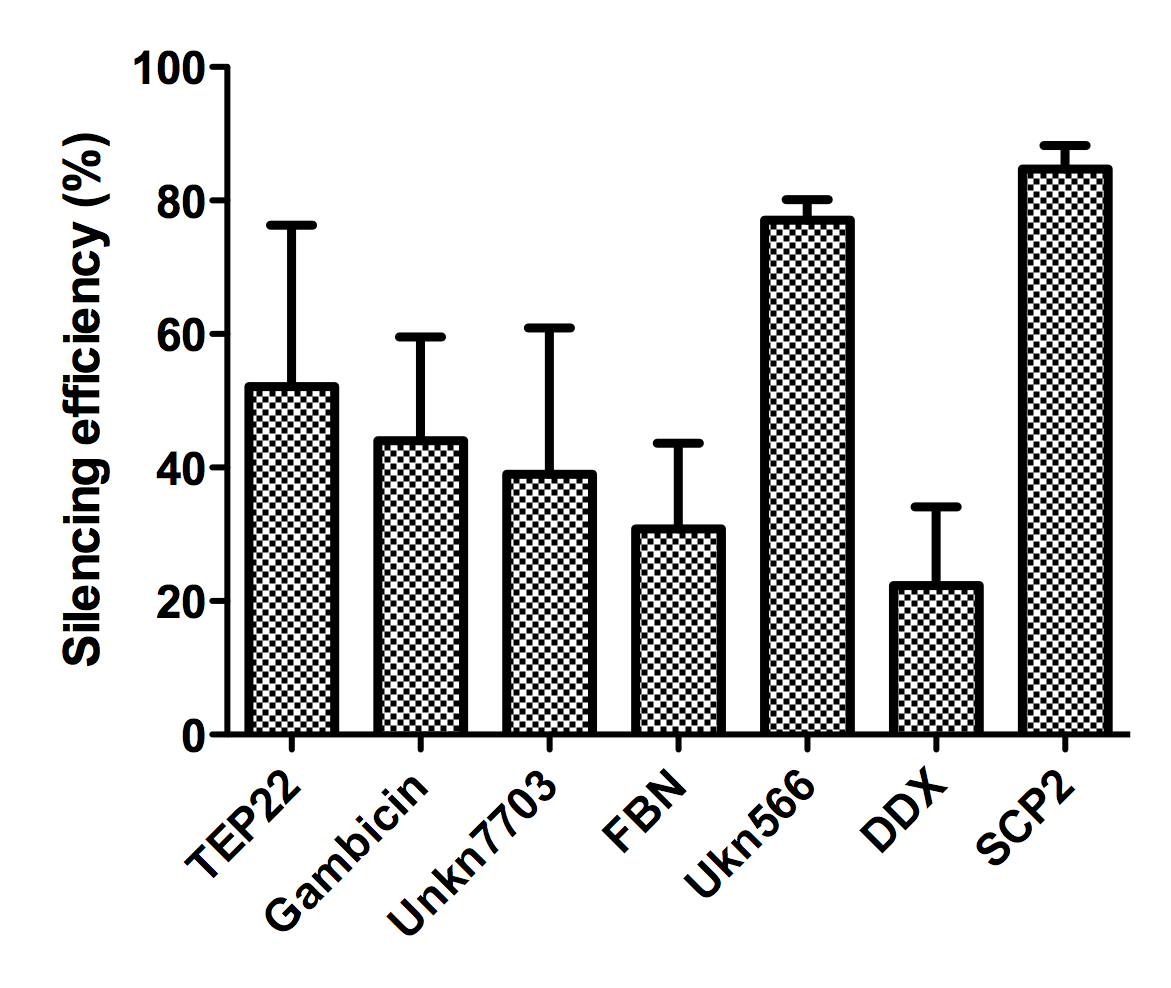

Supplement: S5 Fig — (TIFF) [file pntd.0005187.s005.tiff]
