# Supplementary material for: Engineered Aedes aegypti JAK/STAT Pathway-Mediated Immunity to Dengue Virus
Source: PLoS Negl Trop Dis. 2017 Jan 12;11(1):e0005187. doi: 10.1371/journal.pntd.0005187 (PMC5230736; doi:10.1371/journal.pntd.0005187)
Supplement: S1 Table — Functional group abbreviations: CS, cytoskeletal and structural; CSR, chemosensory reception; DIV, diverse functions; DIG, blood and sugar food digestive; IMM, immunity; MET, metabolism; PROT, proteolysis; RSM, redox, stress and mitochondrion; RTT, replication, transcription, and translation; TRP, transport; UKN, unknown functions. (DOCX) [file pntd.0005187.s006.docx]

**Table S1. Log_2_-fold values and functional groups of transcripts that are significantly enriched or depleted in the fat body of VgDome or VgHop mosquitoes relative to WT mosquitoes.** Functional group abbreviations: CS, cytoskeletal and structural; CSR, chemosensory reception; DIV, diverse functions; DIG, blood and sugar food digestive; IMM, immunity; MET, metabolism; PROT, proteolysis; RSM, redox, stress and mitochondrion; RTT, replication, transcription, and translation; TRP, transport; UKN, unknown functions.

| **Gene ID** | **Description** | **Functional group** | **VgDome FB** | **VgHop FB** |
| --- | --- | --- | --- | --- |
| AAEL000335 | lamin | CS | -0.971 | -0.84 |
| AAEL001904 | arp2/3 | CS |  | -0.773 |
| AAEL002185 | cuticle protein, putative | CS |  | -0.853 |
| AAEL002495 | conserved hypothetical protein (mucin-like protein) | CS |  | -1.65 |
| AAEL002759 | tropomyosin invertebrate | CS |  | -0.991 |
| AAEL004798 | conserved hypothetical protein (mucin-like protein) | CS |  | -1.569 |
| AAEL005146 | conserved hypothetical protein | CS | -1.023 |  |
| AAEL005417 | annexin x | CS |  | 0.964 |
| AAEL005426 | annexin x | CS | -1.442 |  |
| AAEL006726 | innexin | CS | -1.513 | -0.835 |
| AAEL009572 | cyclin B3 | CS | -0.784 | -0.818 |
| AAEL010094 | cyclin b | CS |  | -1.224 |
| AAEL012644 | conserved hypothetical protein | CS | 2.702 |  |
| AAEL013984 | structural constituent of cuticle | CS |  | -1.924 |
| AAEL017334 | Conserved hypothetical protein (chitin-binding domain type 2) | CS | 2.267 |  |
| AAEL003593 | hypothetical protein | CSR |  | 0.851 |
| AAEL005772 | Odorant-binding protein 99c, putative | CSR | 1.051 | 0.893 |
| AAEL000005 | hypothetical protein | DIV |  | -0.76 |
| AAEL000016 | conserved hypothetical protein | DIV | 1.064 |  |
| AAEL000079 | hypothetical protein | DIV |  | -0.769 |
| AAEL000105 | beta-alanine synthase, putative | DIV |  | -1.513 |
| AAEL000115 | conserved hypothetical protein | DIV |  | 0.864 |
| AAEL000125 | hypothetical protein | DIV |  | -0.762 |
| AAEL000147 | single-stranded DNA binding protein, putative | DIV |  | -0.903 |
| AAEL000159 | nipsnap | DIV |  | 1.073 |
| AAEL000262 | conserved hypothetical protein | DIV |  | -0.848 |
| AAEL000315 | pigeon protein (linotte protein) | DIV | 0.838 |  |
| AAEL000428 | tryptophan 2,3-dioxygenase | DIV |  | -1.939 |
| AAEL000442 | conserved hypothetical protein | DIV | -0.931 |  |
| AAEL000551 | hypothetical protein (pacifastin light chain [Culex quinquefasciatus]) | DIV |  | -1.886 |
| AAEL000776 | conserved hypothetical protein | DIV | -0.846 |  |
| AAEL000807 | Tetratricopeptide repeat protein, putative | DIV |  | 0.829 |
| AAEL000923 | conserved hypothetical protein | DIV |  | -0.829 |
| AAEL000973 | conserved hypothetical protein | DIV | -0.789 |  |
| AAEL001087 | synaptic vesicle protein | DIV |  | 1.109 |
| AAEL001100 | phosphoserine phosphatase | DIV |  | 1.23 |
| AAEL001287 | conserved hypothetical protein | DIV |  | 0.861 |
| AAEL001293 | conserved hypothetical protein | DIV |  | 1.057 |
| AAEL001307 | SEC14, putative | DIV |  | 1.183 |
| AAEL001352 | scaffold attachment factor b | DIV |  | -0.803 |
| AAEL001401 | conserved hypothetical protein | DIV |  | 1.052 |
| AAEL001607 | galactose-1-phosphate uridylyltransferase | DIV | 1.168 | 1.245 |
| AAEL001627 | UDP-n-acteylglucosamine pyrophosphorylase | DIV |  | 1.085 |
| AAEL001666 | nucleic acid binding, zinc ion binding | DIV | -1.265 |  |
| AAEL001667 | multicopper oxidase | DIV | 1.553 | 1.086 |
| AAEL001682 | nuclear movement protein nudc | DIV | 1.58 |  |
| AAEL001795 | orfY, putative | DIV |  | 0.818 |
| AAEL002048 | histidyl-tRNA synthetase | DIV |  | 0.969 |
| AAEL002125 | conserved hypothetical protein | DIV |  | -0.762 |
| AAEL002194 | uricase | DIV |  | -1.086 |
| AAEL002261 | GTP cyclohydrolase i | DIV |  | 0.968 |
| AAEL002473 | hypothetical protein | DIV | -0.777 |  |
| AAEL002501 | protein disulfide isomerase | DIV |  | 2.191 |
| AAEL002554 | anosmin, putative | DIV | -1.466 | -1.099 |
| AAEL002559 | conserved hypothetical protein | DIV | 1.526 |  |
| AAEL002675 | arginase | DIV |  | 0.775 |
| AAEL002764 | dihydrolipoamide succinyltransferase component of 2-oxoglutarate dehydrogenase | DIV |  | 0.764 |
| AAEL002860 | conserved hypothetical protein | DIV | 2.016 | 1.633 |
| AAEL002920 | hypothetical protein | DIV |  | -1.949 |
| AAEL002948 | frataxin, putative | DIV |  | -0.761 |
| AAEL003039 | nonsense-mediated mrna decay protein | DIV |  | 1.211 |
| AAEL003109 | atlastin | DIV |  | 1.988 |
| AAEL003165 | low molecular weight protein-tyrosine-phosphatase | DIV |  | 1.078 |
| AAEL003179 | protein arginine n-methyltransferase 1, putative | DIV |  | -0.857 |
| AAEL003213 | guanine deaminase | DIV | 0.813 |  |
| AAEL003237 | low molecular weight protein-tyrosine-phosphatase | DIV | 0.958 |  |
| AAEL003312 | hypothetical protein | DIV |  | 1.425 |
| AAEL003345 | argininosuccinate lyase | DIV | -1.081 |  |
| AAEL003371 | f-box and wd-40 domain protein | DIV |  | -0.816 |
| AAEL003385 | conserved hypothetical protein | DIV | -0.914 |  |
| AAEL003413 | f-spondin | DIV |  | 1.129 |
| AAEL003509 | smap1 | DIV |  | -0.805 |
| AAEL003581 | amidophosphoribosyltransferase | DIV |  | 0.803 |
| AAEL003599 | DNA binding, sulfiredoxin activity, oxidation reduction | DIV |  | 0.778 |
| AAEL003606 | purine biosynthesis protein 6, pur6 | DIV |  | 2.35 |
| AAEL003877 | ubiquitin | DIV | -0.916 |  |
| AAEL003980 | component of oligomeric golgi complex | DIV |  | -1.022 |
| AAEL004023 | Juvenile hormone-inducible protein, putative | DIV |  | 1.056 |
| AAEL004237 | vacuolar protein sorting 18 (deep orange protein) | DIV |  | 1.032 |
| AAEL004278 | conserved hypothetical protein | DIV |  | 1.592 |
| AAEL004335 | secreted ferritin G subunit precursor, putative | DIV |  | 1.225 |
| AAEL004392 | IAP-antagonist michelob-X-like protein, pro-apoptotic protein | DIV |  | 1.038 |
| AAEL004404 | HIG1 domain family member 2A, putative | DIV |  | 0.751 |
| AAEL004480 | cell division cycle 20 (cdc20) (fizzy) | DIV |  | 1.017 |
| AAEL004503 | conserved hypothetical protein | DIV |  | 1.134 |
| AAEL004547 | conserved hypothetical protein | DIV |  | 0.937 |
| AAEL004566 | myo inositol monophosphatase | DIV |  | -1.415 |
| AAEL004575 | beta-galactosidase | DIV |  | 0.809 |
| AAEL004613 | phenylalanyl-tRNA synthetase beta chain | DIV |  | 1.109 |
| AAEL004701 | argininosuccinate synthase | DIV |  | 0.976 |
| AAEL004813 | M-phase phosphoprotein, putative | DIV |  | 0.885 |
| AAEL004860 | acireductone dioxygenase | DIV | 1.969 |  |
| AAEL005199 | hypothetical protein | DIV |  | 1.41 |
| AAEL005289 | ornithine aminotransferase | DIV |  | 1.273 |
| AAEL005308 | pyruvate dehydrogenase | DIV | 0.933 | 1.36 |
| AAEL005348 | hypothetical protein | DIV | -0.928 |  |
| AAEL005384 | phosphoribosylformylglycinamidine synthase, putative | DIV |  | 2.203 |
| AAEL005457 | conserved hypothetical protein | DIV |  | 0.937 |
| AAEL005458 | carnitine o-acyltransferase | DIV | -0.783 | 0.776 |
| AAEL005558 | conserved hypothetical protein | DIV |  | 1.717 |
| AAEL005760 | hypothetical protein | DIV |  | 1.508 |
| AAEL005790 | malic enzyme | DIV | -0.935 |  |
| AAEL005976 | adenine phosphoribosyltransferase, putative | DIV | 1.025 |  |
| AAEL006023 | Vanin-like protein 1 precursor, putative | DIV |  | -0.98 |
| AAEL006279 | hypothetical protein | DIV |  | -0.955 |
| AAEL006353 | sulfotransferase (sult) | DIV | 1.528 |  |
| AAEL006446 | trehalose-6-phosphate synthase | DIV |  | -0.862 |
| AAEL006518 | cytidine deaminase, putative | DIV |  | -2.892 |
| AAEL006544 | nucleoporin P54 | DIV |  | -1.09 |
| AAEL006602 | hypothetical protein | DIV |  | 0.752 |
| AAEL006625 | conserved hypothetical protein | DIV | -1.063 | 2.651 |
| AAEL006662 | hypothetical protein | DIV | 1.212 | 2.29 |
| AAEL006712 | serine/threonine protein kinase | DIV |  | -0.897 |
| AAEL006909 | hypothetical protein | DIV |  | -0.928 |
| AAEL006972 | hepatocellular carcinoma-associated antigen | DIV | 1.463 |  |
| AAEL007072 | conserved hypothetical protein | DIV |  | -0.751 |
| AAEL007114 | conserved hypothetical protein | DIV |  | -0.79 |
| AAEL007130 | leucyl-tRNA synthetase | DIV | -1.176 |  |
| AAEL007226 | nidogen | DIV |  | 1.601 |
| AAEL007383 | secreted ferritin G subunit precursor, putative | DIV |  | -0.768 |
| AAEL007477 | ubiquitin-conjugating enzyme E2 i | DIV | -0.967 |  |
| AAEL007484 | protein transport protein sec23 | DIV | 0.809 | -0.805 |
| AAEL007494 | calcineurin b subunit | DIV | 0.771 | 0.791 |
| AAEL007557 | asparagine synthetase | DIV | 1.038 | 1.431 |
| AAEL007621 | conserved hypothetical protein | DIV |  | 2.049 |
| AAEL007686 | conserved hypothetical protein | DIV |  | 0.755 |
| AAEL007701 | conserved hypothetical protein | DIV |  | -0.85 |
| AAEL007702 | chaperonin | DIV | -0.868 |  |
| AAEL007767 | conserved hypothetical protein | DIV |  | 0.977 |
| AAEL007783 | centromere protein-A, putative | DIV |  | -1.078 |
| AAEL007828 | palmitoyl-protein thioesterase | DIV |  | 2.194 |
| AAEL007868 | ubiquinol-cytochrome c reductase complex 14 kd protein | DIV | 0.816 | 1.358 |
| AAEL008007 | conserved hypothetical protein | DIV |  | 0.955 |
| AAEL008076 | PIWI | DIV | -2.045 |  |
| AAEL008320 | conserved hypothetical protein | DIV |  | -0.754 |
| AAEL008431 | a kinase anchor protein | DIV | -1.011 | -0.919 |
| AAEL008473 | cysteinech venom protein, putative | DIV |  | -1.328 |
| AAEL008595 | conserved hypothetical protein | DIV | -1.162 | -0.953 |
| AAEL008598 | conserved hypothetical protein | DIV | 1.055 |  |
| AAEL008753 | conserved hypothetical protein | DIV |  | -1.126 |
| AAEL008789 | apolipophorin-III, putative | DIV | -0.792 | 0.865 |
| AAEL008863 | protein regulator of cytokinesis 1 prc1 | DIV |  | -1.226 |
| AAEL008953 | conserved hypothetical protein | DIV |  | -1.559 |
| AAEL009037 | GTP-binding protein (i) alpha subunit, gnai | DIV |  | -0.792 |
| AAEL009309 | lipid depleted protein | DIV |  | 1.381 |
| AAEL009508 | zinc finger protein | DIV |  | -0.852 |
| AAEL009629 | endoU protein, putative | DIV |  | -0.818 |
| AAEL009636 | conserved hypothetical protein | DIV |  | 0.876 |
| AAEL009652 | activin receptor type ii | DIV | 0.955 |  |
| AAEL009654 | hypothetical protein | DIV | -1.179 |  |
| AAEL009719 | conserved hypothetical protein | DIV |  | -1.359 |
| AAEL009859 | nucleolar GTP-binding protein | DIV | -1.567 |  |
| AAEL009931 | arsenite inducuble RNA associated protein aip-1 | DIV |  | 1.379 |
| AAEL009962 | hypothetical protein | DIV | -1.241 | -0.96 |
| AAEL009968 | hypothetical protein | DIV |  | -0.987 |
| AAEL010028 | sarcosine dehydrogenase | DIV |  | 0.922 |
| AAEL010065 | protein disulfide-isomerase A6 precursor | DIV | 2.233 |  |
| AAEL010097 | nuclein acid binding | DIV | -1.149 | -1.591 |
| AAEL010204 | dihydropyrimidine dehydrogenase | DIV |  | -1.28 |
| AAEL010280 | conserved hypothetical protein | DIV |  | 0.874 |
| AAEL010455 | cxyorf1 | DIV |  | -0.827 |
| AAEL010520 | conserved hypothetical protein | DIV |  | -0.778 |
| AAEL010572 | late endosomal/lysosomal MP1 interacting protein, putative | DIV |  | -1.297 |
| AAEL010656 | conserved hypothetical protein | DIV |  | 1.414 |
| AAEL010879 | conserved hypothetical protein | DIV |  | -1.093 |
| AAEL010943 | conserved hypothetical protein | DIV | -0.772 |  |
| AAEL011063 | tumor endothelial marker 7 precursor | DIV |  | 0.817 |
| AAEL011088 | conserved hypothetical protein | DIV |  | 0.757 |
| AAEL011105 | adducin | DIV | 1.201 |  |
| AAEL011135 | conserved hypothetical protein | DIV |  | -0.774 |
| AAEL011159 | cartilage associated protein | DIV |  | -0.836 |
| AAEL011168 | GTP-binding protein (i) alpha subunit, gnai | DIV |  | -0.75 |
| AAEL011264 | phosphatidylethanolamine-binding protein | DIV | 1.128 |  |
| AAEL011341 | apyrase, putative | DIV |  | -0.98 |
| AAEL011448 | conserved hypothetical protein | DIV | 1.044 |  |
| AAEL011452 | conserved hypothetical protein | DIV | 0.913 |  |
| AAEL011478 | cytoplasmic dynein light chain | DIV | -0.928 |  |
| AAEL011529 | late endosomal/lysosomal MP1 interacting protein, putative | DIV |  | -1.047 |
| AAEL011580 | conserved hypothetical protein | DIV |  | 0.786 |
| AAEL011849 | hypothetical protein | DIV | -0.79 |  |
| AAEL011853 | conserved hypothetical protein | DIV |  | -0.865 |
| AAEL011881 | conserved hypothetical protein | DIV | 0.897 | 0.867 |
| AAEL011892 | receptor for activated C kinase, putative | DIV |  | -1.134 |
| AAEL011980 | hypothetical protein | DIV |  | -0.84 |
| AAEL012233 | hypothetical protein | DIV |  | 1.117 |
| AAEL012260 | wdpeat protein | DIV | 1.298 |  |
| AAEL012417 | conserved hypothetical protein | DIV |  | 1.117 |
| AAEL012464 | alanine-glyoxylate aminotransferase | DIV |  | 1.099 |
| AAEL012502 | conserved hypothetical protein | DIV |  | -1.179 |
| AAEL012605 | conserved hypothetical protein | DIV | 2.008 | 1.699 |
| AAEL012632 | hypothetical protein | DIV | 0.93 |  |
| AAEL012851 | wdpeat protein | DIV | 0.903 | 1.017 |
| AAEL012855 | hypothetical protein | DIV | 1.019 | 3.375 |
| AAEL012856 | hypothetical protein | DIV |  | 1.083 |
| AAEL012939 | gamma-subunit,methylmalonyl-CoA decarboxylase, putative | DIV | -1.029 |  |
| AAEL013078 | glycosyltransferase | DIV |  | 0.764 |
| AAEL013334 | conserved hypothetical protein | DIV |  | 1.068 |
| AAEL013338 | lethal(2)essential for life protein, l2efl | DIV |  | -0.949 |
| AAEL013510 | smaug protein | DIV | -0.972 |  |
| AAEL013590 | conserved hypothetical protein | DIV |  | -0.854 |
| AAEL013596 | phosphatidylinositol 3-kinase regulatory subunit | DIV |  | -0.957 |
| AAEL013822 | protein binding | DIV |  | -0.927 |
| AAEL013844 | diazepam binding inhibitor, putative | DIV | 1.276 |  |
| AAEL013851 | conserved hypothetical protein (acetyltransferase (GNAT) family domain) | DIV | 1.233 | 2.448 |
| AAEL014199 | dihydropyrimidine dehydrogenase | DIV |  | -1.669 |
| AAEL014275 | molybdopterin cofactor sulfurase (mosc) | DIV |  | 0.787 |
| AAEL014310 | hypothetical protein | DIV |  | -0.852 |
| AAEL014561 | conserved hypothetical protein | DIV |  | 1.955 |
| AAEL014715 | 67 kDa polymerase-associated factor PAF67, putative | DIV | 0.883 |  |
| AAEL014852 | hypothetical protein | DIV | -0.862 |  |
| AAEL015375 | serine/threonine protein kinase | DIV |  | -0.905 |
| AAEL015631 | asparagine synthetase | DIV | 1.071 | 1.418 |
| AAEL015658 | conserved hypothetical protein | DIV |  | -1.281 |
| AAEL002969 | brain chitinase and chia | DIG |  | 1.381 |
| AAEL003060 | serine-type enodpeptidase, putative | DIG |  | 2.319 |
| AAEL005481 | alpha-glucosidase | DIG |  | -0.964 |
| AAEL006121 | Trypsin, putative | DIG |  | -0.788 |
| AAEL008080 | trypsin-eta, putative | DIG | 1.369 |  |
| AAEL013262 | conserved hypothetical protein | DIG | -1.001 |  |
| AAEL014361 | amidase | DIG | 0.782 |  |
| AAEL000030 | clip-domain serine protease, putative | IMM | 1.079 |  |
| AAEL000064 | dopachrome-conversion enzyme (DCE) isoenzyme, putative | IMM |  | 0.764 |
| AAEL000087 | macroglobulin/complement | IMM |  | 0.752 |
| AAEL000598 | antibacterial peptide, putative | IMM | -0.877 |  |
| AAEL000611 | antibacterial peptide, putative | IMM | -1.488 |  |
| AAEL000621 | antibacterial peptide, putative | IMM |  | 1.547 |
| AAEL000625 | antibacterial peptide, putative | IMM |  | 1.659 |
| AAEL002276 | serine protease, putative | IMM |  | -1.607 |
| AAEL002592 | hypothetical protein | IMM |  | -0.792 |
| AAEL003279 | clip-domain serine protease, putative | IMM | 0.953 |  |
| AAEL003625 | clip-domain serine protease, putative | IMM |  | 0.892 |
| AAEL003723 | lysozyme P, putative | IMM |  | 0.94 |
| AAEL003832 | conserved hypothetical protein | IMM |  | 1.168 |
| AAEL003841 | conserved hypothetical protein | IMM |  | 1.435 |
| AAEL004120 | Niemann-Pick Type C-2, putative | IMM | 0.832 |  |
| AAEL004401 | peroxinectin | IMM |  | -0.836 |
| AAEL004522 | gambicin | IMM |  | 1.796 |
| AAEL005431 | clip-domain serine protease, putative | IMM |  | -1.312 |
| AAEL005482 | conserved hypothetical protein | IMM | 2.369 | 2.205 |
| AAEL006168 | serine carboxypeptidase, putative | IMM |  | -0.822 |
| AAEL006271 | superoxide dismutase | IMM | -0.853 | -0.821 |
| AAEL006434 | serine protease, putative | IMM | 0.803 |  |
| AAEL006586 | serine protease | IMM | -0.976 |  |
| AAEL006704 | fibrinogen and fibronectin | IMM | 0.794 | 1.227 |
| AAEL006830 | yellow protein precursor | IMM | -0.839 | 1.7 |
| AAEL007006 | serine protease | IMM |  | 0.834 |
| AAEL007585 | cathepsin b | IMM | -0.853 | 1.184 |
| AAEL007599 | cathepsin b | IMM | 1.229 |  |
| AAEL007969 | serine protease | IMM |  | 0.789 |
| AAEL008607 | tep3 | IMM |  | -1.084 |
| AAEL009637 | cathepsin b | IMM |  | 1.513 |
| AAEL009642 | cathepsin b | IMM |  | 1.828 |
| AAEL011400 | conserved hypothetical protein | IMM | 2.634 | 2.21 |
| AAEL011446 | galactose-specific C-type lectin, putative | IMM |  | 1.316 |
| AAEL011610 | galactose-specific C-type lectin, putative | IMM | 0.767 | 0.796 |
| AAEL011616 | serine protease, putative | IMM | -0.956 |  |
| AAEL012064 | Niemann-Pick Type C-2, putative | IMM | 0.808 |  |
| AAEL012092 | leucinech repeat | IMM |  | 1.094 |
| AAEL012216 | cathepsin b | IMM | -0.776 | 1.181 |
| AAEL012251 | low-density lipoprotein receptor (ldl) | IMM |  | -0.805 |
| AAEL012471 | protein tyrosine phosphatase, putative | IMM |  | -0.979 |
| AAEL012711 | trypsin, putative | IMM |  | 1.502 |
| AAEL013417 | fibrinogen and fibronectin | IMM | 1.584 | 1.708 |
| AAEL014004 | clip-domain serine protease, putative | IMM | -0.761 |  |
| AAEL014238 | aromatic amino acid decarboxylase | IMM |  | -0.795 |
| AAEL014349 | serine protease | IMM |  | -1.681 |
| AAEL014385 | conserved hypothetical protein | IMM | -1.179 |  |
| AAEL014390 | galactose-specific C-type lectin, putative | IMM | 0.76 | 0.763 |
| AAEL014755 | tep2 | IMM |  | -0.957 |
| AAEL015312 | cathepsin b | IMM | 1.105 | 0.823 |
| AAEL015430 | serine protease, putative | IMM |  | 0.905 |
| AAEL015458 | transferrin | IMM | 0.874 | 2.292 |
| AAEL015639 | transferrin | IMM | 1.24 | 1.726 |
| AAEL017132 | C-Type Lysozyme (Lys-C). [Source:Aedes_ManualAnnotation;Acc:AAEL800171] | IMM | 1.146 | -1.13 |
| AAEL017325 | Clip-Domain Serine Protease, family B. (Truncated Protease). [Source:Aedes_ManualAnnotation;Acc:AAEL800831] | IMM |  | -0.978 |
| AAEL017536 | Holotricin, Glycine Rich Repreat Protein (GRRP), Anti-Microbial Peptide. [Source:Aedes_ManualAnnotation;Acc:AAEL800434] | IMM | 0.801 |  |
| AAEL010125 | mitotic protein phosphatase 1 regulator, putative | IMM |  | -1.094 |
| AAEL000006 | phosphoenolpyruvate carboxykinase | MET |  | 1.422 |
| AAEL000059 | proacrosin, putative | MET |  | 0.944 |
| AAEL000080 | phosphoenolpyruvate carboxykinase | MET |  | 1.135 |
| AAEL000101 | AMP dependent coa ligase | MET |  | 1.032 |
| AAEL000111 | nitrilase, putative | MET |  | 0.928 |
| AAEL001423 | acid phosphatase-1 | MET |  | 0.76 |
| AAEL001548 | glucosyl/glucuronosyl transferases | MET | 0.975 | 1.273 |
| AAEL001586 | glucosyl/glucuronosyl transferases | MET |  | 1.056 |
| AAEL001593 | glycerol-3-phosphate dehydrogenase | MET |  | -0.756 |
| AAEL002304 | porphobilinogen synthase | MET |  | -0.816 |
| AAEL002422 | cytoplasmic polyadenylation element binding protein (cpeb) | MET | -1.192 | -0.861 |
| AAEL002964 | brain chitinase and chia | MET |  | 1.046 |
| AAEL004059 | cystathionine beta-lyase | MET |  | 0.769 |
| AAEL004126 | sterol desaturase | MET |  | 1.252 |
| AAEL004127 | acyl-coa dehydrogenase | MET |  | 1 |
| AAEL004313 | fk506-binding protein | MET |  | 1.016 |
| AAEL004739 | acyl-coa dehydrogenase | MET |  | 1.391 |
| AAEL004757 | cleavage and polyadenylation specificity factor | MET |  | 0.768 |
| AAEL005732 | acyl-coa dehydrogenase | MET |  | 1.331 |
| AAEL005740 | AMP dependent ligase | MET |  | 0.94 |
| AAEL006085 | methylenetetrahydrofolate dehydrogenase | MET |  | 0.758 |
| AAEL006171 | n-myc downstream regulated | MET |  | 0.786 |
| AAEL006354 | epoxide hydrolase | MET |  | -0.851 |
| AAEL007097 | 4-nitrophenylphosphatase | MET | -1.064 |  |
| AAEL007201 | glutamyl aminopeptidase | MET |  | -0.888 |
| AAEL007707 | malate dehydrogenase | MET |  | -1.012 |
| AAEL007880 | ornithine decarboxylase | MET |  | -0.782 |
| AAEL007883 | fk506-binding protein | MET | 0.798 |  |
| AAEL008006 | 3-hydroxyacyl-coa dehyrogenase | MET |  | -1.513 |
| AAEL008144 | AMP dependent ligase | MET | 0.918 |  |
| AAEL008302 | glutamine-dependent nad(+) synthetase | MET |  | -0.961 |
| AAEL008330 | hexaprenyldihydroxybenzoate methyltransferase | MET |  | 1.062 |
| AAEL008467 | cysteine synthase | MET | 1.265 |  |
| AAEL009038 | prolylcarboxypeptidase, putative | MET | -1.184 |  |
| AAEL009246 | glycoside hydrolases | MET | 0.878 | 1.662 |
| AAEL009462 | hydroxyacylglutathione hydrolase | MET | 1.342 |  |
| AAEL009503 | 4-nitrophenylphosphatase | MET |  | 1.389 |
| AAEL009911 | rotamase | MET |  | 1.465 |
| AAEL010366 | glucosyl/glucuronosyl transferases | MET |  | 0.918 |
| AAEL010590 | aldose-1-epimerase | MET |  | -1.259 |
| AAEL010691 | ribonucleoside-diphosphate reductase small chain | MET |  | -0.945 |
| AAEL010938 | l-asparaginase | MET |  | 0.842 |
| AAEL011126 | alcohol dehydrogenase | MET |  | -0.794 |
| AAEL011130 | alcohol dehydrogenase | MET |  | -0.852 |
| AAEL011624 | granzyme A precursor, putative | MET | 0.894 |  |
| AAEL012179 | methylthioadenosine phosphorylase | MET | 0.834 |  |
| AAEL012312 | proliferation-associated 2g4 (pa2g4/ebp1) | MET |  | 0.841 |
| AAEL012341 | lysosomal acid lipase, putative | MET |  | -1.067 |
| AAEL012430 | AMP dependent ligase | MET |  | -1.048 |
| AAEL012697 | sterol carrier protein-2, putative | MET |  | -3.624 |
| AAEL012825 | bifunctional purine biosynthesis protein | MET | -1.089 |  |
| AAEL013245 | proacrosin, putative | MET |  | 1.485 |
| AAEL013458 | glutamine synthetase 1, 2 (glutamate-amonia ligase) (gs) | MET |  | -2.122 |
| AAEL013521 | tryptophanyl-tRNA synthetase | MET | 0.771 |  |
| AAEL013967 | Methylmalonyl-CoA carboxyltransferase 12S subunit, putative | MET | -1.655 |  |
| AAEL014662 | AMP dependent coa ligase | MET |  | 1.081 |
| AAEL014709 | methionine-tRNA synthetase | MET | -1.072 | 2.089 |
| AAEL015143 | glycine rich RNA binding protein, putative | MET | -1.01 |  |
| AAEL015337 | neutral alpha-glucosidase ab precursor (glucosidase ii alpha subunit) (alpha glucosidase 2) | MET | -0.967 | -2.407 |
| AAEL017039 | Conserved hypothetical protein (alcohol dehydrogenase 2 [Culex quinquefasciatus] _ | MET |  | 1.013 |
| AAEL017299 | AMP dependent coa ligase, putative | MET |  | 0.973 |
| AAEL000252 | hypothetical protein | PROT | 0.845 | 0.766 |
| AAEL005638 | conserved hypothetical protein | PROT |  | 1.034 |
| AAEL006323 | hypothetical protein | PROT | 0.797 | -3.277 |
| AAEL006542 | retinoid-inducible serine carboxypeptidase (serine carboxypeptidase | PROT | 0.932 | 1.054 |
| AAEL006563 | retinoid-inducible serine carboxypeptidase (serine carboxypeptidase | PROT |  | 1.62 |
| AAEL008862 | conserved hypothetical protein | PROT |  | -1.014 |
| AAEL009406 | n(4)-(beta-n-acetylglucosaminyl)-l-asparaginase | PROT | 1.252 |  |
| AAEL009771 | hypothetical protein | PROT |  | 0.816 |
| AAEL010196 | trypsin | PROT | 2.031 | 2.224 |
| AAEL011658 | plasma glutamate carboxypeptidase | PROT | 1.067 |  |
| AAEL014350 | hypothetical protein | PROT |  | -0.83 |
| AAEL014353 | conserved hypothetical protein | PROT |  | -0.957 |
| AAEL015432 | Trypsin, putative | PROT | -1.16 |  |
| AAEL015527 | conserved hypothetical protein | PROT |  | 0.768 |
| AAEL017451 | proteolysis, metallopeptidase activity, peptidyl-dipeptidase activity, membrane. | PROT |  | 0.789 |
| AAEL010592 | esterase, putative | RSM | 1.749 |  |
| AAEL010634 | hypothetical protein | RSM |  | 0.938 |
| AAEL000546 | carboxylesterase | RSM |  | -1.14 |
| AAEL000986 | NADH-ubiquinone oxidoreductase ashi subunit | RSM | 2.027 | 2.067 |
| AAEL001210 | NADH ubiquinone oxidoreductase subunit, putative | RSM | 1.049 |  |
| AAEL001960 | cytochrome P450 | RSM |  | -1.033 |
| AAEL002046 | cytochrome P450 | RSM |  | -0.82 |
| AAEL002683 | aldehyde oxidase | RSM | 1.058 |  |
| AAEL002886 | thioredoxin reductase | RSM |  | -0.8 |
| AAEL003380 | cytochrome P450 | RSM |  | 1.003 |
| AAEL003423 | NADH dehydrogenase, putative | RSM | 1.189 | -1.074 |
| AAEL003890 | cytochrome P450 | RSM | -1.064 |  |
| AAEL004450 | cytochrome b5, putative | RSM | 0.84 | 0.826 |
| AAEL004643 | mitochondrial ribosomal protein L1 | RSM |  | -0.805 |
| AAEL005178 | juvenile hormone esterase | RSM |  | 1.172 |
| AAEL005305 | conserved hypothetical protein | RSM |  | -0.824 |
| AAEL005946 | NADH-ubiquinone oxidoreductase subunit B14.5b | RSM |  | 0.956 |
| AAEL006230 | gonadotropin inducible transcription factor | RSM |  | -0.758 |
| AAEL006824 | cytochrome P450 | RSM |  | 2.468 |
| AAEL007046 | mitochondrial brown fat uncoupling protein | RSM |  | -0.854 |
| AAEL007355 | mitochondrial ribosomal protein, S18A, putative | RSM |  | 0.95 |
| AAEL007752 | cytochrome c oxidase, subunit VIIA, putative | RSM | 1.135 |  |
| AAEL007946 | glutathione-s-transferase theta, gst | RSM | 0.919 | -0.797 |
| AAEL008128 | mitochondrial inner membrane protein translocase, 13kD-subunit, putative | RSM |  | 0.781 |
| AAEL008397 | glutathione peroxidase | RSM |  | -1.617 |
| AAEL008757 | juvenile hormone esterase | RSM | 0.861 |  |
| AAEL009225 | mitochondrial ribosome recycling factor | RSM | -0.807 |  |
| AAEL010075 | oxidoreductase | RSM |  | 1.014 |
| AAEL010181 | mitochondrial ribosomal protein, L51, putative | RSM | 1.064 | 0.868 |
| AAEL011016 | carboxypeptidase m | RSM |  | 1.25 |
| AAEL012427 | metabolic proces, oxidoreductase activity, oxidation reduction | RSM |  | -0.863 |
| AAEL012845 | mitochondrial import inner membrane translocase subunit tim44 | RSM | 0.777 |  |
| AAEL013066 | checkpoint kinase | RSM |  | -1.071 |
| AAEL013555 | cytochrome P450 | RSM |  | 1.358 |
| AAEL013744 | NADH:ubiquinone dehydrogenase, putative | RSM | -1.282 |  |
| AAEL014019 | cytochrome P450 | RSM |  | 0.918 |
| AAEL014673 | NADH:ubiquinone dehydrogenase, putative | RSM |  | 1.103 |
| AAEL014830 | cytochrome P450 | RSM |  | 1.882 |
| AAEL014893 | cytochrome P450 | RSM | -1.374 |  |
| AAEL015578 | alpha-esterase | RSM |  | -1.038 |
| AAEL015635 | mitochondrial ribosomal protein, S10, putative | RSM | 1.038 |  |
| AAEL017071 | Alpha-esterase, putative | RSM |  | -1.378 |
| AAEL000032 | ribosomal protein S6 | RTT |  | 0.965 |
| AAEL000497 | histone h2a | RTT |  | -1.106 |
| AAEL000518 | histone h2a | RTT | 0.826 | -1.071 |
| AAEL000525 | histone h2a | RTT | 0.768 | -0.969 |
| AAEL002103 | histone H1, putative | RTT |  | -0.901 |
| AAEL002534 | 60S ribosomal protein L10 | RTT | -2.644 |  |
| AAEL002879 | heterogeneous nuclear ribonucleoprotein r | RTT |  | 0.895 |
| AAEL003071 | tRNA pseudouridine synthase D | RTT | 0.986 |  |
| AAEL003352 | ribosomal protein l7ae | RTT |  | 0.751 |
| AAEL003396 | 60S ribosomal protein L32 | RTT | -0.765 |  |
| AAEL003427 | ribosomal protein S9, putative | RTT |  | -0.751 |
| AAEL003646 | conserved hypothetical protein | RTT |  | 0.867 |
| AAEL003659 | histone H3 | RTT | 1.049 | -1.216 |
| AAEL003685 | histone H3 | RTT |  | -1.192 |
| AAEL003818 | histone h2a | RTT |  | -1.202 |
| AAEL003820 | histone h2a | RTT |  | -1.126 |
| AAEL003826 | histone h2a | RTT |  | -1.197 |
| AAEL003851 | histone h2a | RTT |  | -0.976 |
| AAEL003942 | 60S ribosomal protein L44 L41, putative | RTT |  | 1.133 |
| AAEL004978 | DEAD box ATP-dependent RNA helicase | RTT |  | -0.79 |
| AAEL005127 | ribonuclease UK114, putative | RTT | 0.872 |  |
| AAEL005129 | 40S ribosomal protein S30 | RTT | 0.859 | 2.634 |
| AAEL005368 | transcription initiation factor TFIIB | RTT |  | -0.92 |
| AAEL006698 | 60S ribosomal protein L31 | RTT | 0.812 |  |
| AAEL007005 | histone h2a | RTT | 0.795 |  |
| AAEL007078 | eukaryotic translation initiation factor 3, theta subunit | RTT |  | 1.249 |
| AAEL007928 | eukaryotic translation initiation factor 4 gamma | RTT |  | -0.827 |
| AAEL008266 | hypothetical protein | RTT |  | -0.847 |
| AAEL008500 | DEAD box ATP-dependent RNA helicase | RTT |  | -0.956 |
| AAEL009653 | 40S ribosomal protein S30 | RTT |  | 2.336 |
| AAEL010085 | DNA polymerase epsilon subunit, putative | RTT |  | -1.042 |
| AAEL010787 | DEAD box ATP-dependent RNA helicase | RTT |  | 1.474 |
| AAEL010821 | 60S acidic ribosomal protein P0 | RTT | 1.649 |  |
| AAEL011150 | RNA-binding protein precursor, putative | RTT |  | -0.775 |
| AAEL011251 | RNA binding motif protein | RTT | -0.934 |  |
| AAEL011447 | 60S ribosomal protein L14 | RTT | 1.526 | 1.235 |
| AAEL012074 | conserved hypothetical protein | RTT | 2.387 |  |
| AAEL012185 | ribosome biogenesis regulatory protein | RTT |  | 1.255 |
| AAEL012684 | conserved hypothetical protein | RTT |  | 1.378 |
| AAEL012686 | ribosomal protein S12, putative | RTT | 1.388 | 1.085 |
| AAEL012877 | homeobox protein extradenticle, putative | RTT |  | 0.978 |
| AAEL013221 | 60S ribosomal protein L10a | RTT |  | 1.023 |
| AAEL013964 | ribosomal protein L20, putative | RTT | 1.411 |  |
| AAEL014106 | ATP-dependent RNA helicase | RTT | -0.763 |  |
| AAEL014764 | acidic ribosomal protein P1, putative | RTT |  | 0.929 |
| AAEL014838 | 60S ribosomal protein L27e | RTT |  | -0.751 |
| AAEL015244 | splicing factor 3a | RTT | 1.212 |  |
| AAEL017595 | 5S ribosomal RNA [Source: RFAM 9.0] | RTT |  | 0.76 |
| AAEL017630 | 5S ribosomal RNA [Source: RFAM 9.0] | RTT |  | 0.782 |
| AAEL017685 | Nuclear RNase P [Source: RFAM 9.0] | RTT | 1.164 |  |
| AAEL017742 | 5S ribosomal RNA [Source: RFAM 9.0] | RTT | 0.846 | 0.766 |
| AAEL017760 | 5S ribosomal RNA [Source: RFAM 9.0] | RTT | 0.888 | 0.76 |
| AAEL017779 | 5S ribosomal RNA [Source: RFAM 9.0] | RTT |  | 0.757 |
| AAEL000471 | monocarboxylate transporter | TRP |  | 0.809 |
| AAEL001308 | CRAL/TRIO domain-containing protein | TRP |  | 1.065 |
| AAEL002063 | cationic amino acid transporter | TRP |  | 0.984 |
| AAEL002555 | sodium/solute symporter | TRP | 0.771 |  |
| AAEL002576 | sodium/solute symporter | TRP |  | 0.931 |
| AAEL002726 | D7 protein, putative | TRP | -0.869 |  |
| AAEL003548 | sulfate transporter | TRP |  | -0.859 |
| AAEL003626 | sodium/shloride dependent amino acid transporter | TRP |  | 0.832 |
| AAEL004247 | Sialin, Sodium/sialic acid cotransporter, putative | TRP |  | -0.758 |
| AAEL004855 | adp,atp carrier protein | TRP | -0.778 |  |
| AAEL005496 | zinc/iron transporter | TRP | 0.884 |  |
| AAEL005769 | glucose dehydrogenase | TRP |  | -0.836 |
| AAEL006138 | hypothetical protein | TRP |  | 1.353 |
| AAEL007458 | amino acid transporter | TRP | -0.833 | -0.945 |
| AAEL008406 | cationic amino acid transporter | TRP |  | 0.955 |
| AAEL008635 | abc transporter | TRP |  | 0.907 |
| AAEL009832 | exocyst complex protein exo70 | TRP |  | -1.481 |
| AAEL009863 | sodium/dicarboxylate cotransporter, putative | TRP | 1.445 | 1.056 |
| AAEL010102 | tetraspanin, putative | TRP |  | 1.164 |
| AAEL010434 | conserved hypothetical protein | TRP |  | 1.519 |
| AAEL010481 | sugar transporter | TRP |  | -1.701 |
| AAEL010485 | sugar transporter | TRP |  | 1 |
| AAEL010584 | vesicular mannose-binding lectin | TRP |  | -0.972 |
| AAEL011025 | vacuolar ATP synthase subunit ac39 | TRP |  | -2.707 |
| AAEL011244 | surfeit locus protein | TRP |  | 1.09 |
| AAEL012674 | d-amino acid oxidase | TRP | 1.387 |  |
| AAEL014927 | sodium/chloride dependent transporter | TRP |  | -1.124 |
| AAEL015549 | calcineurin b subunit | TRP |  | 0.769 |
| AAEL000019 | conserved hypothetical protein | UKN | 1.278 |  |
| AAEL000309 | hypothetical protein | UKN |  | -1.223 |
| AAEL000566 | conserved hypothetical protein | UKN | 0.946 | 1.748 |
| AAEL000619 | conserved hypothetical protein | UKN |  | 1.291 |
| AAEL001032 | conserved hypothetical protein | UKN | -0.753 |  |
| AAEL001107 | hypothetical protein | UKN | -0.837 | -0.924 |
| AAEL001323 | conserved hypothetical protein | UKN | 0.933 |  |
| AAEL001325 | conserved hypothetical protein | UKN |  | -1.639 |
| AAEL001414 | conserved hypothetical protein | UKN |  | 0.884 |
| AAEL001511 | conserved hypothetical protein | UKN | -0.769 |  |
| AAEL001718 | conserved hypothetical protein | UKN |  | 0.776 |
| AAEL001880 | conserved hypothetical protein | UKN |  | 1.242 |
| AAEL001885 | conserved hypothetical protein | UKN |  | 1.258 |
| AAEL001888 | hypothetical protein | UKN |  | 1.118 |
| AAEL001892 | conserved hypothetical protein | UKN |  | -1.661 |
| AAEL001897 | conserved hypothetical protein | UKN |  | -1.378 |
| AAEL002652 | hypothetical protein | UKN | -1.234 | -1.081 |
| AAEL002719 | conserved hypothetical protein | UKN |  | 0.81 |
| AAEL002758 | conserved hypothetical protein | UKN |  | 1.46 |
| AAEL002815 | conserved hypothetical protein | UKN |  | 1.197 |
| AAEL002828 | hypothetical protein | UKN |  | -0.805 |
| AAEL002889 | hypothetical protein | UKN | 2.243 |  |
| AAEL002900 | conserved hypothetical protein | UKN |  | 0.774 |
| AAEL003029 | hypothetical protein | UKN |  | 0.81 |
| AAEL003067 | conserved hypothetical protein | UKN |  | 0.997 |
| AAEL003482 | hypothetical protein | UKN | 1.257 | 1.157 |
| AAEL003766 | hypothetical protein | UKN |  | 0.901 |
| AAEL003842 | hypothetical protein | UKN |  | 0.838 |
| AAEL003944 | conserved hypothetical protein | UKN |  | 1.086 |
| AAEL004100 | hypothetical protein | UKN | 1.055 |  |
| AAEL004498 | hypothetical protein | UKN |  | 0.806 |
| AAEL004591 | hypothetical protein | UKN | 1.108 |  |
| AAEL004670 | conserved hypothetical protein | UKN |  | 1.172 |
| AAEL004809 | conserved hypothetical protein | UKN |  | 0.858 |
| AAEL004826 | conserved hypothetical protein | UKN |  | -1.156 |
| AAEL005106 | conserved hypothetical protein | UKN | 1.749 | 0.958 |
| AAEL005215 | conserved hypothetical protein | UKN |  | -0.759 |
| AAEL005620 | conserved hypothetical protein | UKN |  | 1.81 |
| AAEL005755 | hypothetical protein | UKN |  | 1.331 |
| AAEL005968 | conserved hypothetical protein | UKN | 1.69 |  |
| AAEL006131 | hypothetical protein | UKN | 0.861 |  |
| AAEL006585 | predicted protein | UKN | -0.898 |  |
| AAEL006629 | conserved hypothetical protein | UKN | -0.79 |  |
| AAEL006676 | conserved hypothetical protein | UKN | 0.891 |  |
| AAEL006792 | conserved hypothetical protein | UKN |  | -0.865 |
| AAEL006848 | conserved hypothetical protein | UKN |  | -0.876 |
| AAEL006863 | hypothetical protein | UKN |  | 3.042 |
| AAEL006969 | conserved hypothetical protein | UKN | 1.265 |  |
| AAEL006971 | conserved hypothetical protein | UKN |  | 0.876 |
| AAEL007259 | conserved hypothetical protein | UKN |  | -0.914 |
| AAEL007342 | conserved hypothetical protein | UKN |  | -0.814 |
| AAEL007703 | conserved hypothetical protein | UKN | 3.215 | 3.352 |
| AAEL007847 | conserved hypothetical protein | UKN |  | 1.119 |
| AAEL008025 | conserved hypothetical protein | UKN |  | 1.749 |
| AAEL008039 | conserved hypothetical protein | UKN |  | -1.212 |
| AAEL008100 | conserved hypothetical protein | UKN |  | -0.992 |
| AAEL008182 | conserved hypothetical protein | UKN | -1.003 |  |
| AAEL008274 | conserved hypothetical protein | UKN |  | -1.427 |
| AAEL008286 | conserved hypothetical protein | UKN |  | -1.18 |
| AAEL008365 | conserved hypothetical protein | UKN |  | -1.158 |
| AAEL008485 | conserved hypothetical protein | UKN |  | -1.848 |
| AAEL008492 | conserved hypothetical protein | UKN | 1.721 | 1.275 |
| AAEL008729 | hypothetical protein | UKN | -0.788 | -0.835 |
| AAEL008771 | conserved hypothetical protein | UKN |  | 1.584 |
| AAEL008802 | conserved hypothetical protein | UKN |  | 0.829 |
| AAEL009177 | conserved hypothetical protein | UKN |  | -0.864 |
| AAEL009201 | conserved hypothetical protein | UKN | 0.754 | 1.139 |
| AAEL009487 | hypothetical protein | UKN |  | 0.893 |
| AAEL009519 | hypothetical protein | UKN |  | -0.785 |
| AAEL010752 | hypothetical protein | UKN |  | -1.077 |
| AAEL011010 | conserved hypothetical protein | UKN |  | 1.191 |
| AAEL011330 | conserved hypothetical protein | UKN |  | -0.807 |
| AAEL011388 | conserved hypothetical protein | UKN |  | 0.756 |
| AAEL011456 | conserved hypothetical protein | UKN |  | 1.762 |
| AAEL011532 | hypothetical protein | UKN |  | -0.827 |
| AAEL011665 | hypothetical protein | UKN |  | -0.762 |
| AAEL011884 | hypothetical protein | UKN |  | -2.065 |
| AAEL011928 | conserved hypothetical protein | UKN | 1.037 | 0.87 |
| AAEL012208 | hypothetical protein | UKN |  | -1.004 |
| AAEL012293 | conserved hypothetical protein | UKN | 1.193 |  |
| AAEL012454 | conserved hypothetical protein | UKN |  | 1.13 |
| AAEL012710 | conserved hypothetical protein | UKN | 0.905 | 0.831 |
| AAEL012858 | hypothetical protein | UKN |  | -0.952 |
| AAEL012859 | conserved hypothetical protein | UKN | -0.75 |  |
| AAEL012860 | conserved hypothetical protein | UKN |  | 0.912 |
| AAEL012862 | hypothetical protein | UKN | 1.151 | 2.434 |
| AAEL012867 | conserved hypothetical protein | UKN |  | 1.242 |
| AAEL013287 | conserved hypothetical protein (cystatin-like domain; cysteine-type endopeptidase inhibitor activity) | UKN | -1.37 |  |
| AAEL013300 | conserved hypothetical protein | UKN |  | -0.786 |
| AAEL013484 | hypothetical protein | UKN |  | -0.754 |
| AAEL013486 | hypothetical protein | UKN |  | -1.061 |
| AAEL013734 | hypothetical protein | UKN | -1.287 | -0.987 |
| AAEL013800 | conserved hypothetical protein | UKN |  | -0.864 |
| AAEL013843 | conserved hypothetical protein | UKN | 1.543 |  |
| AAEL014068 | conserved hypothetical protein | UKN | 1.351 | 0.863 |
| AAEL014171 | conserved hypothetical protein | UKN |  | -1.184 |
| AAEL014300 | hypothetical protein | UKN | 0.842 | 0.868 |
| AAEL014388 | conserved hypothetical protein | UKN |  | -0.913 |
| AAEL014511 | predicted protein | UKN |  | -0.883 |
| AAEL014565 | hypothetical protein | UKN |  | -0.957 |
| AAEL014937 | hypothetical protein | UKN | -0.945 | -1.289 |
| AAEL015379 | conserved hypothetical protein | UKN | -0.888 |  |
| AAEL017016 | Conserved hypothetical protein | UKN | 0.828 | 0.958 |
| AAEL017034 | Hypothetical protein | UKN |  | -1.584 |
| AAEL017144 | Hypothetical protein | UKN | 1.364 |  |
| AAEL017190 | Hypothetical protein | UKN |  | -1.305 |
| AAEL017455 | hypothetical protein | UKN | -0.981 | 1.642 |
| AAEL017491 | hypothetical protein | UKN | -1.251 | -1.001 |
| AAEL017530 | hypothetical protein | UKN |  | -1.363 |
