# Supplementary material for: Engineered Aedes aegypti JAK/STAT Pathway-Mediated Immunity to Dengue Virus
Source: PLoS Negl Trop Dis. 2017 Jan 12;11(1):e0005187. doi: 10.1371/journal.pntd.0005187 (PMC5230736; doi:10.1371/journal.pntd.0005187)
Supplement: S2 Table — Statistical analyses were performed using a hypergeometric test with phyper in R. (DOCX) [file pntd.0005187.s007.docx]

**Table S2. Numerical data and hypergeometric statistics of over-representation analysis of gene functional category from fat body and midgut transcriptome of VgDome and VgHop lines.** Statistical analyses were performed using a hypergeometric test with phyper in R.

| **Fat body** | | | | | | | |
| --- | --- | --- | --- | --- | --- | --- | --- |
| **Functional category** | **VgDome enriched** | | | **VgHop enriched** | | | |
|  | **% of significantly regulated gene** | **Fold differences compared to transcriptome** | **p-value** | **% of significantly regulated gene** | **Fold differences compared to transcriptome** | **p-value** |  |
| **CS** | 0.28 | 0.38 | 0.9050 | 0.14 | 0.10 | 0.9998 |  |
| **CSR** | 0.30 | 0.40 | 0.7175 | 0.60 | 0.41 | 0.8703 |  |
| **DIV** | 0.57 | 0.76 | 0.9786 | 1.33 | 0.91 | 0.8579 |  |
| **DIG** | 1.55 | 2.07 | 0.0729 | 1.55 | 1.06 | 0.2929 |  |
| **IMM** | 2.58 | 3.44 | 0.0000 | 4.40 | 3.01 | 0.0000 |  |
| **MET** | 0.84 | 1.12 | 0.2870 | 2.88 | 1.97 | 0.0001 |  |
| **PROT** | 1.63 | 2.17 | 0.0212 | 2.17 | 1.48 | 0.0938 |  |
| **RSM** | 1.74 | 2.32 | 0.0020 | 2.32 | 1.58 | 0.0020 |  |
| **RTT** | 0.76 | 1.01 | 0.4099 | 0.94 | 0.64 | 0.9866 |  |
| **TRP** | 0.49 | 0.65 | 0.7416 | 1.71 | 1.17 | 0.2228 |  |
| **UKN** | 0.60 | 0.80 | 0.8747 | 1.15 | 0.79 | 0.9672 |  |
| **Transcriptome** | 0.75 | - | - | 1.46 | - | - |  |
| **Functional category** | **VgDome depleted** | | | **VgHop depleted** | | | |
|  | **% of significantly regulated gene** | **Fold differences compared to transcriptome** | **p-value** | **% of significantly regulated gene** | **Fold differences compared to transcriptome** | **p-value** |  |
| **CS** | 0.71 | 1.51 | 0.1136 | 1.41 | 1.20 | 0.2281 |  |
| **CSR** | 0.00 | 0.00 | 0.7937 | 0.00 | 0.00 | 0.9815 |  |
| **DIV** | 0.47 | 1.02 | 0.4088 | 1.09 | 0.93 | 0.7627 |  |
| **DIG** | 0.78 | 1.66 | 0.1219 | 1.55 | 1.32 | 0.1943 |  |
| **IMM** | 1.52 | 3.25 | 0.0002 | 2.28 | 1.94 | 0.0050 |  |
| **MET** | 0.74 | 1.59 | 0.0630 | 1.67 | 1.42 | 0.0502 |  |
| **PROT** | 0.27 | 0.58 | 0.5168 | 1.08 | 0.92 | 0.4383 |  |
| **RSM** | 0.58 | 1.24 | 0.2201 | 2.17 | 1.85 | 0.0076 |  |
| **RTT** | 0.18 | 0.38 | 0.9839 | 0.85 | 0.72 | 0.9275 |  |
| **TRP** | 0.37 | 0.78 | 0.5369 | 1.10 | 0.93 | 0.4993 |  |
| **UKN** | 0.38 | 0.80 | 0.7970 | 1.08 | 0.91 | 0.7186 |  |
| **Transcriptome** | 0.47 | - | - | 1.18 | - | - |  |
|  |  |  |  |  |  |  |  |
| **Midgut** | | | | | | | |
| **Functional category** | **VgDome enriched** | | | **VgHop enriched** | | | |
|  | **% of significantly regulated gene** | **Fold differences compared to transcriptome** | **p-value** | **% of significantly regulated gene** | **Fold differences compared to transcriptome** | **p-value** |  |
| **CS** | 2.40 | 1.00 | 0.4322 | 1.27 | 0.60 | 0.9406 |  |
| **CSR** | 0.60 | 0.25 | 0.9875 | 1.50 | 0.71 | 0.7081 |  |
| **DIV** | 2.54 | 1.06 | 0.1480 | 2.62 | 1.25 | 0.0001 |  |
| **DIG** | 11.63 | 4.86 | 0.0000 | 7.75 | 3.68 | 0.0001 |  |
| **IMM** | 2.28 | 0.95 | 0.5123 | 2.12 | 1.01 | 0.4150 |  |
| **MET** | 4.37 | 1.83 | 0.0000 | 3.62 | 1.72 | 0.0003 |  |
| **PROT** | 2.17 | 0.91 | 0.5240 | 4.34 | 2.06 | 0.0022 |  |
| **RSM** | 4.35 | 1.82 | 0.0006 | 2.90 | 1.38 | 0.0586 |  |
| **RTT** | 1.07 | 0.45 | 1.0000 | 0.81 | 0.38 | 1.0000 |  |
| **TRP** | 4.02 | 1.68 | 0.0013 | 0.98 | 0.46 | 0.9910 |  |
| **UKN** | 1.58 | 0.66 | 1.0000 | 1.50 | 0.71 | 0.9989 |  |
| **Transcriptome** | 2.39 | - | - | 2.10 | - | - |  |
| **Functional category** | **VgDome depleted** | | | **VgHop depleted** | | | |
|  | **% of significantly regulated gene** | **Fold differences compared to transcriptome** | **p-value** | **% of significantly regulated gene** | **Fold differences compared to transcriptome** | **p-value** |  |
| **CS** | 0.85 | 0.49 | 0.9636 | 0.85 | 0.49 | 0.9674 |  |
| **CSR** | 0.60 | 0.35 | 0.9299 | 0.60 | 0.35 | 0.9299 |  |
| **DIV** | 1.58 | 0.92 | 0.8504 | 1.56 | 0.91 | 0.8771 |  |
| **DIG** | 8.53 | 4.95 | 0.0000 | 6.20 | 3.60 | 0.0004 |  |
| **IMM** | 1.82 | 1.06 | 0.3491 | 3.64 | 2.11 | 0.0002 |  |
| **MET** | 3.81 | 2.21 | 0.0000 | 3.53 | 2.05 | 0.0000 |  |
| **PROT** | 4.88 | 2.83 | 0.0000 | 2.17 | 1.26 | 0.1884 |  |
| **RSM** | 2.90 | 1.68 | 0.0086 | 3.04 | 1.77 | 0.0043 |  |
| **RTT** | 0.89 | 0.52 | 0.9996 | 0.72 | 0.42 | 1.0000 |  |
| **TRP** | 2.07 | 1.20 | 0.1755 | 1.71 | 0.99 | 0.4442 |  |
| **UKN** | 1.30 | 0.75 | 0.9901 | 1.58 | 0.91 | 0.7718 |  |
| **Transcriptome** | 1.72 | - | - | 1.72 | - | - |  |
