# Supplementary material for: Engineered Aedes aegypti JAK/STAT Pathway-Mediated Immunity to Dengue Virus
Source: PLoS Negl Trop Dis. 2017 Jan 12;11(1):e0005187. doi: 10.1371/journal.pntd.0005187 (PMC5230736; doi:10.1371/journal.pntd.0005187)
Supplement: S3 Table — Functional group abbreviations: CS, cytoskeletal and structural; CSR, chemosensory reception; DIV, diverse functions; DIG, blood and sugar food digestive; IMM, immunity; MET, metabolism; PROT, proteolysis; RSM, redox, stress and mitochondrion; RTT, replication, transcription, and translation; TRP, transport; UKN, unknown functions. (DOCX) [file pntd.0005187.s008.docx]

**Table S3. Log_2_-fold values and functional groups of transcripts that are significantly enriched or depleted in the midgut of VgDome or VgHop mosquitoes relative to WT mosquitoes.** Functional group abbreviations: CS, cytoskeletal and structural; CSR, chemosensory reception; DIV, diverse functions; DIG, blood and sugar food digestive; IMM, immunity; MET, metabolism; PROT, proteolysis; RSM, redox, stress and mitochondrion; RTT, replication, transcription, and translation; TRP, transport; UKN, unknown functions.

| **Gene ID** | **Description** | **Functional group** | **VgDome MG** | **VgHop MG** |
| --- | --- | --- | --- | --- |
| AAEL000840 | skeletal muscle/kidney enriched inositol 5-phosphatase | CS | 1.035 |  |
| AAEL001829 | beta nu integrin subunit | CS | 0.787 |  |
| AAEL001951 | actin | CS | 1.19 |  |
| AAEL002185 | cuticle protein, putative | CS | 1.451 | 1.731 |
| AAEL002623 | conserved hypothetical protein | CS | 0.791 |  |
| AAEL002630 | conserved hypothetical protein | CS | 0.836 |  |
| AAEL003024 | netrin | CS | -0.991 |  |
| AAEL004758 | pupal cuticle protein, putative | CS | 1.518 | 1.167 |
| AAEL004798 | conserved hypothetical protein (mucin-like protein) | CS | 1.901 |  |
| AAEL005146 | conserved hypothetical protein | CS | -1.486 |  |
| AAEL005407 | annexin x | CS |  | -1.195 |
| AAEL005408 | annexin x | CS |  | 1.579 |
| AAEL005412 | annexin x | CS | -0.758 |  |
| AAEL005961 | actin | CS |  | 0.778 |
| AAEL006042 | arginine or creatine kinase | CS |  | -0.817 |
| AAEL006911 | microtubule-associated protein | CS | 0.804 |  |
| AAEL006953 | conserved hypothetical protein | CS |  | -1.704 |
| AAEL007132 | dynein heavy chain | CS |  | -1.228 |
| AAEL007162 | gaba(a) receptor-associated protein | CS | -0.903 |  |
| AAEL007439 | myosin light chain 1, putative | CS | 1.021 |  |
| AAEL008315 | calponin/transgelin | CS |  | 0.814 |
| AAEL010289 | beta nu integrin subunit | CS | 0.827 |  |
| AAEL010307 | conserved hypothetical protein | CS | 1.32 |  |
| AAEL010984 | conserved hypothetical protein | CS | 1.181 |  |
| AAEL011336 | conserved hypothetical protein | CS |  | 1.076 |
| AAEL011803 | prohibitin, putative | CS |  | -2.016 |
| AAEL012631 | myosin i | CS |  | 1.906 |
| AAEL012644 | conserved hypothetical protein | CS | 3.341 |  |
| AAEL012888 | dcapl | CS | 0.808 |  |
| AAEL013515 | Pupal cuticle protein, putative | CS | -1.199 |  |
| AAEL013984 | structural constituent of cuticle | CS |  | -1.509 |
| AAEL014084 | kinesin eg-5 | CS | 1.343 |  |
| AAEL014668 | ankyrin 2,3/unc44 | CS |  | 2.698 |
| AAEL014742 | ankyrin 2,3/unc44 | CS | -0.908 | 2.533 |
| AAEL017334 | Conserved hypothetical protein (chitin-binding domain type 2) | CS | 3.197 |  |
| AAEL001705 | odorant response protein ODR-4, putative | CSR |  | 0.821 |
| AAEL002587 | Odorant-binding protein 56e, putative | CSR |  | 0.885 |
| AAEL002606 | Odorant-binding protein 56e, putative | CSR | 1.632 | 2.99 |
| AAEL005770 | Odorant-binding protein 99c, putative | CSR | 0.834 |  |
| AAEL005772 | Odorant-binding protein 99c, putative | CSR | -1.324 | -0.877 |
| AAEL006108 | conserved hypothetical protein | CSR |  | 0.759 |
| AAEL006109 | conserved hypothetical protein | CSR |  | -0.797 |
| AAEL010666 | Odorant-binding protein 58c, putative | CSR |  | 1.23 |
| AAEL017316 | odorant receptor [Source:Aedes_ManualAnnotation;Acc:AAEL800100] | CSR | -0.849 |  |
| AAEL000067 | hypothetical protein | DIV | 0.764 |  |
| AAEL000088 | brefeldin A-sensitive peripheral Golgi protein, putative | DIV | 0.911 |  |
| AAEL000102 | conserved hypothetical protein | DIV |  | 0.829 |
| AAEL000105 | beta-alanine synthase, putative | DIV | -1.423 | -1.497 |
| AAEL000126 | molybdopterin cofactor synthesis protein a | DIV | 1.22 | 0.875 |
| AAEL000241 | conserved hypothetical protein | DIV | 0.803 |  |
| AAEL000315 | pigeon protein (linotte protein) | DIV | 0.795 |  |
| AAEL000385 | developmentally regulated RNA-binding protein | DIV | -1.486 | -0.882 |
| AAEL000444 | UDP-glucose glycoprotein:glucosyltransferase | DIV |  | 1.748 |
| AAEL000544 | hypothetical protein | DIV |  | 0.833 |
| AAEL000551 | hypothetical protein (pacifastin light chain [Culex quinquefasciatus]) | DIV |  | -2.653 |
| AAEL000673 | conserved hypothetical protein | DIV | 0.959 |  |
| AAEL000718 | virus-induced RNA, putative | DIV |  | 1.029 |
| AAEL000719 | pak-interacting exchange factor, beta-pix/cool-1 | DIV |  | 0.863 |
| AAEL000757 | anterior fat body protein | DIV | -1.94 | -0.867 |
| AAEL000813 | dimethylaniline monooxygenase | DIV | 0.85 | 0.779 |
| AAEL000845 | protein-tyrosine phosphatase | DIV | 1.378 | 0.953 |
| AAEL000910 | conserved hypothetical protein | DIV | 1.505 | 1.493 |
| AAEL000931 | alkaline phosphatase | DIV | 1.23 |  |
| AAEL000973 | conserved hypothetical protein | DIV |  | -0.791 |
| AAEL001007 | conserved hypothetical protein | DIV | -1.138 | -1.094 |
| AAEL001017 | tyrosine-protein kinase | DIV | -0.978 |  |
| AAEL001022 | anterior fat body protein | DIV |  | -1.586 |
| AAEL001023 | p15-2b protein, putative | DIV |  | -0.759 |
| AAEL001087 | synaptic vesicle protein | DIV |  | -0.8 |
| AAEL001100 | phosphoserine phosphatase | DIV |  | 1.405 |
| AAEL001111 | zinc finger protein | DIV | 0.883 |  |
| AAEL001166 | protein phosphatases pp1 regulatory subunit | DIV | 0.836 |  |
| AAEL001191 | conserved hypothetical protein | DIV | 0.936 |  |
| AAEL001238 | bap28 | DIV |  | -0.754 |
| AAEL001287 | conserved hypothetical protein | DIV |  | 0.759 |
| AAEL001293 | conserved hypothetical protein | DIV | -0.892 |  |
| AAEL001301 | conserved hypothetical protein | DIV |  | -0.76 |
| AAEL001306 | conserved hypothetical protein | DIV |  | 0.922 |
| AAEL001352 | scaffold attachment factor b | DIV |  | -0.938 |
| AAEL001385 | conserved hypothetical protein | DIV | -0.838 |  |
| AAEL001401 | conserved hypothetical protein | DIV |  | -0.979 |
| AAEL001402 | conserved hypothetical protein | DIV |  | -1.392 |
| AAEL001420 | conserved hypothetical protein | DIV |  | -1.98 |
| AAEL001431 | hypothetical protein | DIV | 1.112 |  |
| AAEL001479 | protoheme ix farnesyltransferase | DIV |  | 0.936 |
| AAEL001627 | UDP-n-acteylglucosamine pyrophosphorylase | DIV |  | -1.006 |
| AAEL001667 | multicopper oxidase | DIV | -1.345 |  |
| AAEL001670 | conserved hypothetical protein | DIV | 0.807 |  |
| AAEL001682 | nuclear movement protein nudc | DIV | 0.822 |  |
| AAEL001698 | charged multivesicular body protein 4b | DIV |  | 2.302 |
| AAEL001714 | p15-2a protein, putative | DIV |  | -0.814 |
| AAEL001784 | cytidine and deoxycytidylate deaminase zinc-binding region | DIV | -0.965 |  |
| AAEL001900 | lactosylceramide 4-alpha-galactosyltransferase (alpha- 1,4-galactosyltransferase) | DIV |  | 1.287 |
| AAEL001954 | px serine/threonine kinase (pxk) | DIV |  | 0.814 |
| AAEL001965 | imaginal disc growth factor | DIV |  | -1.452 |
| AAEL002048 | histidyl-tRNA synthetase | DIV |  | 1.605 |
| AAEL002077 | uv excision repair protein rad23 | DIV | -0.825 | 1.316 |
| AAEL002081 | conserved hypothetical protein | DIV | -0.858 |  |
| AAEL002158 | secreted ferritin G subunit precursor, putative | DIV | -0.815 |  |
| AAEL002182 | t-diRNAhydrouridine synthase | DIV | -0.904 | -0.802 |
| AAEL002248 | conserved hypothetical protein | DIV | 0.808 |  |
| AAEL002257 | conserved hypothetical protein | DIV |  | -0.768 |
| AAEL002258 | conserved hypothetical protein | DIV | 1.05 | 0.815 |
| AAEL002267 | conserved hypothetical protein | DIV |  | 0.848 |
| AAEL002349 | conserved hypothetical protein | DIV | 0.773 |  |
| AAEL002387 | conserved hypothetical protein | DIV |  | 0.849 |
| AAEL002395 | cdk8 | DIV |  | 1.404 |
| AAEL002454 | conserved hypothetical protein | DIV | -0.897 |  |
| AAEL002492 | hypothetical protein | DIV |  | -0.934 |
| AAEL002501 | protein disulfide isomerase | DIV |  | 2.149 |
| AAEL002539 | fimbrin/plastin | DIV |  | 0.808 |
| AAEL002691 | conserved hypothetical protein | DIV | 0.786 |  |
| AAEL002701 | mannosyltransferase | DIV | 1.081 |  |
| AAEL002738 | synaptic vesicle protein | DIV | 1.968 |  |
| AAEL002748 | aspartyl-tRNA synthetase | DIV |  | 0.756 |
| AAEL002764 | dihydrolipoamide succinyltransferase component of 2-oxoglutarate dehydrogenase | DIV |  | 0.947 |
| AAEL002796 | l-asparaginase i | DIV |  | 0.866 |
| AAEL002808 | diphthine synthase | DIV | -0.873 |  |
| AAEL002834 | myo-inositol-1 phosphate synthase | DIV | 1.115 |  |
| AAEL002860 | conserved hypothetical protein | DIV | 2.987 |  |
| AAEL002919 | conserved hypothetical protein | DIV | 0.804 |  |
| AAEL002920 | hypothetical protein | DIV | 2.143 | -1.368 |
| AAEL002937 | hypothetical protein | DIV |  | -1.483 |
| AAEL002943 | che-11 | DIV |  | 1.242 |
| AAEL002948 | frataxin, putative | DIV |  | -1.007 |
| AAEL002950 | conserved hypothetical protein | DIV | -1.033 |  |
| AAEL002996 | dolichyl glycosyltransferase | DIV |  | 1.401 |
| AAEL002999 | gtpase_rho | DIV |  | 1.803 |
| AAEL003015 | protein phosphatase 2a, regulatory subunit | DIV |  | -1.468 |
| AAEL003022 | serine/threonine-protein kinase | DIV |  | -0.913 |
| AAEL003046 | saposin | DIV | -0.753 |  |
| AAEL003054 | conserved hypothetical protein | DIV | -1.136 |  |
| AAEL003109 | atlastin | DIV |  | 2.216 |
| AAEL003123 | deoxyribonuclease I, putative | DIV | 1.689 |  |
| AAEL003153 | hypothetical protein | DIV |  | 0.752 |
| AAEL003213 | guanine deaminase | DIV | 0.794 |  |
| AAEL003237 | low molecular weight protein-tyrosine-phosphatase | DIV | 1.979 |  |
| AAEL003286 | alkaline phosphatase | DIV | -1.114 |  |
| AAEL003287 | arsenitesistance protein | DIV | 1.43 |  |
| AAEL003317 | alkaline phosphatase | DIV | -0.813 |  |
| AAEL003326 | protein phosphatase 2c | DIV | 1.088 | 0.996 |
| AAEL003327 | zinc finger protein | DIV |  | 0.765 |
| AAEL003336 | conserved hypothetical protein | DIV | 2.263 |  |
| AAEL003338 | hypothetical protein | DIV |  | 0.827 |
| AAEL003345 | argininosuccinate lyase | DIV |  | -1.054 |
| AAEL003413 | f-spondin | DIV |  | -0.798 |
| AAEL003443 | threonine dehydrogenase | DIV | 0.859 |  |
| AAEL003448 | conserved hypothetical protein | DIV |  | 1.225 |
| AAEL003599 | DNA binding, sulfiredoxin activity, oxidation reduction | DIV | 1.075 |  |
| AAEL003606 | purine biosynthesis protein 6, pur6 | DIV | -1.17 |  |
| AAEL003732 | conserved hypothetical protein (ankyrin repeats) | DIV | 0.955 |  |
| AAEL003824 | conserved hypothetical protein | DIV | 0.799 |  |
| AAEL003883 | conserved hypothetical protein | DIV |  | -0.99 |
| AAEL003887 | vacuolar membrane protein pep11 | DIV |  | 1.318 |
| AAEL003912 | trafficking protein particle complex subunit 6b | DIV |  | -0.876 |
| AAEL003922 | conserved hypothetical protein | DIV |  | -1.388 |
| AAEL003954 | Juvenile hormone-inducible protein, putative | DIV |  | 0.76 |
| AAEL004092 | deoxyribonuclease I, putative | DIV |  | 1.167 |
| AAEL004101 | hypothetical protein | DIV | 0.861 |  |
| AAEL004107 | nucleoside diphosphate kinase, putative | DIV |  | -0.893 |
| AAEL004121 | ubiquitin-conjugating enzyme E2 q | DIV | 1.471 | 1.17 |
| AAEL004188 | conserved hypothetical protein | DIV | 0.876 |  |
| AAEL004237 | vacuolar protein sorting 18 (deep orange protein) | DIV |  | 1.014 |
| AAEL004260 | parkin (ubiquitin E3 ligase prkn) | DIV | -0.778 |  |
| AAEL004277 | conserved hypothetical protein | DIV |  | 1.959 |
| AAEL004278 | conserved hypothetical protein | DIV | -0.826 | 1.486 |
| AAEL004333 | pax neighbour protein | DIV | -1.071 |  |
| AAEL004335 | secreted ferritin G subunit precursor, putative | DIV | -1.573 | -1.277 |
| AAEL004392 | IAP-antagonist michelob-X-like protein, pro-apoptotic protein | DIV | 0.767 |  |
| AAEL004404 | HIG1 domain family member 2A, putative | DIV |  | 0.821 |
| AAEL004406 | conserved hypothetical protein | DIV | 1.879 |  |
| AAEL004409 | yellow protein, putative | DIV | 1.55 |  |
| AAEL004490 | conserved hypothetical protein | DIV |  | 0.85 |
| AAEL004513 | neurotransmitter gated ion channel | DIV | 0.933 |  |
| AAEL004568 | adpbosylation factor, arf | DIV |  | -1.523 |
| AAEL004575 | beta-galactosidase | DIV |  | 1.292 |
| AAEL004580 | beta-galactosidase | DIV |  | -0.788 |
| AAEL004582 | beta-galactosidase | DIV |  | -2.317 |
| AAEL004592 | tyrosine-protein kinase src64b | DIV |  | 1.058 |
| AAEL004665 | conserved hypothetical protein | DIV | -1.148 |  |
| AAEL004750 | nonmuscle myosin heavy chain-A, putative | DIV |  | 0.778 |
| AAEL004756 | vesicle docking protein P115 | DIV |  | 1.829 |
| AAEL004827 | conserved hypothetical protein | DIV | -0.833 |  |
| AAEL004835 | conserved hypothetical protein | DIV | 0.781 |  |
| AAEL004842 | conserved hypothetical protein | DIV | 0.757 |  |
| AAEL004845 | conserved hypothetical protein | DIV | -0.781 |  |
| AAEL004860 | acireductone dioxygenase | DIV | 2.322 |  |
| AAEL004916 | conserved hypothetical protein | DIV |  | 0.897 |
| AAEL004967 | myo inositol monophosphatase | DIV | 1.264 | 1.119 |
| AAEL004969 | conserved hypothetical protein | DIV |  | -1.284 |
| AAEL005037 | seryl-tRNA synthetase | DIV | 1.459 | 1.119 |
| AAEL005218 | conserved hypothetical protein | DIV | -0.817 |  |
| AAEL005255 | par domain protein | DIV | 0.918 |  |
| AAEL005270 | transferase activty | DIV | -0.783 |  |
| AAEL005308 | pyruvate dehydrogenase | DIV | 0.931 | 0.932 |
| AAEL005312 | conserved hypothetical protein | DIV | -1.207 |  |
| AAEL005349 | conserved hypothetical protein | DIV |  | 1.49 |
| AAEL005353 | conserved hypothetical protein | DIV |  | -1.442 |
| AAEL005384 | phosphoribosylformylglycinamidine synthase, putative | DIV | 2.08 | 1.246 |
| AAEL005402 | hypothetical protein | DIV |  | -0.782 |
| AAEL005425 | conserved hypothetical protein | DIV |  | 1.594 |
| AAEL005455 | ctp synthase | DIV |  | 2.706 |
| AAEL005458 | carnitine o-acyltransferase | DIV |  | 1.828 |
| AAEL005519 | synaptotagmin-14 | DIV | 1.061 |  |
| AAEL005523 | Selenoprotein G, putative | DIV | 1.2 |  |
| AAEL005536 | tetraspanin 29fb | DIV | 0.873 | -1.102 |
| AAEL005543 | conserved hypothetical protein | DIV | 1.285 |  |
| AAEL005557 | hypothetical protein | DIV | -0.88 |  |
| AAEL005572 | hypothetical protein | DIV |  | 0.835 |
| AAEL005651 | ethanolamine-phosphate cytidylyltransferase | DIV | 1.565 |  |
| AAEL005739 | conserved hypothetical protein | DIV |  | 0.934 |
| AAEL005743 | conserved hypothetical protein | DIV |  | 1.775 |
| AAEL005760 | hypothetical protein | DIV | -0.945 |  |
| AAEL005768 | hypothetical protein | DIV |  | 1.298 |
| AAEL005816 | chromatin regulatory protein sir2 | DIV |  | 1.073 |
| AAEL005819 | l-allo-threonine aldolase | DIV |  | -3.005 |
| AAEL005826 | conserved hypothetical protein | DIV | -1.04 |  |
| AAEL005839 | uridine phosphorylase | DIV | 1.703 |  |
| AAEL005849 | synaptic vesicle protein | DIV | 0.757 |  |
| AAEL005881 | autocrine motility factor receptor, amfr | DIV |  | 0.81 |
| AAEL005890 | conserved hypothetical protein | DIV | 0.83 |  |
| AAEL005947 | Gch sequence factor-1, putative | DIV | 1.51 |  |
| AAEL005959 | phospholipase b, plb1 | DIV | 1.194 | 1.397 |
| AAEL005976 | adenine phosphoribosyltransferase, putative | DIV | 0.949 |  |
| AAEL005997 | allergen, putative | DIV | 0.808 | -0.886 |
| AAEL006020 | hypothetical protein | DIV |  | -0.931 |
| AAEL006024 | Vanin-like protein 2 precursor, putative | DIV |  | 0.877 |
| AAEL006071 | hypothetical protein | DIV |  | -0.918 |
| AAEL006190 | conserved hypothetical protein | DIV | -0.891 |  |
| AAEL006203 | Juvenile hormone-inducible protein, putative | DIV | 0.781 |  |
| AAEL006207 | conserved hypothetical protein | DIV | -0.788 |  |
| AAEL006213 | tartan protein, putative | DIV | -1.026 |  |
| AAEL006279 | hypothetical protein | DIV |  | 1.316 |
| AAEL006309 | conserved hypothetical protein | DIV | 0.923 |  |
| AAEL006314 | upstream transcription factor | DIV |  | 0.806 |
| AAEL006315 | 26S proteasome regulatory subunit rpn1 | DIV |  | -1.139 |
| AAEL006321 | 1-acylglycerol-3-phosphate acyltransferase | DIV |  | -0.966 |
| AAEL006350 | conserved hypothetical protein | DIV |  | 0.757 |
| AAEL006446 | trehalose-6-phosphate synthase | DIV | -0.925 | -0.969 |
| AAEL006466 | chondroitin synthase | DIV |  | -0.766 |
| AAEL006480 | conserved hypothetical protein | DIV |  | -0.806 |
| AAEL006483 | conserved hypothetical protein | DIV | 1.983 | 1.057 |
| AAEL006507 | conserved hypothetical protein | DIV | 1.098 | 1.298 |
| AAEL006515 | conserved hypothetical protein | DIV | 1.073 | 0.865 |
| AAEL006544 | nucleoporin P54 | DIV |  | -0.861 |
| AAEL006553 | protein disulfide isomerase, putative | DIV |  | 0.901 |
| AAEL006557 | conserved hypothetical protein | DIV | 2.319 | 1.183 |
| AAEL006587 | conserved hypothetical protein | DIV |  | 0.894 |
| AAEL006600 | Juvenile hormone-inducible protein, putative | DIV | 0.906 |  |
| AAEL006602 | hypothetical protein | DIV | 1.85 | 1.425 |
| AAEL006625 | conserved hypothetical protein | DIV | -0.814 | 1.307 |
| AAEL006672 | conserved hypothetical protein | DIV | -1.219 |  |
| AAEL006733 | fad nadph dehydrogenase | DIV | -0.832 |  |
| AAEL006859 | Myb-interacting protein, putative | DIV |  | 0.759 |
| AAEL006909 | hypothetical protein | DIV |  | -0.751 |
| AAEL006946 | chaperonin | DIV | 0.754 |  |
| AAEL006972 | hepatocellular carcinoma-associated antigen | DIV | 0.785 |  |
| AAEL006987 | conserved hypothetical protein | DIV | 0.932 |  |
| AAEL007072 | conserved hypothetical protein | DIV | -1.336 |  |
| AAEL007114 | conserved hypothetical protein | DIV | -1.152 |  |
| AAEL007229 | conserved hypothetical protein | DIV | 0.864 | -1.01 |
| AAEL007239 | conserved hypothetical protein | DIV | 0.79 |  |
| AAEL007251 | zinc finger protein, putative | DIV | 0.808 |  |
| AAEL007256 | DNA helicase recq1 | DIV |  | 0.844 |
| AAEL007262 | hypothetical protein | DIV | 1.238 | -0.944 |
| AAEL007267 | suppressor of ty3 | DIV |  | 1.279 |
| AAEL007334 | glial maturation factor | DIV |  | 0.758 |
| AAEL007367 | conserved hypothetical protein | DIV | -0.821 |  |
| AAEL007375 | pyruvate dehydrogenase | DIV | -0.808 |  |
| AAEL007387 | conserved hypothetical protein | DIV | -1.127 |  |
| AAEL007452 | conserved hypothetical protein | DIV |  | 0.965 |
| AAEL007484 | protein transport protein sec23 | DIV |  | -1.013 |
| AAEL007488 | hypothetical protein | DIV |  | 1.503 |
| AAEL007489 | synaptic vesicle protein | DIV | 0.838 |  |
| AAEL007494 | calcineurin b subunit | DIV | 1.144 | 0.882 |
| AAEL007501 | nikI, nikkomycin biosynthesis protein P6, putative | DIV |  | 1.111 |
| AAEL007510 | transferase activity, transferring acyl groups other than amino-acyl groups | DIV | 0.87 |  |
| AAEL007536 | carnitine o-acyltransferase | DIV | 1.035 |  |
| AAEL007561 | conserved hypothetical protein | DIV | 0.828 |  |
| AAEL007581 | Rfc5p, putative | DIV | 1.069 |  |
| AAEL007621 | conserved hypothetical protein | DIV |  | 1.823 |
| AAEL007697 | conserved hypothetical protein | DIV |  | -0.835 |
| AAEL007738 | app binding protein | DIV | 1.183 |  |
| AAEL007800 | cytidine and deoxycytidylate deaminase zinc-binding region | DIV | -1.04 |  |
| AAEL007802 | multicopper oxidase | DIV |  | 1.094 |
| AAEL007822 | ubiquitin-conjugating enzyme E2 g | DIV | 2.209 | 1.057 |
| AAEL007842 | conserved hypothetical protein | DIV |  | 0.959 |
| AAEL007864 | zinc finger protein | DIV |  | 0.756 |
| AAEL007868 | ubiquinol-cytochrome c reductase complex 14 kd protein | DIV | -1.396 | 1.375 |
| AAEL007895 | beta-1,4-galactosyltransferase | DIV |  | -1.159 |
| AAEL007997 | conserved hypothetical protein | DIV | 0.933 |  |
| AAEL008135 | conserved hypothetical protein | DIV | 1.795 | 1.442 |
| AAEL008179 | RAS, putative | DIV |  | 0.789 |
| AAEL008279 | conserved hypothetical protein | DIV | -0.762 |  |
| AAEL008307 | conserved hypothetical protein | DIV | 0.813 | 1.583 |
| AAEL008369 | acylphosphatase, putative | DIV | 1.172 |  |
| AAEL008458 | conserved hypothetical protein | DIV |  | 2.34 |
| AAEL008473 | cysteinech venom protein, putative | DIV | 0.918 | 0.762 |
| AAEL008477 | hypothetical protein | DIV | -0.923 |  |
| AAEL008657 | midline fasciclin | DIV | 0.821 |  |
| AAEL008688 | g-protein signalling modulator | DIV |  | 1.036 |
| AAEL008723 | conserved hypothetical protein | DIV | 0.898 | 3.703 |
| AAEL008753 | conserved hypothetical protein | DIV |  | -2.148 |
| AAEL008793 | conserved hypothetical protein | DIV | -1.384 |  |
| AAEL008857 | deoxyribonuclease I, putative | DIV | 1.22 |  |
| AAEL008861 | deoxyribonuclease I, putative | DIV | 1.093 | 2.527 |
| AAEL008876 | deoxyribonuclease I, putative | DIV | 2.016 | 1.209 |
| AAEL008882 | conserved hypothetical protein | DIV | 0.75 |  |
| AAEL008900 | p15-2a protein, putative | DIV | 1.615 |  |
| AAEL008963 | tyrosine aminotransferase | DIV |  | 0.811 |
| AAEL009071 | conserved hypothetical protein | DIV | -1.438 |  |
| AAEL009094 | conserved hypothetical protein | DIV | 0.883 |  |
| AAEL009109 | conserved hypothetical protein | DIV | -1.49 |  |
| AAEL009166 | conserved hypothetical protein | DIV | 6.082 |  |
| AAEL009185 | arginine or creatine kinase | DIV | 0.843 |  |
| AAEL009198 | conserved hypothetical protein | DIV | -1.176 |  |
| AAEL009223 | conserved hypothetical protein | DIV |  | -0.758 |
| AAEL009326 | fragile X mental retardation syndromelated protein 1, putative | DIV |  | -0.862 |
| AAEL009393 | conserved hypothetical protein | DIV |  | 0.773 |
| AAEL009483 | conserved hypothetical protein | DIV | 0.822 | 1.81 |
| AAEL009531 | niemann-pick C1 | DIV |  | 0.793 |
| AAEL009561 | apolipoprotein D, putative | DIV | 3.317 |  |
| AAEL009626 | ankyrin repeat domain protein | DIV |  | 0.838 |
| AAEL009629 | endoU protein, putative | DIV |  | -0.996 |
| AAEL009719 | conserved hypothetical protein | DIV |  | -1.034 |
| AAEL009730 | hypothetical protein | DIV | -0.766 |  |
| AAEL009785 | protein kinase protein | DIV | 0.766 | 0.843 |
| AAEL009829 | ARL3, putative | DIV | 0.822 |  |
| AAEL009859 | nucleolar GTP-binding protein | DIV | -1.194 |  |
| AAEL009871 | 80 kda MCM3-associated protein | DIV | -0.773 |  |
| AAEL009875 | alanine aminotransferase | DIV |  | -1.324 |
| AAEL009909 | cln3/battenin | DIV |  | 1.02 |
| AAEL009912 | conserved hypothetical protein | DIV |  | 2.644 |
| AAEL009931 | arsenite inducuble RNA associated protein aip-1 | DIV | -0.935 | 1.011 |
| AAEL009935 | conserved hypothetical protein | DIV | 2.312 |  |
| AAEL010000 | ganglioside induced differentiation associated protein | DIV |  | 1.202 |
| AAEL010014 | hypothetical protein | DIV | 3.968 |  |
| AAEL010019 | conserved hypothetical protein | DIV | -1.087 | 1.197 |
| AAEL010054 | conserved hypothetical protein | DIV |  | 0.786 |
| AAEL010065 | protein disulfide-isomerase A6 precursor | DIV | 1.422 | -0.954 |
| AAEL010097 | nuclein acid binding | DIV | -0.84 |  |
| AAEL010123 | equilibrative nucleoside transporter | DIV | 0.756 |  |
| AAEL010137 | ketoreductase, putative | DIV | -1.645 | 2.167 |
| AAEL010204 | dihydropyrimidine dehydrogenase | DIV |  | 0.767 |
| AAEL010276 | aminomethyltransferase | DIV |  | -1.764 |
| AAEL010326 | conserved hypothetical protein | DIV | -0.876 |  |
| AAEL010339 | orf 140; significant similarity to ORF next to beta-keto adipate succinyl CoA transferase from P. putida, putative | DIV | 2.268 |  |
| AAEL010369 | phospholipase b, plb1 | DIV | -1.019 |  |
| AAEL010373 | dullard protein | DIV | 0.88 |  |
| AAEL010424 | p15-2a protein, putative | DIV |  | -0.751 |
| AAEL010431 | conserved hypothetical protein | DIV | -1.137 | 1.138 |
| AAEL010509 | bridging integrator | DIV | 0.757 |  |
| AAEL010513 | class b basic helix-loop-helix protein (bhlhb) (differentially expressed in chondrocytes) (mdec) (sharp) | DIV | -1.806 |  |
| AAEL010520 | conserved hypothetical protein | DIV | -0.802 |  |
| AAEL010588 | striatin, putative | DIV | -2.492 |  |
| AAEL010605 | zinc finger protein | DIV |  | -0.844 |
| AAEL010682 | armc4 | DIV |  | 0.805 |
| AAEL010775 | conserved hypothetical protein | DIV | 0.982 |  |
| AAEL010778 | conserved hypothetical protein | DIV | -0.85 |  |
| AAEL010899 | hypothetical protein | DIV | -0.893 |  |
| AAEL010920 | conserved hypothetical protein | DIV | -1.046 | -0.942 |
| AAEL010986 | apyrase, putative | DIV | -2.063 |  |
| AAEL011082 | conserved hypothetical protein | DIV | -1.363 |  |
| AAEL011105 | adducin | DIV | 1.198 |  |
| AAEL011134 | esophageal cancer associated protein | DIV |  | -1.368 |
| AAEL011135 | conserved hypothetical protein | DIV |  | -0.938 |
| AAEL011175 | alkaline phosphatase | DIV |  | 0.802 |
| AAEL011176 | hypothetical protein | DIV | -1.059 |  |
| AAEL011200 | conserved hypothetical protein | DIV | -0.775 |  |
| AAEL011261 | yemanuclein | DIV |  | -0.766 |
| AAEL011264 | phosphatidylethanolamine-binding protein | DIV |  | -0.765 |
| AAEL011383 | MAGE-41 protein, putative | DIV |  | 0.949 |
| AAEL011429 | clathrin coat adaptor ap3 medium chain | DIV | 1.13 |  |
| AAEL011510 | multiple inositol polyphosphate phosphatase | DIV |  | 2.572 |
| AAEL011518 | aspartyl-tRNA synthetase | DIV |  | 1.053 |
| AAEL011538 | conserved hypothetical protein | DIV | 0.819 | 0.807 |
| AAEL011606 | conserved hypothetical protein | DIV | 1.911 |  |
| AAEL011653 | thyroid hormone receptor interactor | DIV | 1.049 |  |
| AAEL011683 | hypothetical protein | DIV |  | -0.81 |
| AAEL011721 | conserved hypothetical protein | DIV |  | 1.128 |
| AAEL011782 | conserved hypothetical protein | DIV |  | -1.14 |
| AAEL011795 | allergen, putative | DIV | 1.854 | 2.733 |
| AAEL011798 | allergen, putative | DIV | 2.369 | 3.656 |
| AAEL011881 | conserved hypothetical protein | DIV | 1.201 |  |
| AAEL011899 | N-acetyllactosaminide beta-1,3-N-acetylglucosaminyltransferase, putative | DIV |  | 0.888 |
| AAEL011900 | N-acetyllactosaminide beta-1,3-N-acetylglucosaminyltransferase, putative | DIV | 0.906 |  |
| AAEL011962 | conserved hypothetical protein | DIV |  | 0.839 |
| AAEL011966 | conserved hypothetical protein | DIV | 0.871 |  |
| AAEL011971 | hypothetical protein | DIV | 1.2 | -1.028 |
| AAEL011980 | hypothetical protein | DIV |  | 1.369 |
| AAEL011989 | signal peptide peptidase | DIV | -2.234 |  |
| AAEL012071 | ras | DIV | 0.75 |  |
| AAEL012095 | 26S protease regulatory subunit | DIV |  | 1.017 |
| AAEL012097 | app binding protein | DIV | 1.228 |  |
| AAEL012105 | conserved hypothetical protein | DIV | 1.741 | 2.833 |
| AAEL012133 | conserved hypothetical protein | DIV | -0.851 |  |
| AAEL012223 | zinc finger protein | DIV |  | 0.841 |
| AAEL012233 | hypothetical protein | DIV |  | 1.03 |
| AAEL012260 | wdpeat protein | DIV | 1.068 | -0.835 |
| AAEL012262 | conserved hypothetical protein | DIV |  | 3.795 |
| AAEL012283 | diaphanous | DIV |  | 0.815 |
| AAEL012313 | charged multivesicular body protein 5 | DIV | 0.97 |  |
| AAEL012339 | cdk1 | DIV | 1.035 |  |
| AAEL012448 | conserved hypothetical protein | DIV |  | -0.961 |
| AAEL012532 | tetraspanin 29fb | DIV | -1.138 | 0.808 |
| AAEL012553 | tyrosine protein kinase | DIV |  | 1.033 |
| AAEL012554 | maltose phosphorylase | DIV | 1.734 |  |
| AAEL012567 | synaptic vesicle protein | DIV | 0.817 |  |
| AAEL012574 | hypothetical protein | DIV | 1.379 |  |
| AAEL012579 | aspartate aminotransferase | DIV |  | 0.903 |
| AAEL012635 | conserved hypothetical protein | DIV | 0.947 |  |
| AAEL012646 | conserved hypothetical protein | DIV | 1.091 | 0.837 |
| AAEL012687 | Juvenile hormone-inducible protein, putative | DIV |  | 1.062 |
| AAEL012703 | conserved hypothetical protein | DIV |  | -2.132 |
| AAEL012725 | conserved hypothetical protein | DIV | 0.848 |  |
| AAEL012737 | conserved hypothetical protein | DIV |  | 1.28 |
| AAEL012834 | hypothetical protein | DIV | 1.386 |  |
| AAEL012851 | wdpeat protein | DIV | 0.917 |  |
| AAEL012856 | hypothetical protein | DIV |  | -1.379 |
| AAEL012865 | conserved hypothetical protein | DIV |  | -0.981 |
| AAEL012866 | conserved hypothetical protein (acetyltransferase (GNAT) family domain) | DIV |  | 2.982 |
| AAEL012911 | conserved hypothetical protein | DIV | 0.796 |  |
| AAEL012996 | rho guanine dissociation factor | DIV | 0.818 | 2.387 |
| AAEL013008 | intraflagellar transport 52, putative | DIV |  | -0.855 |
| AAEL013062 | hypothetical protein | DIV |  | 0.831 |
| AAEL013072 | conserved hypothetical protein | DIV | -1.051 |  |
| AAEL013085 | hypothetical protein | DIV | -1.417 | -0.789 |
| AAEL013113 | metallo-beta-lactamase, putative | DIV |  | -0.924 |
| AAEL013118 | conserved hypothetical protein | DIV | -1.05 | 1.67 |
| AAEL013127 | conserved hypothetical protein | DIV | 5.817 | 3.24 |
| AAEL013223 | hypothetical protein | DIV | 1.955 |  |
| AAEL013327 | conserved hypothetical protein | DIV |  | 0.877 |
| AAEL013334 | conserved hypothetical protein | DIV |  | 0.89 |
| AAEL013338 | lethal(2)essential for life protein, l2efl | DIV | -1.5 | -1.493 |
| AAEL013351 | lethal(2)essential for life protein, l2efl | DIV |  | -0.951 |
| AAEL013559 | uncoordinated protein | DIV | 0.923 |  |
| AAEL013574 | apolipoprotein D, putative | DIV | 0.956 | 1.191 |
| AAEL013577 | conserved hypothetical protein | DIV | 3.14 | -0.928 |
| AAEL013584 | conserved hypothetical protein | DIV | -1.4 | 1.613 |
| AAEL013591 | dynactin, p27-subunit, putative | DIV | -1 |  |
| AAEL013594 | conserved hypothetical protein | DIV | -0.836 |  |
| AAEL013597 | conserved hypothetical protein | DIV | -0.957 |  |
| AAEL013612 | conserved hypothetical protein | DIV | 0.825 |  |
| AAEL013619 | dolichyl-diphosphooligosaccharide protein glycosyltransferase | DIV |  | -1.015 |
| AAEL013823 | calcium/calmodulin dependent protein kinase ii | DIV | -1.231 | -0.999 |
| AAEL013844 | diazepam binding inhibitor, putative | DIV | 0.997 |  |
| AAEL013857 | conserved hypothetical protein | DIV |  | -0.831 |
| AAEL013884 | synaptic vesicle protein | DIV | -0.758 | -0.805 |
| AAEL013962 | mutagen-sensitive, putative | DIV |  | 0.937 |
| AAEL014001 | yellow protein precursor, putative | DIV | 1.434 | 1.326 |
| AAEL014045 | allantoicase | DIV | -1.02 | 1.078 |
| AAEL014128 | hypothetical protein | DIV | -1.68 | -1.733 |
| AAEL014176 | cornichon, involved in development of anterior-posterior and dorso-ventral polarirty via EGF-receptor signaling pathway | DIV | 0.913 |  |
| AAEL014183 | conserved hypothetical protein | DIV | -1.152 | -0.792 |
| AAEL014275 | molybdopterin cofactor sulfurase (mosc) | DIV |  | 1.119 |
| AAEL014389 | conserved hypothetical protein | DIV |  | -1.267 |
| AAEL014399 | conserved hypothetical protein | DIV | -0.785 |  |
| AAEL014405 | predicted protein | DIV | 0.809 |  |
| AAEL014435 | Juvenile hormone-inducible protein, putative | DIV | 1.046 |  |
| AAEL014541 | maltose phosphorylase | DIV | 2.958 |  |
| AAEL014715 | 67 kDa polymerase-associated factor PAF67, putative | DIV | 1.586 |  |
| AAEL014734 | saccharopine dehydrogenase | DIV | -1.169 | -1.243 |
| AAEL014741 | serine/threonine-protein kinase ripk4 | DIV |  | 3.083 |
| AAEL014746 | o-linked n-acetylglucosamine transferase, ogt | DIV | -0.79 |  |
| AAEL014768 | glutamate synthase | DIV | -0.925 |  |
| AAEL014821 | hypothetical protein | DIV | 1.377 | 1.505 |
| AAEL014842 | multiple inositol polyphosphate phosphatase | DIV |  | 0.785 |
| AAEL014844 | conserved hypothetical protein | DIV | 1.228 |  |
| AAEL014868 | diaphanous | DIV |  | 1.184 |
| AAEL014901 | conserved hypothetical protein | DIV |  | 1.05 |
| AAEL014910 | synaptic vesicle protein | DIV | 1.534 |  |
| AAEL014938 | conserved hypothetical protein | DIV | -0.759 |  |
| AAEL014955 | tartan protein, putative | DIV | 1.039 |  |
| AAEL015107 | conserved hypothetical protein | DIV | 1.133 |  |
| AAEL015125 | Activin receptor type I, putative | DIV | -1.347 |  |
| AAEL015140 | conserved hypothetical protein | DIV | -1.189 | 1.933 |
| AAEL015172 | conserved hypothetical protein | DIV |  | -0.772 |
| AAEL015216 | serine/threonine-protein kinase vrk | DIV |  | -1.685 |
| AAEL015260 | phosphatidylethanolamine-binding protein, putative | DIV | -2.051 | 3.801 |
| AAEL015305 | sulfotransferase (sult) | DIV | 1.334 | 1.339 |
| AAEL015330 | hypothetical protein | DIV |  | -0.765 |
| AAEL015357 | conserved hypothetical protein | DIV |  | 1.232 |
| AAEL015415 | intracellular signaling pathway | DIV | 0.751 |  |
| AAEL015442 | hypothetical protein | DIV |  | 0.759 |
| AAEL015447 | hypothetical protein | DIV | -1.058 | -1.459 |
| AAEL015658 | conserved hypothetical protein | DIV |  | -0.889 |
| AAEL016973 | Conserved hypothetical protein (Chromatin organization modifier domain) | DIV | 0.914 |  |
| AAEL017029 | Phenylalanine hydroxylase | DIV |  | -1.094 |
| AAEL017421 | Nucleolar protein Nop56 , protein impliated in ribosomal biogenesis, translation, ribosomal structure and biogenesis | DIV | -1.235 | 0.918 |
| AAEL017480 | Ankyrin 2,3/unc44, akyrin repeats mediate protein-protein interactions in very diverse families of proteins, | DIV |  | 2.05 |
| AAEL017507 | catalytic activity | DIV |  | 0.762 |
| AAEL017549 | GPCR Orphan/Putative Class A Family [Source:Aedes_ManualAnnotation;Acc:AAEL800742] | DIV |  | 1.107 |
| AAEL000651 | alpha-amylase | DIG | -0.859 |  |
| AAEL000678 | alpha-amylase | DIG | -0.95 |  |
| AAEL001703 | serine-type enodpeptidase, putative | DIG | -1.127 | -1.176 |
| AAEL002347 | serine-type enodpeptidase, putative | DIG | -1.222 |  |
| AAEL003060 | serine-type enodpeptidase, putative | DIG | 4.278 | 0.967 |
| AAEL004369 | alpha-glucosidase | DIG |  | -1.252 |
| AAEL005188 | alpha-galactosidase/alpha-n-acetylgalactosaminidase | DIG |  | 1.378 |
| AAEL005460 | alpha-galactosidase/alpha-n-acetylgalactosaminidase | DIG | 1.053 |  |
| AAEL006181 | amidase | DIG | 1.166 |  |
| AAEL006365 | trypsin-alpha, putative | DIG |  | -1.301 |
| AAEL006384 | Trypsin, putative | DIG |  | -4.657 |
| AAEL006598 | serine-type enodpeptidase, putative | DIG | -1.47 |  |
| AAEL006627 | serine-type enodpeptidase, putative | DIG | 2.427 | 1.739 |
| AAEL006700 | Trypsin, putative | DIG | 1.975 |  |
| AAEL007601 | trypsin | DIG | -1.003 |  |
| AAEL007818 | trypsin | DIG |  | 0.94 |
| AAEL007992 | Trypsin, putative | DIG | 1.334 |  |
| AAEL008079 | trypsin-alpha, putative | DIG | 3.027 |  |
| AAEL008085 | trypsin | DIG | 1.615 |  |
| AAEL008093 | trypsin | DIG | 1.318 |  |
| AAEL008430 | conserved hypothetical protein | DIG | -0.752 | -1.884 |
| AAEL008781 | serine-type enodpeptidase, putative | DIG | 0.761 | 1.4 |
| AAEL010540 | alpha-amylase | DIG |  | -1.766 |
| AAEL011916 | serine-type enodpeptidase, putative | DIG | 1.774 | 1.38 |
| AAEL011929 | serine-type enodpeptidase, putative | DIG | 1.056 | 0.908 |
| AAEL012852 | trypsin | DIG | -1.158 |  |
| AAEL013262 | conserved hypothetical protein | DIG | -1.621 | 2.984 |
| AAEL013623 | trypsin | DIG |  | 0.809 |
| AAEL013628 | trypsin-eta, putative | DIG | 0.899 |  |
| AAEL014188 | serine-type enodpeptidase, putative | DIG | -0.855 |  |
| AAEL014361 | amidase | DIG | 1.142 |  |
| AAEL015102 | trypsin-alpha, putative | DIG |  | -1.415 |
| AAEL015528 | Trypsin, putative | DIG | -0.97 | -3.668 |
| AAEL017051 | Trypsin, putative | DIG | 1.277 | 1.025 |
| AAEL000598 | antibacterial peptide, putative | IMM |  | 1.003 |
| AAEL000621 | antibacterial peptide, putative | IMM | 1.182 | 0.876 |
| AAEL001914 | scavenger receptor, putative | IMM |  | 0.933 |
| AAEL002295 | leucinech transmembrane protein | IMM | 0.808 |  |
| AAEL002309 | peroxiredoxin 6, prx-6 | IMM | 1.439 | 1.605 |
| AAEL002593 | serine protease | IMM |  | -0.768 |
| AAEL003444 | caspase-1 | IMM | -1.465 | -0.768 |
| AAEL003520 | serine protease, putative | IMM |  | 1.564 |
| AAEL003723 | lysozyme P, putative | IMM | 0.882 |  |
| AAEL004120 | Niemann-Pick Type C-2, putative | IMM |  | -0.789 |
| AAEL004522 | gambicin | IMM | 0.803 | -0.769 |
| AAEL004868 | hemomucin | IMM |  | -0.778 |
| AAEL004884 | hemomucin | IMM | -1.169 |  |
| AAEL005482 | conserved hypothetical protein | IMM |  | -1.138 |
| AAEL005641 | galactose-specific C-type lectin, putative | IMM | 0.783 |  |
| AAEL005718 | serine protease, putative | IMM |  | 1.525 |
| AAEL005910 | programmed cell death protein | IMM | 0.903 | 0.809 |
| AAEL006378 | trypsin, putative | IMM |  | -3.395 |
| AAEL006389 | cathepsin l | IMM | -1.053 | -0.953 |
| AAEL006691 | fibrinogen and fibronectin | IMM | -1.519 | -0.965 |
| AAEL006699 | fibrinogen and fibronectin | IMM | -0.887 |  |
| AAEL006702 | fibrinogen and fibronectin | IMM | -2.051 | -1.576 |
| AAEL006704 | fibrinogen and fibronectin | IMM | 1.051 |  |
| AAEL007585 | cathepsin b | IMM | -1.988 | -1.488 |
| AAEL007599 | cathepsin b | IMM | -2.145 | -1.889 |
| AAEL007942 | fibrinogen and fibronectin | IMM |  | -0.812 |
| AAEL009176 | gram-negative bacteria binding protein | IMM |  | -1.436 |
| AAEL009178 | gram-negative bacteria binding protein | IMM |  | -1.17 |
| AAEL009556 | Niemann-Pick Type C-2, putative | IMM |  | 2.629 |
| AAEL009637 | cathepsin b | IMM |  | 3.552 |
| AAEL009680 | chymotrypsin, putative | IMM |  | -0.784 |
| AAEL009760 | Niemann-Pick Type C-2, putative | IMM | -1.839 |  |
| AAEL009842 | keratinocyte lectin, putative | IMM |  | -0.822 |
| AAEL010100 | lysozyme P, putative | IMM |  | -1.302 |
| AAEL010827 | programmed cell death protein 11 (prena processing protein rrp5) | IMM | 1.132 |  |
| AAEL011404 | galactose-specific C-type lectin, putative | IMM | 2.277 | 2.282 |
| AAEL011407 | type II transmembrane receptor OtB7, putative | IMM | 2.298 | 2.297 |
| AAEL011941 | oxidase/peroxidase | IMM | 0.797 |  |
| AAEL012216 | cathepsin b | IMM | -1.966 | -1.495 |
| AAEL012712 | trypsin, putative | IMM |  | 1.149 |
| AAEL013714 | trypsin, putative | IMM |  | 0.843 |
| AAEL014658 | caspase-1 | IMM |  | 0.944 |
| AAEL014989 | peptidoglycan recognition protein-1, putative | IMM | 0.994 |  |
| AAEL015312 | cathepsin b | IMM | -2.244 | -2.001 |
| AAEL015404 | lysozyme P, putative | IMM |  | -1.341 |
| AAEL017132 | C-Type Lysozyme (Lys-C). [Source:Aedes_ManualAnnotation;Acc:AAEL800171] | IMM | 2.179 |  |
| AAEL017211 | Cecropin, Anti-Microbial Peptide. [Source:Aedes_ManualAnnotation;Acc:AAEL800425] | IMM |  | -1.23 |
| AAEL017457 | Catalytic activity, serine-type endopeptidase activity, protelysis | IMM | 2.959 |  |
| AAEL017536 | Holotricin, Glycine Rich Repreat Protein (GRRP), Anti-Microbial Peptide. [Source:Aedes_ManualAnnotation;Acc:AAEL800434] | IMM |  | -1.706 |
| AAEL006736 | conserved hypothetical protein | IMM | -1.139 |  |
| AAEL010125 | mitotic protein phosphatase 1 regulator, putative | IMM |  | -0.962 |
| AAEL000006 | phosphoenolpyruvate carboxykinase | MET | -0.975 |  |
| AAEL000044 | ornithine decarboxylase | MET |  | 0.801 |
| AAEL000146 | nitrilase, putative | MET |  | 1.561 |
| AAEL000150 | nitrilase, putative | MET |  | 1.587 |
| AAEL000258 | magnesium ion binding, cytoplasm, lipid metabolic process, 5'-nucleotidase activity, lipase activty | MET | -0.779 |  |
| AAEL000687 | glucosyl/glucuronosyl transferases | MET | 1.135 |  |
| AAEL001146 | n-acetylgalactosaminyltransferase | MET | 1.195 |  |
| AAEL001151 | n-acetylgalactosaminyltransferase | MET | 1.678 | -1.138 |
| AAEL001232 | tubulointerstitial nephritis antigen | MET |  | 1.815 |
| AAEL001586 | glucosyl/glucuronosyl transferases | MET | 0.77 | 0.894 |
| AAEL001642 | 6-pyruvoyltetrahydropterin synthase, putative | MET | -0.849 |  |
| AAEL001816 | glucosyl/glucuronosyl transferases | MET | 0.822 |  |
| AAEL001837 | lipase | MET | 1.607 | 0.799 |
| AAEL002176 | inosine-uridine preferring nucleoside hydrolase | MET | 1.353 |  |
| AAEL002542 | triosephosphate isomerase | MET |  | 1.144 |
| AAEL002680 | AMP dependent ligase | MET | 1.123 | -1.7 |
| AAEL002842 | 3-hydroxyacyl-coa dehyrogenase | MET | 1.059 | 0.806 |
| AAEL002861 | catalytic activity, binding, metabolic process | MET | 1.486 |  |
| AAEL003622 | delta(9)-desaturase, putative | MET | 0.818 |  |
| AAEL003913 | methionine-tRNA synthetase | MET |  | -0.952 |
| AAEL003993 | cyclohex-1-ene-1-carboxyl-CoA hydratase, putative | MET | 0.789 |  |
| AAEL004059 | cystathionine beta-lyase | MET |  | -0.797 |
| AAEL004126 | sterol desaturase | MET | -1 |  |
| AAEL004137 | acyl-coa dehydrogenase | MET | 1.23 | 0.873 |
| AAEL004178 | ribose-phosphate pyrophosphokinase 1, putative | MET | -0.824 |  |
| AAEL004294 | dihydrolipoamide acetyltransferase component of pyruvate dehydrogenase | MET | 0.975 |  |
| AAEL004345 | cysteinyl-tRNA synthetase | MET | 1.364 |  |
| AAEL004427 | prefoldin subunit | MET | -0.763 |  |
| AAEL004507 | valacyclovir hydrolase | MET | -1.75 |  |
| AAEL004739 | acyl-coa dehydrogenase | MET | -0.962 | 1.558 |
| AAEL004757 | cleavage and polyadenylation specificity factor | MET |  | 1.679 |
| AAEL004778 | acyl-coa dehydrogenase | MET | 1.461 | -0.894 |
| AAEL004933 | lysosomal acid lipase, putative | MET | 0.849 |  |
| AAEL005134 | arylsulfatase b | MET |  | -1.195 |
| AAEL005337 | carbonic anhydrase | MET | 1.247 |  |
| AAEL005617 | utp-glucose-1-phosphate uridylyltransferase 2 | MET |  | 0.987 |
| AAEL005732 | acyl-coa dehydrogenase | MET |  | 1.553 |
| AAEL005740 | AMP dependent ligase | MET | -0.81 |  |
| AAEL005749 | lysosomal alpha-mannosidase (mannosidase alpha class 2b member 1) | MET |  | -0.894 |
| AAEL005763 | lysosomal alpha-mannosidase (mannosidase alpha class 2b member 1) | MET | 2.428 |  |
| AAEL005793 | AMP dependent ligase | MET |  | -2.44 |
| AAEL006297 | venom allergen | MET | 0.886 | 1.169 |
| AAEL006381 | sphingomyelin phosphodiesterase | MET | 1.068 |  |
| AAEL006982 | lipase | MET | 1.186 |  |
| AAEL007028 | ceramidase | MET | -1.145 |  |
| AAEL007055 | lipase | MET | -0.791 | -0.896 |
| AAEL007063 | lipase | MET | -1.274 |  |
| AAEL007090 | 4-nitrophenylphosphatase | MET | -1.311 |  |
| AAEL007097 | 4-nitrophenylphosphatase | MET |  | -0.885 |
| AAEL007326 | ccr4-not transcription complex | MET |  | 0.994 |
| AAEL007707 | malate dehydrogenase | MET |  | -1.503 |
| AAEL007892 | xaa-pro aminopeptidase | MET | -1.505 |  |
| AAEL008216 | aconitase | MET | -1.746 |  |
| AAEL008330 | hexaprenyldihydroxybenzoate methyltransferase | MET | 0.827 | 1.146 |
| AAEL008367 | fk506-binding protein | MET | -0.756 |  |
| AAEL008467 | cysteine synthase | MET |  | 0.832 |
| AAEL008507 | srpk | MET | 0.935 | 0.89 |
| AAEL008841 | acyl-CoA oxidase | MET |  | -0.785 |
| AAEL008849 | selenophosphate synthase | MET | -1.178 |  |
| AAEL008853 | choline/ethanolamine kinase | MET | 0.967 |  |
| AAEL008865 | oligoribonuclease, mitochondrial | MET | 0.778 |  |
| AAEL009038 | prolylcarboxypeptidase, putative | MET | -1.201 | -1.345 |
| AAEL009246 | glycoside hydrolases | MET | -1.324 |  |
| AAEL009291 | retinoid-inducible serine carboxypeptidase (serine carboxypeptidase | MET | 1.174 |  |
| AAEL009323 | carbonic anhydrase precursor | MET |  | 0.757 |
| AAEL009462 | hydroxyacylglutathione hydrolase | MET | 0.902 |  |
| AAEL009503 | 4-nitrophenylphosphatase | MET | 0.929 |  |
| AAEL009596 | sterol o-acyltransferase | MET | 0.828 |  |
| AAEL009664 | aldehyde dehydrogenase | MET | -0.813 | -0.867 |
| AAEL009764 | xaa-pro aminopeptidase | MET | -1.605 |  |
| AAEL009831 | pyrroline-5-carboxylate reductase | MET | -0.808 |  |
| AAEL009948 | aldehyde dehydrogenase | MET | 1.096 |  |
| AAEL009951 | dimeric dihydrodiol dehydrogenase | MET |  | -0.793 |
| AAEL009955 | conserved hypothetical protein | MET | -1.549 | -1.983 |
| AAEL010227 | dolichol-phosphate mannosyltransferase | MET | 1.502 | 0.916 |
| AAEL010269 | venom allergen | MET | 0.816 | 1.177 |
| AAEL010321 | porphobilinogen deaminase | MET | 0.865 |  |
| AAEL010442 | 4-hydroxyphenylpyruvate dioxygenase | MET |  | -1.071 |
| AAEL010555 | sterol regulatory element-binding protein | MET |  | -1.061 |
| AAEL010590 | aldose-1-epimerase | MET |  | -1.872 |
| AAEL010600 | hypoxia associated factor | MET | 0.998 | 1.077 |
| AAEL010653 | alpha-l-fucosidase | MET |  | -2.522 |
| AAEL010727 | glutaminyl-peptide cyclotransferase | MET | 0.807 |  |
| AAEL010893 | carbonic anhydrase | MET | 1.052 |  |
| AAEL010912 | dipeptidyl-peptidase | MET | -1.045 | -0.895 |
| AAEL010930 | l-asparaginase | MET | -0.957 |  |
| AAEL010936 | gamma glutamyl transpeptidases | MET | -0.8 |  |
| AAEL010960 | xaa-pro dipeptidase app(e.coli) | MET |  | 0.754 |
| AAEL011313 | epoxide hydrolase | MET |  | 0.855 |
| AAEL011314 | epoxide hydrolase | MET |  | -0.978 |
| AAEL011746 | succinyl-coa synthetase beta chain | MET |  | -1.264 |
| AAEL011898 | 1-acyl-sn-glycerol-3-phosphate acyltransferase | MET | -0.766 |  |
| AAEL011912 | mannose-1-phosphate guanyltransferase | MET | -0.995 |  |
| AAEL011973 | fumarylacetoacetate hydrolase | MET | 0.771 |  |
| AAEL012014 | l-lactate dehydrogenase | MET |  | 0.785 |
| AAEL012077 | nad dehydrogenase | MET | -1.307 |  |
| AAEL012162 | aldehyde dehydrogenase | MET | -0.871 |  |
| AAEL012340 | lipase 1 precursor | MET | -0.995 |  |
| AAEL012341 | lysosomal acid lipase, putative | MET |  | 0.898 |
| AAEL012402 | elongase, putative | MET | 1.093 | 0.843 |
| AAEL012425 | sterol desaturase | MET | 0.894 |  |
| AAEL012430 | AMP dependent ligase | MET |  | -0.982 |
| AAEL012431 | AMP dependent ligase | MET | 2.836 |  |
| AAEL012695 | sterol carrier protein-2, putative | MET |  | -2.194 |
| AAEL012697 | sterol carrier protein-2, putative | MET |  | -0.882 |
| AAEL012809 | peptidylprolyl isomerase | MET |  | -1.381 |
| AAEL012825 | bifunctional purine biosynthesis protein | MET | -1.776 |  |
| AAEL013047 | sphingolipid delta 4 desaturase/c-4 hydroxylase protein des2 | MET |  | 0.856 |
| AAEL013213 | enhancer of zeste, ezh | MET |  | 0.872 |
| AAEL013260 | alpha methylacyl-coa racemase | MET |  | -0.779 |
| AAEL013458 | glutamine synthetase 1, 2 (glutamate-amonia ligase) (gs) | MET |  | -1.447 |
| AAEL013717 | sphingomyelin phosphodiesterase | MET | 1.269 |  |
| AAEL013967 | Methylmalonyl-CoA carboxyltransferase 12S subunit, putative | MET |  | 1.063 |
| AAEL014026 | 1-acylglycerol-3-phosphate acyltransferase | MET | 1.585 |  |
| AAEL014080 | aldehyde dehydrogenase | MET |  | -0.886 |
| AAEL014101 | pyrroline-5-carboxylate reductase | MET | -1.058 |  |
| AAEL014190 | elongase, putative | MET | 1.335 | 0.98 |
| AAEL014204 | 6-phosphogluconolactonase | MET |  | -2.568 |
| AAEL014215 | nicotinate phosphoribosyltransferase | MET | -0.798 |  |
| AAEL014218 | lipase 1 precursor | MET | 1.535 | 1.358 |
| AAEL014219 | nicotinate phosphoribosyltransferase | MET | -0.873 |  |
| AAEL014246 | glucosyl/glucuronosyl transferases | MET |  | -1.067 |
| AAEL014426 | glycine dehydrogenase | MET |  | -1.138 |
| AAEL014449 | ribose-phosphate pyrophosphokinase 1, putative | MET | -0.893 |  |
| AAEL014452 | acyl-coa dehydrogenase | MET | -0.755 |  |
| AAEL014551 | triacylglycerol lipase, pancreatic | MET |  | -0.883 |
| AAEL014567 | oviductin | MET |  | 0.752 |
| AAEL014570 | oviductin | MET |  | 1.696 |
| AAEL014600 | 4-hydroxyphenylpyruvate dioxygenase | MET |  | -1.145 |
| AAEL014651 | nad dependent epimerase/dehydratase | MET | -0.918 |  |
| AAEL014664 | AMP dependent coa ligase | MET | 1.994 | 1.256 |
| AAEL014709 | methionine-tRNA synthetase | MET | -0.752 | 2.535 |
| AAEL014921 | lipase 1 precursor | MET |  | 0.788 |
| AAEL015020 | glycoside hydrolases | MET | -0.942 |  |
| AAEL015337 | neutral alpha-glucosidase ab precursor (glucosidase ii alpha subunit) (alpha glucosidase 2) | MET |  | -1.131 |
| AAEL015561 | gamma glutamyl transpeptidases | MET | -0.797 |  |
| AAEL015628 | glycine dehydrogenase | MET |  | -1.184 |
| AAEL015661 | sterol carrier protein-2, putative | MET |  | -1.143 |
| AAEL017133 | metabolic process, oxidoreductase activity | MET | 0.757 |  |
| AAEL017302 | NADP+-dependent farnesol dehydrogenase 1 | MET |  | 0.805 |
| AAEL017318 | Phosphofructokinase, putative | MET |  | 1.193 |
| AAEL001674 | serine-type enodpeptidase, putative | PROT | -1.039 | -1.119 |
| AAEL001690 | serine-type enodpeptidase, putative | PROT | 3.369 | -2.097 |
| AAEL001693 | serine-type enodpeptidase, putative | PROT | -0.78 |  |
| AAEL001839 | zinc carboxypeptidase | PROT | -1.277 | 1.859 |
| AAEL001844 | zinc carboxypeptidase | PROT | -0.95 |  |
| AAEL001863 | zinc carboxypeptidase | PROT |  | 1.019 |
| AAEL002360 | serine-type enodpeptidase, putative | PROT | -2.232 |  |
| AAEL002661 | matrix metalloproteinase | PROT | 0.75 |  |
| AAEL002793 | conserved hypothetical protein | PROT | -0.87 |  |
| AAEL003212 | protease m1 zinc metalloprotease | PROT | -0.783 |  |
| AAEL003951 | hypothetical protein | PROT |  | 0.788 |
| AAEL005808 | alanyl aminopeptidase | PROT |  | 1.282 |
| AAEL005821 | alanyl aminopeptidase | PROT |  | 2.643 |
| AAEL006011 | conserved hypothetical protein | PROT | 1.303 |  |
| AAEL006323 | hypothetical protein | PROT |  | -3.43 |
| AAEL006542 | retinoid-inducible serine carboxypeptidase (serine carboxypeptidase | PROT |  | -0.936 |
| AAEL006563 | retinoid-inducible serine carboxypeptidase (serine carboxypeptidase | PROT | -1.756 | -0.98 |
| AAEL007792 | conserved hypothetical protein | PROT | 1.956 | 1.8 |
| AAEL008155 | protease m1 zinc metalloprotease | PROT | -1.695 |  |
| AAEL008708 | lysosomal pro-X carboxypeptidase, putative | PROT | -0.905 |  |
| AAEL008780 | serine-type enodpeptidase, putative | PROT | -0.833 |  |
| AAEL009108 | protease m1 zinc metalloprotease | PROT | -1.095 | -1.828 |
| AAEL009310 | angiotensin-converting enzyme (dipeptidyl carboxypeptidase | PROT |  | 0.846 |
| AAEL009316 | angiotensin-converting enzyme (dipeptidyl carboxypeptidase | PROT | 0.995 |  |
| AAEL010782 | carboxypeptidase | PROT | -0.985 | 1.375 |
| AAEL011542 | metalloproteinase, putative | PROT |  | 1.071 |
| AAEL011557 | metalloproteinase, putative | PROT | 1.589 | 1.138 |
| AAEL011658 | plasma glutamate carboxypeptidase | PROT |  | -1.126 |
| AAEL011662 | plasma glutamate carboxypeptidase | PROT |  | -1.672 |
| AAEL011891 | serine-type enodpeptidase, putative | PROT | 0.764 |  |
| AAEL012776 | protease m1 zinc metalloprotease | PROT | -0.874 | 1.866 |
| AAEL012779 | protease m1 zinc metalloprotease | PROT | 1.45 |  |
| AAEL012783 | protease m1 zinc metalloprotease | PROT | -0.819 | 1.067 |
| AAEL012786 | conserved hypothetical protein | PROT | -0.76 |  |
| AAEL013899 | protease m1 zinc metalloprotease | PROT | -0.815 |  |
| AAEL014516 | metalloproteinase, putative | PROT |  | 1.261 |
| AAEL014671 | protease S51 alpha-aspartyl dipeptidase | PROT | -2.513 | 2.002 |
| AAEL014946 | protease U48 caax prenyl protease rce1 | PROT |  | 1.355 |
| AAEL017346 | Protease m1 zinc metalloprotease, putative (protease m1 zinc metalloprotease [Culex quinquefasciatus]) | PROT |  | 0.774 |
| AAEL002612 | hypothetical protein | RSM | 1.352 | 0.797 |
| AAEL003884 | conserved hypothetical protein | RSM | -0.958 |  |
| AAEL008606 | conserved hypothetical protein | RSM |  | 0.969 |
| AAEL010592 | esterase, putative | RSM | 1.015 | 1.065 |
| AAEL010634 | hypothetical protein | RSM |  | -1.079 |
| AAEL000502 | nadph fad oxidoreductase | RSM | 0.843 |  |
| AAEL000986 | NADH-ubiquinone oxidoreductase ashi subunit | RSM | 1.047 | 1.603 |
| AAEL001078 | glutathione-s-transferase theta, gst | RSM |  | -1.349 |
| AAEL001424 | mitochondrial inner membrane protease subunit | RSM | 1.006 |  |
| AAEL001960 | cytochrome P450 | RSM | 5.878 |  |
| AAEL002145 | gonadotropin inducible transcription factor | RSM |  | 1.009 |
| AAEL002385 | carboxylesterase | RSM | -1.119 |  |
| AAEL002493 | short-chain dehydrogenase | RSM | 0.759 |  |
| AAEL002723 | peroxisomal membrane protein pmp34 | RSM | 1.867 |  |
| AAEL003148 | short-chain dehydrogenase | RSM | 0.863 | 0.848 |
| AAEL003397 | mitochondrial ribosomal protein L17 | RSM | 0.998 |  |
| AAEL003423 | NADH dehydrogenase, putative | RSM |  | -1.032 |
| AAEL004054 | cytochrome P450 | RSM |  | -1.059 |
| AAEL004118 | aldo-keto reductase | RSM |  | -1.208 |
| AAEL004146 | DNA photolyase | RSM | 1.016 |  |
| AAEL004273 | short-chain dehydrogenase | RSM | 0.973 |  |
| AAEL004643 | mitochondrial ribosomal protein L1 | RSM |  | -1.151 |
| AAEL004870 | cytochrome P450 | RSM | 0.924 | 0.845 |
| AAEL005946 | NADH-ubiquinone oxidoreductase subunit B14.5b | RSM |  | 0.814 |
| AAEL006318 | short-chain dehydrogenase | RSM | 0.856 | 1.137 |
| AAEL006984 | cytochrome P450 | RSM | -1.378 |  |
| AAEL006989 | cytochrome P450 | RSM | 1.556 |  |
| AAEL007135 | peroxiredoxin 5, prdx5 | RSM | -0.971 |  |
| AAEL007473 | cytochrome P450 | RSM | -0.762 |  |
| AAEL007668 | mitochondrial ribosomal protein, S29, putative | RSM |  | -0.827 |
| AAEL007669 | oxidoreductase | RSM |  | 0.909 |
| AAEL007752 | cytochrome c oxidase, subunit VIIA, putative | RSM |  | -1.045 |
| AAEL007811 | short-chain dehydrogenase | RSM | -1.589 |  |
| AAEL007812 | cytochrome P450 | RSM | -0.95 | -0.841 |
| AAEL007830 | cytochrome P450 | RSM |  | -0.754 |
| AAEL007948 | glutathione-s-transferase theta, gst | RSM | -1.275 |  |
| AAEL007951 | glutathione-s-transferase theta, gst | RSM | 1.166 |  |
| AAEL008016 | short-chain dehydrogenase | RSM |  | 0.926 |
| AAEL008081 | excision repair cross-complementing 1 ercc1 | RSM |  | -1.516 |
| AAEL008345 | cytochrome P450 | RSM |  | -0.99 |
| AAEL008397 | glutathione peroxidase | RSM |  | -2.77 |
| AAEL008494 | mitochondrial carrier protein ymc | RSM |  | -0.904 |
| AAEL008532 | carboxylesterase | RSM |  | -1.083 |
| AAEL008663 | aldo-keto reductase | RSM | -0.908 |  |
| AAEL008846 | cytochrome P450 | RSM | -1.126 |  |
| AAEL008864 | conserved hypothetical protein | RSM | 0.754 |  |
| AAEL009100 | mitochondrial oxodicarboxylate carrier | RSM | 2.427 |  |
| AAEL009130 | cytochrome P450 | RSM | -0.763 |  |
| AAEL009414 | NADH-ubiquinone oxidoreductase 39 kda subunit | RSM | -2.749 | -2.232 |
| AAEL010382 | aldehyde oxidase | RSM | 0.859 |  |
| AAEL010384 | aldehyde oxidase | RSM | 0.817 |  |
| AAEL010389 | alpha-esterase | RSM | 1.497 |  |
| AAEL011239 | short-chain dehydrogenase | RSM | -1.109 |  |
| AAEL011704 | heat shock protein | RSM |  | 0.928 |
| AAEL011780 | DNA mismatch repair protein muts | RSM |  | 0.875 |
| AAEL011992 | NADH:ubiquinone dehydrogenase, putative | RSM |  | 0.92 |
| AAEL012318 | 2-amino-3-ketobutyrate coenzyme a ligase | RSM | 1.387 |  |
| AAEL012491 | cytochrome P450 | RSM | 1.549 | -0.975 |
| AAEL012509 | carboxylesterase-6 | RSM |  | -0.871 |
| AAEL012636 | cytochrome b5, putative | RSM | -1.095 | -1.104 |
| AAEL012761 | cytochrome P450 | RSM | 0.779 |  |
| AAEL013744 | NADH:ubiquinone dehydrogenase, putative | RSM | -1.215 |  |
| AAEL013771 | mitochondrial ribosomal protein, L54, putative | RSM |  | -0.811 |
| AAEL014412 | cytochrome P450 | RSM | -1.048 |  |
| AAEL014493 | aldehyde oxidase | RSM | 0.891 |  |
| AAEL014605 | cytochrome P450 | RSM | -0.772 |  |
| AAEL014606 | cytochrome P450 | RSM |  | 0.819 |
| AAEL014609 | cytochrome P450 | RSM | -0.791 |  |
| AAEL014611 | cytochrome P450 | RSM |  | 1.227 |
| AAEL014612 | cytochrome P450 | RSM |  | 0.788 |
| AAEL014673 | NADH:ubiquinone dehydrogenase, putative | RSM | 2.256 | 1.108 |
| AAEL014889 | NADH:ubiquinone dehydrogenase, putative | RSM | -0.908 |  |
| AAEL014891 | cytochrome P450 | RSM | 2.246 | -1.486 |
| AAEL014893 | cytochrome P450 | RSM | -1.238 |  |
| AAEL015218 | conserved hypothetical protein | RSM |  | 1.253 |
| AAEL015578 | alpha-esterase | RSM | 2.125 |  |
| AAEL017061 | Cytochrome p450, putative | RSM | 1.949 |  |
| AAEL017071 | Alpha-esterase, putative | RSM | 3.02 |  |
| AAEL017539 | cytochrome P450 [Source:Aedes_ManualAnnotation;Acc:AAEL800330] | RSM |  | 0.893 |
| AAEL000494 | histone h2a | RTT | 0.841 |  |
| AAEL000497 | histone h2a | RTT | 0.839 |  |
| AAEL000518 | histone h2a | RTT | 0.819 |  |
| AAEL000525 | histone h2a | RTT | 0.803 |  |
| AAEL001382 | small nuclear ribonucleoprotein sm d1 | RTT |  | 1.334 |
| AAEL001618 | ATP-dependent RNA helicase | RTT |  | 0.752 |
| AAEL001657 | DEAD box ATP-dependent RNA helicase | RTT |  | 0.815 |
| AAEL001769 | DEAD box ATP-dependent RNA helicase | RTT | -0.927 |  |
| AAEL002036 | nucleic acid binding | RTT | 0.896 | 0.846 |
| AAEL002488 | DEAD box ATP-dependent RNA helicase | RTT | 0.791 |  |
| AAEL002534 | 60S ribosomal protein L10 | RTT | -3.992 |  |
| AAEL003071 | tRNA pseudouridine synthase D | RTT |  | -0.841 |
| AAEL003352 | ribosomal protein l7ae | RTT |  | 0.974 |
| AAEL003396 | 60S ribosomal protein L32 | RTT | -1.42 |  |
| AAEL003427 | ribosomal protein S9, putative | RTT | -0.877 |  |
| AAEL003582 | ribosomal protein S15p/S13e | RTT |  | -1.336 |
| AAEL003589 | transcription factor, putative | RTT |  | -1.742 |
| AAEL003603 | transcription factor, putative | RTT |  | -2.172 |
| AAEL003716 | ribonuclease UK114, putative | RTT |  | -1.142 |
| AAEL004119 | ribonuclease p/mrp subunit | RTT | -0.792 |  |
| AAEL004252 | hypothetical protein | RTT | -0.97 |  |
| AAEL004254 | hypothetical protein | RTT | -0.937 |  |
| AAEL004325 | ribosomal protein L5 | RTT | -0.984 | -0.821 |
| AAEL004911 | DEAD box ATP-dependent RNA helicase | RTT | 0.854 | 1.043 |
| AAEL005097 | cold induced protein (BnC24A), putative | RTT | -1.136 |  |
| AAEL005127 | ribonuclease UK114, putative | RTT | 1.464 |  |
| AAEL005129 | 40S ribosomal protein S30 | RTT |  | 3.888 |
| AAEL005245 | DNA repair protein rad50 | RTT | 0.833 |  |
| AAEL005368 | transcription initiation factor TFIIB | RTT | 1.718 |  |
| AAEL005825 | translation initiation factor eif-2b beta subunit | RTT |  | -1.178 |
| AAEL005986 | hypothetical protein | RTT |  | -2.342 |
| AAEL006021 | hypothetical protein | RTT | 0.922 | 0.85 |
| AAEL006249 | poly(a) polymerase cid (pap) (caffein-induced death protein) | RTT | 1.594 |  |
| AAEL006698 | 60S ribosomal protein L31 | RTT | 1.356 |  |
| AAEL006851 | eukaryotic translation initiation factor | RTT |  | 0.781 |
| AAEL007005 | histone h2a | RTT | 0.824 |  |
| AAEL007078 | eukaryotic translation initiation factor 3, theta subunit | RTT |  | 1.848 |
| AAEL007482 | mediator complex, subunit, putative | RTT | -0.982 |  |
| AAEL008385 | Histone H2A, putative | RTT | 1.118 |  |
| AAEL008471 | hypothetical protein | RTT | 0.862 |  |
| AAEL009279 | conserved hypothetical protein | RTT | -0.87 |  |
| AAEL009465 | replication factor c / DNA polymerase iii gamma-tau subunit | RTT | -0.787 |  |
| AAEL009608 | 28S ribosomal protein S16, mitochondrial | RTT | -1.035 | 0.909 |
| AAEL009653 | 40S ribosomal protein S30 | RTT |  | 1.986 |
| AAEL010251 | translation initiation factor eif-2b alpha subunit | RTT | 1.467 |  |
| AAEL010467 | heterogeneous nuclear ribonucleoprotein | RTT |  | -0.768 |
| AAEL010821 | 60S acidic ribosomal protein P0 | RTT | 1.329 |  |
| AAEL011906 | histone H1, putative | RTT | -0.967 |  |
| AAEL011988 | tRNA selenocysteine associated protein (secp43) | RTT |  | 0.777 |
| AAEL012684 | conserved hypothetical protein | RTT |  | 0.78 |
| AAEL012686 | ribosomal protein S12, putative | RTT |  | 1.182 |
| AAEL012733 | 60S ribosomal protein L19 | RTT | -1.135 |  |
| AAEL013224 | hypothetical protein | RTT | 1.355 |  |
| AAEL013235 | ATP-dependent RNA helicase | RTT | 0.815 |  |
| AAEL013964 | ribosomal protein L20, putative | RTT |  | -0.861 |
| AAEL014292 | 40S ribosomal protein S24 | RTT | -0.909 |  |
| AAEL014330 | conserved hypothetical protein | RTT |  | 1.167 |
| AAEL014501 | mediator complex, subunit, putative | RTT | -0.934 |  |
| AAEL014748 | DNA repair protein rad50 | RTT | 0.787 |  |
| AAEL014764 | acidic ribosomal protein P1, putative | RTT | -1.155 |  |
| AAEL014903 | 40S ribosomal protein S24 | RTT | -0.76 |  |
| AAEL015236 | signal recognition particle, 9kD-subunit, putative | RTT | 0.939 | 1.004 |
| AAEL015244 | splicing factor 3a | RTT | 1.766 |  |
| AAEL015450 | ribonuclease UK114, putative | RTT |  | -1.041 |
| AAEL015674 | histone H2B | RTT | 0.761 |  |
| AAEL016129 | tRNA-Tyr | RTT |  | 0.762 |
| AAEL017363 | DNA-directed RNA polymerase II largest subunit, putative | RTT | -0.968 |  |
| AAEL017607 | U1 spliceosomal RNA [Source: RFAM 9.0] | RTT |  | -0.762 |
| AAEL017691 | U1 spliceosomal RNA [Source: RFAM 9.0] | RTT |  | -0.765 |
| AAEL017799 | U1 spliceosomal RNA [Source: RFAM 9.0] | RTT |  | -0.786 |
| AAEL017860 | U1 spliceosomal RNA [Source: RFAM 9.0] | RTT |  | -0.75 |
| AAEL017935 | U1 spliceosomal RNA [Source: RFAM 9.0] | RTT |  | -0.758 |
| AAEL000164 | d-amino acid oxidase | TRP | 0.823 |  |
| AAEL000213 | d-amino acid oxidase | TRP |  | -1.247 |
| AAEL000291 | Vacuolar ATP synthase 16 kDa proteolipid subunit | TRP |  | 0.892 |
| AAEL001101 | ATP-dependent transporter | TRP | 0.865 |  |
| AAEL001114 | amino acid transporter | TRP |  | -0.813 |
| AAEL001239 | cation efflux protein/ zinc transporter | TRP | 0.977 |  |
| AAEL001268 | conserved hypothetical protein | TRP |  | -0.839 |
| AAEL001318 | CRAL/TRIO domain-containing protein | TRP |  | -0.817 |
| AAEL001656 | sodium-dependent phosphate transporter | TRP | -1.249 | 1.013 |
| AAEL001687 | adenylate cyclase | TRP | -1.236 |  |
| AAEL001813 | sodium/solute symporter | TRP | 1.264 |  |
| AAEL001835 | sodium/solute symporter | TRP | 0.827 |  |
| AAEL002294 | sulfate transporter | TRP | 2.047 |  |
| AAEL002469 | endophilin a, putative | TRP | -1.052 |  |
| AAEL003347 | CRAL/TRIO domain-containing protein | TRP | -0.85 | -0.898 |
| AAEL003426 | sodium-dependent phosphate transporter | TRP | 1.307 |  |
| AAEL003627 | sodium/shloride dependent amino acid transporter | TRP | -0.794 |  |
| AAEL003758 | sorting nexin | TRP |  | 1.019 |
| AAEL003899 | sugar transporter | TRP | -0.888 |  |
| AAEL004213 | monocarboxylate transporter | TRP | 1.26 |  |
| AAEL004247 | Sialin, Sodium/sialic acid cotransporter, putative | TRP | 0.87 |  |
| AAEL004483 | hypothetical protein | TRP | 0.976 |  |
| AAEL004805 | potassium-dependent sodium-calcium exchanger, putative | TRP | 2.051 |  |
| AAEL004987 | conserved hypothetical protein | TRP | 0.961 | 1.348 |
| AAEL005001 | aquaporin | TRP | 1.179 | 1.551 |
| AAEL005499 | ATP-binding cassette transporter | TRP |  | -1.157 |
| AAEL006138 | hypothetical protein | TRP | -1.318 | -1.006 |
| AAEL006139 | sodium/solute symporter | TRP | -1.204 |  |
| AAEL006372 | sulfate transporter | TRP |  | -1.074 |
| AAEL006516 | vacuolar ATP synthase subunit h | TRP | 0.82 | 1.801 |
| AAEL006995 | sodium/solute symporter | TRP | 1.227 |  |
| AAEL007126 | sugar transporter | TRP | 1.021 |  |
| AAEL007128 | sugar transporter | TRP | 1.123 |  |
| AAEL007139 | sugar transporter | TRP | 0.87 |  |
| AAEL007731 | Pep12p, putative | TRP | 1.034 |  |
| AAEL008146 | zinc transporter | TRP | 1.682 |  |
| AAEL008848 | ATP synthase gamma subunit | TRP | -0.811 |  |
| AAEL008931 | inwardly rectifying k+ channel, putative | TRP | 0.933 |  |
| AAEL009362 | cationic amino acid transporter | TRP | -0.758 |  |
| AAEL009479 | amino acid transporter | TRP |  | 0.824 |
| AAEL010027 | CRAL/TRIO domain-containing protein | TRP | -0.807 |  |
| AAEL010102 | tetraspanin, putative | TRP | 1.03 |  |
| AAEL010148 | sodium/potassium-dependent atpase beta-2 subunit | TRP |  | -0.784 |
| AAEL010337 | CRAL/TRIO domain-containing protein | TRP | -0.774 |  |
| AAEL010479 | sugar transporter | TRP | -1.431 | -1.462 |
| AAEL010485 | sugar transporter | TRP | -1.335 |  |
| AAEL010584 | vesicular mannose-binding lectin | TRP | 0.79 |  |
| AAEL010738 | sodium bicarbonate cotransporter | TRP | 1.88 |  |
| AAEL011025 | vacuolar ATP synthase subunit ac39 | TRP |  | -1.202 |
| AAEL011244 | surfeit locus protein | TRP | -1.277 |  |
| AAEL011308 | serine threonine-protein kinase | TRP |  | -0.779 |
| AAEL011505 | integral membrane pore glycoprotein gp210, putative | TRP | 0.775 |  |
| AAEL012037 | sulfate transporter | TRP |  | 1.589 |
| AAEL012674 | d-amino acid oxidase | TRP | 1.629 |  |
| AAEL012960 | importin alpha | TRP | 0.777 |  |
| AAEL012981 | sugar transporter | TRP | 0.831 |  |
| AAEL013230 | coatomer delta subunit | TRP | 0.787 |  |
| AAEL013608 | sugar transporter | TRP | -0.84 |  |
| AAEL013848 | hypothetical protein | TRP | 1.346 |  |
| AAEL014156 | zinc transporter | TRP | 1.341 |  |
| AAEL014762 | zinc transporter | TRP | -0.755 | -2.569 |
| AAEL015001 | coatomer | TRP | 0.84 |  |
| AAEL015249 | sulfate transporter | TRP |  | -1.256 |
| AAEL016968 | Conserved hypothetical protein (xanthine-uracil permease superfamily) | TRP | 1.988 |  |
| AAEL000019 | conserved hypothetical protein | U | 1.028 | -1.165 |
| AAEL000310 | hypothetical protein | U |  | 1.357 |
| AAEL000607 | hypothetical protein | U |  | 0.984 |
| AAEL000612 | hypothetical protein | U |  | 0.972 |
| AAEL000658 | conserved hypothetical protein | U | -0.992 |  |
| AAEL000903 | hypothetical protein (enhancer of yellow 2 [Culex quinquefasciatus]) | U | -0.883 |  |
| AAEL000963 | conserved hypothetical protein | U |  | -0.759 |
| AAEL000992 | hypothetical protein | U |  | -0.8 |
| AAEL001107 | hypothetical protein | U | -0.963 |  |
| AAEL001261 | hypothetical protein | U | 0.768 |  |
| AAEL001414 | conserved hypothetical protein | U |  | -1.387 |
| AAEL001425 | conserved hypothetical protein | U | 0.981 |  |
| AAEL001499 | conserved hypothetical protein | U | -1.197 |  |
| AAEL001577 | conserved hypothetical protein | U |  | -0.87 |
| AAEL001596 | conserved hypothetical protein | U | 2.45 |  |
| AAEL001599 | conserved hypothetical protein | U | 0.982 |  |
| AAEL001880 | conserved hypothetical protein | U |  | 0.785 |
| AAEL001885 | conserved hypothetical protein | U |  | 0.791 |
| AAEL001892 | conserved hypothetical protein | U | -1.229 |  |
| AAEL001897 | conserved hypothetical protein | U |  | -1.16 |
| AAEL001913 | conserved hypothetical protein | U | -0.773 | -0.791 |
| AAEL001944 | conserved hypothetical protein | U | 1.263 |  |
| AAEL002056 | conserved hypothetical protein | U |  | 0.941 |
| AAEL002072 | conserved hypothetical protein | U | 0.876 |  |
| AAEL002100 | hypothetical protein | U |  | -0.809 |
| AAEL002315 | hypothetical protein | U |  | -0.859 |
| AAEL002429 | hypothetical protein | U | 0.774 | 1.725 |
| AAEL002641 | conserved hypothetical protein | U | -0.845 |  |
| AAEL002719 | conserved hypothetical protein | U | -0.935 |  |
| AAEL002742 | hypothetical protein | U | 1.793 |  |
| AAEL002805 | conserved hypothetical protein | U |  | 0.931 |
| AAEL002811 | conserved hypothetical protein | U |  | 4.123 |
| AAEL002889 | hypothetical protein | U | 2.811 |  |
| AAEL002900 | conserved hypothetical protein | U | 1.1 | 0.836 |
| AAEL002935 | conserved hypothetical protein | U |  | 1.016 |
| AAEL002944 | conserved hypothetical protein | U |  | 0.964 |
| AAEL002979 | conserved hypothetical protein | U | 1.081 |  |
| AAEL003029 | hypothetical protein | U |  | -0.807 |
| AAEL003107 | conserved hypothetical protein | U |  | -1.246 |
| AAEL003160 | conserved hypothetical protein | U | -3.322 |  |
| AAEL003190 | hypothetical protein | U | 0.762 |  |
| AAEL003264 | conserved hypothetical protein | U | 0.869 |  |
| AAEL003333 | hypothetical protein | U | -1.14 | -1.284 |
| AAEL003457 | conserved hypothetical protein | U |  | -1.197 |
| AAEL003483 | conserved hypothetical protein | U |  | -1.716 |
| AAEL003585 | conserved hypothetical protein | U |  | 1.235 |
| AAEL003590 | hypothetical protein | U | -1.033 |  |
| AAEL003596 | hypothetical protein | U | -1.244 | -1.494 |
| AAEL003601 | conserved hypothetical protein | U |  | 0.979 |
| AAEL003749 | conserved hypothetical protein | U |  | 1.518 |
| AAEL003816 | hypothetical protein | U | -1.273 |  |
| AAEL003878 | hypothetical protein | U |  | -0.837 |
| AAEL003987 | hypothetical protein | U |  | 1.296 |
| AAEL004239 | conserved hypothetical protein | U | -0.954 | -1.936 |
| AAEL004317 | hypothetical protein | U | 0.818 | -0.769 |
| AAEL004373 | hypothetical protein | U |  | 0.841 |
| AAEL004411 | hypothetical protein | U |  | 0.828 |
| AAEL004541 | conserved hypothetical protein | U |  | -1.12 |
| AAEL004558 | conserved hypothetical protein | U |  | -1.268 |
| AAEL004591 | hypothetical protein | U |  | -1.108 |
| AAEL004689 | conserved hypothetical protein | U |  | 0.88 |
| AAEL004775 | conserved hypothetical protein | U |  | 0.773 |
| AAEL004826 | conserved hypothetical protein | U |  | -1.456 |
| AAEL004841 | conserved hypothetical protein | U | -0.81 |  |
| AAEL005100 | hypothetical protein | U | -0.757 |  |
| AAEL005103 | conserved hypothetical protein | U | 1.132 |  |
| AAEL005156 | hypothetical protein | U |  | 1.352 |
| AAEL005158 | conserved hypothetical protein | U | 1.646 |  |
| AAEL005179 | hypothetical protein | U |  | -0.866 |
| AAEL005212 | hypothetical protein | U | -0.81 |  |
| AAEL005259 | conserved hypothetical protein | U |  | -0.982 |
| AAEL005315 | conserved hypothetical protein | U | 1.117 | 0.967 |
| AAEL005428 | conserved hypothetical protein | U |  | -2.873 |
| AAEL005517 | hypothetical protein | U | -1.218 |  |
| AAEL005601 | conserved hypothetical protein | U | 0.899 |  |
| AAEL005608 | hypothetical protein | U |  | -0.928 |
| AAEL005620 | conserved hypothetical protein | U |  | 1.417 |
| AAEL005669 | conserved hypothetical protein | U | -1.839 |  |
| AAEL005702 | conserved hypothetical protein | U | 0.814 |  |
| AAEL005755 | hypothetical protein | U | -0.954 | -0.987 |
| AAEL005899 | conserved hypothetical protein | U | 0.75 |  |
| AAEL005912 | conserved hypothetical protein | U |  | -0.854 |
| AAEL005968 | conserved hypothetical protein | U | 0.847 |  |
| AAEL006103 | conserved hypothetical protein | U |  | 0.814 |
| AAEL006146 | hypothetical protein | U | 1.158 |  |
| AAEL006220 | conserved hypothetical protein | U | 0.829 |  |
| AAEL006225 | conserved hypothetical protein | U | -1.25 |  |
| AAEL006228 | hypothetical protein | U |  | 1.435 |
| AAEL006247 | conserved hypothetical protein | U |  | 0.938 |
| AAEL006493 | hypothetical protein (putative salivary secreted peptide [Aedes albopictus]) | U |  | 1.194 |
| AAEL006528 | hypothetical protein | U | -0.922 |  |
| AAEL006549 | conserved hypothetical protein | U |  | -0.767 |
| AAEL006616 | hypothetical protein | U | -0.755 |  |
| AAEL006706 | conserved hypothetical protein | U |  | -0.886 |
| AAEL006863 | hypothetical protein | U | 1.369 | 2.98 |
| AAEL006890 | conserved hypothetical protein | U |  | -1.092 |
| AAEL006896 | hypothetical protein | U | 0.976 |  |
| AAEL006908 | conserved hypothetical protein | U | 0.825 |  |
| AAEL007012 | hypothetical protein | U |  | -0.826 |
| AAEL007071 | conserved hypothetical protein | U |  | -0.843 |
| AAEL007265 | hypothetical protein | U |  | 0.818 |
| AAEL007343 | conserved hypothetical protein | U | -0.928 |  |
| AAEL007381 | conserved hypothetical protein | U | -1.109 | -0.87 |
| AAEL007537 | conserved hypothetical protein | U | 1.996 |  |
| AAEL007666 | hypothetical protein | U |  | -0.85 |
| AAEL007667 | hypothetical protein | U |  | -1.489 |
| AAEL007680 | hypothetical protein | U | -0.762 |  |
| AAEL007703 | conserved hypothetical protein | U | 3.956 |  |
| AAEL007729 | conserved hypothetical protein | U | 0.93 |  |
| AAEL007847 | conserved hypothetical protein | U |  | 0.954 |
| AAEL007872 | hypothetical protein | U |  | 1.108 |
| AAEL007910 | conserved hypothetical protein | U |  | 0.786 |
| AAEL008024 | hypothetical protein | U |  | 0.758 |
| AAEL008025 | conserved hypothetical protein | U |  | 1.491 |
| AAEL008100 | conserved hypothetical protein | U |  | -1.003 |
| AAEL008248 | conserved hypothetical protein | U | 0.821 | -0.833 |
| AAEL008359 | hypothetical protein | U | 1.155 | 2.083 |
| AAEL008365 | conserved hypothetical protein | U |  | -1.075 |
| AAEL008433 | hypothetical protein | U |  | -1.62 |
| AAEL008441 | hypothetical protein | U | 1.049 | 0.817 |
| AAEL008446 | hypothetical protein | U | -0.772 | -0.817 |
| AAEL008460 | hypothetical protein | U | -1.418 |  |
| AAEL008478 | conserved hypothetical protein | U | -0.761 |  |
| AAEL008485 | conserved hypothetical protein | U | -1.252 | -2.198 |
| AAEL008492 | conserved hypothetical protein | U | 2.138 |  |
| AAEL008750 | conserved hypothetical protein | U | 1.825 |  |
| AAEL008761 | conserved hypothetical protein | U |  | 0.8 |
| AAEL008771 | conserved hypothetical protein | U |  | 1.272 |
| AAEL008910 | hypothetical protein | U | 0.957 |  |
| AAEL009103 | hypothetical protein | U | 0.8 |  |
| AAEL009165 | conserved hypothetical protein | U | -0.867 | 1.905 |
| AAEL009188 | hypothetical protein | U | 1.655 |  |
| AAEL009329 | hypothetical protein | U |  | 1.375 |
| AAEL009347 | hypothetical protein | U | 0.838 |  |
| AAEL009476 | hypothetical protein | U |  | 0.821 |
| AAEL009627 | hypothetical protein | U |  | -1.011 |
| AAEL009713 | conserved hypothetical protein | U | 0.761 |  |
| AAEL009844 | conserved hypothetical protein | U | 1.072 |  |
| AAEL009985 | conserved hypothetical protein (14.5 kDa salivary protein [Culex quinquefasciatus]) | U | -0.926 |  |
| AAEL010025 | conserved hypothetical protein | U | 1.036 |  |
| AAEL010072 | hypothetical protein | U |  | -1.246 |
| AAEL010385 | conserved hypothetical protein | U | 0.995 |  |
| AAEL010623 | conserved hypothetical protein | U |  | 0.769 |
| AAEL010892 | conserved hypothetical protein | U |  | -0.754 |
| AAEL010937 | conserved hypothetical protein | U |  | 0.791 |
| AAEL011148 | hypothetical protein | U |  | 0.891 |
| AAEL011262 | hypothetical protein | U |  | -0.842 |
| AAEL011456 | conserved hypothetical protein | U |  | 1.485 |
| AAEL011516 | conserved hypothetical protein | U |  | -0.857 |
| AAEL011551 | conserved hypothetical protein | U | -1.21 | -0.882 |
| AAEL011710 | conserved hypothetical protein | U | 0.915 |  |
| AAEL011799 | conserved hypothetical protein | U | -0.914 |  |
| AAEL011869 | conserved hypothetical protein | U | -0.868 | 0.878 |
| AAEL012012 | conserved hypothetical protein (phosphotyrosine-binding (PTB) domain) | U | 2.582 |  |
| AAEL012017 | hypothetical protein | U | -0.878 |  |
| AAEL012214 | hypothetical protein | U | -0.833 |  |
| AAEL012295 | conserved hypothetical protein | U | 0.892 |  |
| AAEL012438 | conserved hypothetical protein | U |  | 1.537 |
| AAEL012536 | hypothetical protein | U | -1.588 | -0.952 |
| AAEL012586 | conserved hypothetical protein | U | 0.925 | 0.981 |
| AAEL012639 | hypothetical protein | U | 3.161 |  |
| AAEL012704 | conserved hypothetical protein | U |  | -1.678 |
| AAEL012710 | conserved hypothetical protein | U |  | 1.504 |
| AAEL012837 | conserved hypothetical protein | U |  | -0.825 |
| AAEL012849 | hypothetical protein | U | 0.816 |  |
| AAEL012858 | hypothetical protein | U |  | -0.791 |
| AAEL012859 | conserved hypothetical protein | U | 1.072 |  |
| AAEL012931 | conserved hypothetical protein | U | -1.009 |  |
| AAEL013004 | conserved hypothetical protein | U | 1.47 | 1.547 |
| AAEL013013 | hypothetical protein | U |  | -0.765 |
| AAEL013207 | conserved hypothetical protein | U | 0.8 |  |
| AAEL013273 | hypothetical protein | U | 0.94 |  |
| AAEL013287 | conserved hypothetical protein (cystatin-like domain; cysteine-type endopeptidase inhibitor activity) | U | -0.855 |  |
| AAEL013290 | conserved hypothetical protein | U | -0.821 | -0.939 |
| AAEL013484 | hypothetical protein | U | 0.838 |  |
| AAEL013545 | hypothetical protein | U |  | -0.86 |
| AAEL013724 | conserved hypothetical protein | U |  | 1.288 |
| AAEL013774 | conserved hypothetical protein | U | 3.719 |  |
| AAEL013776 | predicted protein | U | 0.75 |  |
| AAEL013800 | conserved hypothetical protein | U | 0.754 |  |
| AAEL013905 | conserved hypothetical protein | U | -1.279 |  |
| AAEL013945 | conserved hypothetical protein | U | -0.98 |  |
| AAEL014171 | conserved hypothetical protein | U |  | -1.466 |
| AAEL014234 | conserved hypothetical protein | U | 1.855 | 1.779 |
| AAEL014241 | hypothetical protein | U | -0.886 |  |
| AAEL014268 | hypothetical protein | U |  | 0.992 |
| AAEL014293 | hypothetical protein | U | 0.835 | 1.119 |
| AAEL014388 | conserved hypothetical protein | U |  | -0.887 |
| AAEL014454 | hypothetical protein | U | 0.931 |  |
| AAEL014573 | conserved hypothetical protein | U | -0.79 |  |
| AAEL014736 | conserved hypothetical protein | U |  | 0.85 |
| AAEL014855 | conserved hypothetical protein | U | -0.752 |  |
| AAEL014898 | hypothetical protein | U |  | 0.991 |
| AAEL014937 | hypothetical protein | U | -0.808 | -2.122 |
| AAEL015014 | conserved hypothetical protein | U | -0.918 |  |
| AAEL015437 | conserved hypothetical protein | U | 1.021 |  |
| AAEL015451 | hypothetical protein | U |  | -0.988 |
| AAEL015477 | conserved hypothetical protein | U | 1.642 |  |
| AAEL015493 | conserved hypothetical protein | U | -0.875 | -1.129 |
| AAEL015616 | conserved hypothetical protein | U | -1.102 |  |
| AAEL016996 | Hypothetical protein | U |  | 2.075 |
| AAEL017016 | Conserved hypothetical protein | U | -1.456 |  |
| AAEL017034 | Hypothetical protein | U | 2.432 |  |
| AAEL017078 | Hypothetical protein | U |  | 1.065 |
| AAEL017087 | Hypothetical protein | U |  | 0.756 |
| AAEL017190 | Hypothetical protein | U |  | -1.297 |
| AAEL017455 | hypothetical protein | U |  | -1.154 |
| AAEL017468 | hypothetical protein | U |  | 0.823 |
| AAEL017491 | hypothetical protein | U | -0.942 |  |
| AAEL017519 | hypothetical protein | U |  | -1.1 |
