# Supplementary material for: Engineered Aedes aegypti JAK/STAT Pathway-Mediated Immunity to Dengue Virus
Source: PLoS Negl Trop Dis. 2017 Jan 12;11(1):e0005187. doi: 10.1371/journal.pntd.0005187 (PMC5230736; doi:10.1371/journal.pntd.0005187)
Supplement: S4 Table — (DOCX) [file pntd.0005187.s009.docx]

**Table S4. Log_2_-fold values of the putative RFs and HFs in the midgut transcriptome.**

| **Gene ID** | **Gene name** | **Putative role** | **VgDome MG** | **VgHop MG** |
| --- | --- | --- | --- | --- |
| AAEL007703 | conserved hypothetical protein | RF | 3.956 |  |
| AAEL012697 | sterol carrier protein-2, putative | HF |  | -0.882 |
| AAEL011025 | vacuolar ATP synthase subunit ac39 | HF |  | -1.202 |
