# Supplementary material for: Engineered Aedes aegypti JAK/STAT Pathway-Mediated Immunity to Dengue Virus
Source: PLoS Negl Trop Dis. 2017 Jan 12;11(1):e0005187. doi: 10.1371/journal.pntd.0005187 (PMC5230736; doi:10.1371/journal.pntd.0005187)
Supplement: S5 Table — (DOCX) [file pntd.0005187.s010.docx]

**Table S5. Descriptive statistics for CHIKV and ZIKV infection assays.**

|  | **CHIKV** | | | | | | | |
| --- | --- | --- | --- | --- | --- | --- | --- | --- |
|  | **Midgut 7dpibm** | | **Disseminated 7dpibm** | | **Midgut 14dpibm** | | **Disseminated 14dpibm** | |
|  | WT | VgHop | WT | VgHop | WT | VgHop | WT | VgHop |
| n | 61 | 47 | 61 | 46 | 34 | 22 | 34 | 22 |
| Median | 20000 | 300000 | 22000 | 5100 | 600000 | 260000 | 365000 | 210000 |
| Mean | 263182 | 613289 | 873990 | 912526 | 870315 | 548136 | 751912 | 530909 |
| SD | 469343 | 737824 | 1487000 | 1406000 | 1049000 | 862818 | 978745 | 1035000 |
|  |  |  |  |  |  |  |  |  |
|  |  |  |  |  |  |  |  |  |
|  |  |  |  |  |  |  |  |  |
|  | **ZIKV** | | | | | | | |
|  | **Midgut 7dpibm** | | **Disseminated 7dpibm** | | **Midgut 14dpibm** | | **Disseminated 14dpibm** | |
|  | WT | VgHop | WT | VgHop | WT | VgHop | WT | VgHop |
| n | 67 | 52 | 57 | 34 | 84 | 54 | 60 | 32 |
| Median | 45 | 3750 | 0 | 0 | 4250 | 7750 | 0 | 0 |
| Mean | 10461 | 14368 | 128895 | 0 | 19886 | 13288 | 4138 | 121.1 |
| SD | 19238 | 26443 | 532784 | 0 | 28614 | 18439 | 22514 | 354.4 |
