# Supplementary material for: Engineered Aedes aegypti JAK/STAT Pathway-Mediated Immunity to Dengue Virus
Source: PLoS Negl Trop Dis. 2017 Jan 12;11(1):e0005187. doi: 10.1371/journal.pntd.0005187 (PMC5230736; doi:10.1371/journal.pntd.0005187)
Supplement: S6 Table — (DOCX) [file pntd.0005187.s011.docx]

**Table S6. List of primers used to generate the transgene constructs, dsRNA synthesis, and real-time PCR.**

| **Gene / Segment** | **Primer Name** | **Sequence** | **RE sites / Notes** |
| --- | --- | --- | --- |
| Dome 1-1531 | Dome1F_PstI | TAGTCTGCAGATGGTACAGAGACAAGTATT | PstI |
|  | Dome1R_AclI | AGCTAACGTTGTTCAGTTCATAGCT | AclI |
| Dome 1532-3432 | Dome2F_AclI | GAACAACGTTAGCTCGACGTTAAATTTTGC | AclI |
|  | Dome2R_PstI | TAGTCTGCAGTTACTGCATTTTCAGACCAT | PstI |
| Hop 1-1516 | Hop1F_EcoRI | TATGAGAATTCATGTCGGAGCATGAGAACAAAT | EcoRI |
|  | Hop1R_SacI | GAAAGAGCTCAGCTCTTGTCCTTTCAAAGA | SacI |
| Hop 1517-3408 | Hop2F_SacI | AGCTGAGCTCTTTCTGCCGAATAATACCAA | SacI |
|  | Hop2R_EcoRI | TATGAGAATTCTTAGAAAAGTTGAATTGATT | EcoRI |
| Trypsin terminator | Tryp-Ter-F | TGAATACTAGTTAGGTAGCTGAGCGCATGCGATCTC | SpeI |
|  | Tryp-Ter-R | TAAGTGCGGCCGC***GGCCGGCC***GGTCGGCGCGCCCACCCTTGAG | NotI*,* ***FseI*** |
| AeVg promoter | AeVgPro F | TAGTCTCGAG***GGCCGGCC***GAATTCCACCACCAGG | XhoI*,* ***FseI*** |
|  | AeVgPro R | TAGTGTCGACCTTCAAGTATCCGGCAGCTG | SalI |
| Transgenic verification | ITRR2’ | GGGGTCCGTCAAAACAAAACA | used with VgProR |
| FBN | T7FBN13417-F436 | TAATACGACTCACTATAGGG ACCCTGGTTCCCGACAAATC | dsRNA synthesis |
|  | T7FBN13417-R845 | TAATACGACTCACTATAGGG TCCAAAGCATCACGAGCAGT |  |
|  | qFBN13417-F168 | AGCAGTGAACGCAGACATGA | realtime PCR |
|  | qFBN13417-R261 | GCGATGCGTGATCGTTGTTT |  |
| GAMB | T7GAMB4522-F58 | TAATACGACTCACTATAGGG ACCGATGCTTTGGTGTTTGTT | dsRNA synthesis |
|  | T7GAMB4522-R249 | TAATACGACTCACTATAGGG GTAGCATTCGGTGATGGCAC |  |
|  | qGAMB4522-F13 | ACAGTGTGTATTTTGCTGGCAC | realtime PCR |
|  | qGAMB4522-R65 | GCATCGGTATAGGCAGCTGAT |  |
| UKN7703 | T7UKN7703-F718 | TAATACGACTCACTATAGGG GGTCGGCTATCGGCAGTATC | dsRNA synthesis |
|  | T7UKN7703-R1164 | TAATACGACTCACTATAGGG CTCCAATCCCAGTTGGCTGT |  |
|  | qUKN7703-F144 | CGCTCGGAACTCGCTATCTT | realtime PCR |
|  | qUKN7703-R269 | GAATACACACCTCCCGCCAA |  |
| UNK566 | T7UKN566-F135 | TAATACGACTCACTATAGGGCAGACCTTCAGACGCTGCTA | dsRNA synthesis |
|  | T7UKN566-R413 | TAATACGACTCACTATAGGGACGTATGCCTTGCACCAATC |  |
|  | qUKN566-F5 | CGCAGACAATCAAGATAAGCGG | realtime PCR |
|  | qUKN566-99 | CAGCAACAGAACCCCTAGCA |  |
| DDX | T7DDX-F272 | TAATACGACTCACTATAGGGATGGCCACGAGAACGGATTT | dsRNA synthesis |
|  | T7DDX-R480 | TAATACGACTCACTATAGGGATCCGTGCCGTTCTCATTGT |  |
|  | qDDX-F1041 | TCGTCTGTTGGACTTCGTCG | realtime PCR |
|  | qDDX-R1150 | CAACCGATGGCATGAAACCC |  |
| SCP2 | T7SCP2-F71 | TAATACGACTCACTATAGGGAGGTTCTGGGAGTGTTCCAGT | dsRNA synthesis |
|  | T7SCP2-R233 | TAATACGACTCACTATAGGGAAGGTCTTTCCGCTGATGGC |  |
|  | qSCP2-F22 | GAGAGAATCAAGGCTCGCGT | realtime PCR |
|  | qSCP2-R107 | GCGGTCTTGATGTTCAACTGG​ |  |
| TEP22 | T7TEP22-87-F2302 | TAATACGACTCACTATAGGGTTGGGGGAAATCGCGATCAA | dsRNA synthesis |
|  | T7TEP22-87-R2769 | TAATACGACTCACTATAGGGGTTCCATTGACCAAACGCCC​ |  |
|  | qTEP22-87-F291 | TGTCAACGACGGTGGTAGTG | realtime PCR |
|  | qTEP22-87-R400 | CGCCTGGTTTGTAGACAGGT |  |
| Dome | RT Dome 2F | AAACGGTGGCAAAATGAACT | realtime PCR |
|  | RT Dome 2R | CTCCAGACCGGTGAGATTGT |  |
| Hop | Hop F | CCGGACTTTATCGAGCTGTC | realtime PCR |
|  | Hop R | ATCTGGTTCACTCCGTCGTC |  |
| DVRF1 | DVRF1 F | TAGTGCTGTTTGCCGGATTT | realtime PCR |
|  | DVRF1 R | TTCCTGATCCGTGATTCTCA |  |
