# Supplementary material for: Engineered Aedes aegypti JAK/STAT Pathway-Mediated Immunity to Dengue Virus
Source: PLoS Negl Trop Dis. 2017 Jan 12;11(1):e0005187. doi: 10.1371/journal.pntd.0005187 (PMC5230736; doi:10.1371/journal.pntd.0005187)
Supplement: S7 Table — (DOCX) [file pntd.0005187.s012.docx]

**Table S7. Descriptive statistics for DENV infection assays in transgenic mosquitoes.**

|  | **A-Midgut** | | | **B-Disseminated** | | | **C-Salivary gland** | | |
| --- | --- | --- | --- | --- | --- | --- | --- | --- | --- |
|  | WT | VgDome | VgHop | WT | VgDome | VgHop | WT | VgDome | VgHop |
| **n** | 64 | 58 | 51 | 50 | 64 | 56 | 31 | 39 | 34 |
| **Median** | 2750 | 600 | 450 | 4750 | 600 | 275 | 450 | 0 | 0 |
| **Mean** | 4038 | 1336 | 1290 | 6184 | 2511 | 2515 | 2459 | 840.3 | 911 |
| **SD** | 4989 | 1889 | 1915 | 6578 | 3919 | 3815 | 3277 | 2348 | 2869 |
| **Prevalence (%)** | 85.94 | 72.41 | 74.51 | 96 | 82.81 | 69.64 | 67.7 | 48.7 | 41.2 |

|  | **D-Midgut** | | | | **E-Disseminated** | | | | **F-Disseminated** | | | |
| --- | --- | --- | --- | --- | --- | --- | --- | --- | --- | --- | --- | --- |
|  | WT | VgDome | VgHop | WT | | VgDome | VgHop | Dome x Hop | | WT | VgDome | VgHop |
| **n** | 63 | 66 | 65 | 52 | | 57 | 43 | 47 | | 78 | 77 | 80 |
| **Median** | 14538 | 12741 | 11633 | 200 | | 15 | 0 | 0 | | 22.5 | 0 | 0 |
| **Mean** | 14538 | 12741 | 11633 | 721.2 | | 417.5 | 316.7 | 186.6 | | 2518 | 516.5 | 101.6 |
| **SD** | 19197 | 21745 | 20582 | 1358 | | 1431 | 800.8 | 477.7 | | 10158 | 1839 | 324.7 |
| **Prevalence (%)** | 98.41 | 98.48 | 100 | 78.85 | | 57.89 | 48.84 | 53.19 | | 65.4 | 42.9 | 38.8 |
