# Supplementary material for: Engineered Aedes aegypti JAK/STAT Pathway-Mediated Immunity to Dengue Virus
Source: PLoS Negl Trop Dis. 2017 Jan 12;11(1):e0005187. doi: 10.1371/journal.pntd.0005187 (PMC5230736; doi:10.1371/journal.pntd.0005187)
Supplement: S8 Table — (DOCX) [file pntd.0005187.s013.docx]

**Table S8. Descriptive statistics for DENV infection assays in RF and HF gene silenced mosquitoes.**

|  | **Putative restriction factors** | | | | | | **Putative host factors** | | | |  |
| --- | --- | --- | --- | --- | --- | --- | --- | --- | --- | --- | --- |
| **dsRNA** | GFP | TEP22 | GAMB | UKN7703 | FBN | GFP | | UKN566 | DDX | SCP2 | |
| **n** | 76 | 57 | 66 | 72 | 65 | 69 | | 72 | 60 | 63 | |
| **Median** | 5500 | 4000 | 5000 | 7250 | 4000 | 3500 | | 3000 | 1350 | 500 | |
| **Mean** | 8191 | 14224 | 12293 | 19988 | 13711 | 6616 | | 7263 | 3475 | 1563 | |
| **SD** | 12689 | 27920 | 20234 | 30717 | 29546 | 13707 | | 19046 | 4710 | 3293 | |
